# Pinpointing the axial ligand effect on platinum single-atom-catalyst towards efficient alkaline hydrogen evolution reaction

**DOI:** 10.1038/s41467-022-34619-5

**Published:** 2022-11-12

**Authors:** Tianyu Zhang, Jing Jin, Junmei Chen, Yingyan Fang, Xu Han, Jiayi Chen, Yaping Li, Yu Wang, Junfeng Liu, Lei Wang

**Affiliations:** 1 State Key Laboratory of Chemical Resource Engineering, Beijing University of Chemical Technology, Beijing, 100029 China; 2 Department of Chemical and Biomolecular Engineering, National University of Singapore, Singapore, 117585 Singapore; 3 State Key Laboratory of Acoustics, Institute of Acoustics, Chinese Academy of Sciences Institution, Beijing, 100190 China; 4 Shanghai Synchrotron Radiation Facility, Zhangjiang Laboratory, Shanghai Advanced Research Institute, Chinese Academy of Sciences, Shanghai, 201204 China

**Keywords:** Electrocatalysis, Energy, Nanoscale materials

## Abstract

Developing active single-atom-catalyst (SAC) for alkaline hydrogen evolution reaction (HER) is a promising solution to lower the green hydrogen cost. However, the correlations are not clear between the chemical environments around the active-sites and their desired catalytic activity. Here we study a group of SACs prepared by anchoring platinum atoms on NiFe-layered-double-hydroxide. While maintaining the homogeneity of the Pt-SACs, various axial ligands (−F, −Cl, −Br, −I, −OH) are employed *via* a facile irradiation-impregnation procedure, enabling us to discover definite chemical-environments/performance correlations. Owing to its high first-electron-affinity, chloride chelated Pt-SAC exhibits optimized bindings with hydrogen and hydroxide, which favor the sluggish water dissociation and further promote the alkaline HER. Specifically, it shows high mass-activity of 30.6 A mgPt^−1^ and turnover frequency of 30.3  H_2_ s^−1^ at 100 mV overpotential, which are significantly higher than those of the state-of-the-art Pt-SACs and commercial Pt/C catalyst. Moreover, high energy efficiency of 80% is obtained for the alkaline water electrolyser assembled using the above catalyst under practical-relevant conditions.

## Abstract (web summary)

Establishing robust structure/performance correlations is critical for the development of single-atom-catalysts with improved activity. Here, the axial ligand on Pt single-atom-catalyst is precisely adjusted and studied, showing that the ligand’s first electron affinity is crucial for the catalysis.

## Introduction

Green hydrogen produced via electrocatalytic hydrogen evolution reaction (HER) has been recognized as a promising alternative to mitigate the pressing carbon emission issues^1,2^. Thus, HER has been explored extensively through both proton reduction in acidic media, and water reduction in alkaline media^3,4^. In strong acidic media, noble-metal-based materials are usually needed to catalyse proton reduction for HER to avoid electrode dissolutions^5,6^. In contrast, abundant materials have shown satisfactory stability for water reduction in alkaline electrolyte^7,8^. Unfortunately, the HER kinetics is typically one to a few orders of magnitude lower in alkaline electrolyte than in acids, even on the state-of-the-art platinum (Pt)-based electrocatalysts^9–13^. Besides, HER is also much more sensitive to the catalyst surface-structure in alkaline media than in acids^14^. Nevertheless, enhancing the sluggish kinetics of water reduction in alkaline electrolyte is crucial to reduce the high overpotential and associated energy loss for green hydrogen production^15–17^.

Development of highly active single-atom catalysts (SACs) with maximized atomic utilization efficiency is a promising solution to address the above challenges^18–22^. The chemical environment of the SACs active-sites, including the ligand identity, coordination number and configuration, can directly influence the electronic state, degrees of freedom and other physicochemical properties of the active-center that are associated to the adsorptions of the reactant/intermediates, and thus further determines the catalytic performance of the SACs^23–25^. Hence, revealing the chemical-environment/catalytic-performance correlations is critical for designing SACs with improved activity^26^. Currently, several methods have been developed to synthesize SACs with relatively controllable chemical-environments around the active-center, including defect engineering^27^, annealing^24^, metal-support interaction^28^, heteroatom tethering^29^, cluster/nanoparticle introduction^30^, etc. While tremendous progress has been made in developing new materials using the above synthetic strategies, the harsh conditions involved inevitably break the homogeneity of the active-centers within SACs, e.g., the metal loading, the coordination number/configuration, and the structure of supports, leading complications and uncertainties in establishing definitive correlations between the chemical-environment of SACs active-centers and their catalytic performance^31,32^. Recently, a handful of Pt-SACs have been developed and shown encouraging activity towards alkaline HER^33–38^, however, systematic explorations on stimulating the HER activity via controllable modifications of the chemical-environment around Pt-single-sites is rare. This motivated us to design Pt-SAC systems in which the chemical-environments around the Pt-sites can be precisely manipulated, so that reliable structure/performance correlations can be determined and utilized for future catalyst design.

Herein, we first introduce Pt-single-sites onto NiFe-layered-double-hydroxide (LDH) nanoarrays by electrodeposition, and then develop a facile procedure of irradiation-impregnation to precisely adjust the axial ligand on the Pt- single-sites, based on which we are able to establish robust chemical-environment/HER-activity relationship while maintaining the homogeneity of the above Pt-SACs. Note, during the preparation of our manuscript, another Pt-SAC based on NiFe-LDH, however, with different structure owing to different synthetic method, was reported elsewhere, and in which the axial-ligand-effect was not discussed^39^. In this work, the aforementioned irradiation-impregnation procedure allows us to modify the Pt-sites with different axial ligands, e.g., −F, −Cl, −Br, −I, −OH. Through detailed spectroscopic and electrochemical characterizations, we show that the Cl-Pt/LDH exhibits superior HER performance with a low overpotential of 25.2 mV at 10 mA cm^−2^ in 1.0 M KOH, and a mass activity as high as 30.6 A mgPt^−1^ at the overpotential of 100 mV, which is 5 and 133 times greater than those of the HO-Pt/LDH and commercial 20% Pt/C respectively. Under the same conditions, the HER activity follows the order of Cl-Pt/LDH > F-Pt/LDH > HO-Pt/LDH > Br-Pt/LDH > I-Pt/LDH, confirming the significant axial-ligand effect on HER activity. Density functional theory (DFT) calculations suggest that, owing to the high first-electron-affinity, Cl chelated Pt-sites show optimized adsorption affinities toward both OH* and H*, consequently facilitating the sluggish Volmer step (water dissociation) which is typically the kinetic limiting step for alkaline HER. Moreover, a membrane electrode assembly (MEA) based water electrolyser is assembled using Cl-Pt/LDH and pristine NiFe-LDH as the cathodic and anodic catalysts, respectively. Encouragingly, it exhibits decent performance with a modest cell voltage of 1.87 V at 1 A cm^−2^ at 60 °C, corresponding to a high energy efficiency of 80%. Overall, our efforts demonstrate the significance of engineering the SAC active-sites with atomic precision, the obtained insights can guide next generation catalyst design.

## Results and discussion

### Manipulating the axial ligand on Pt-single-sites

**Fig. 1:**
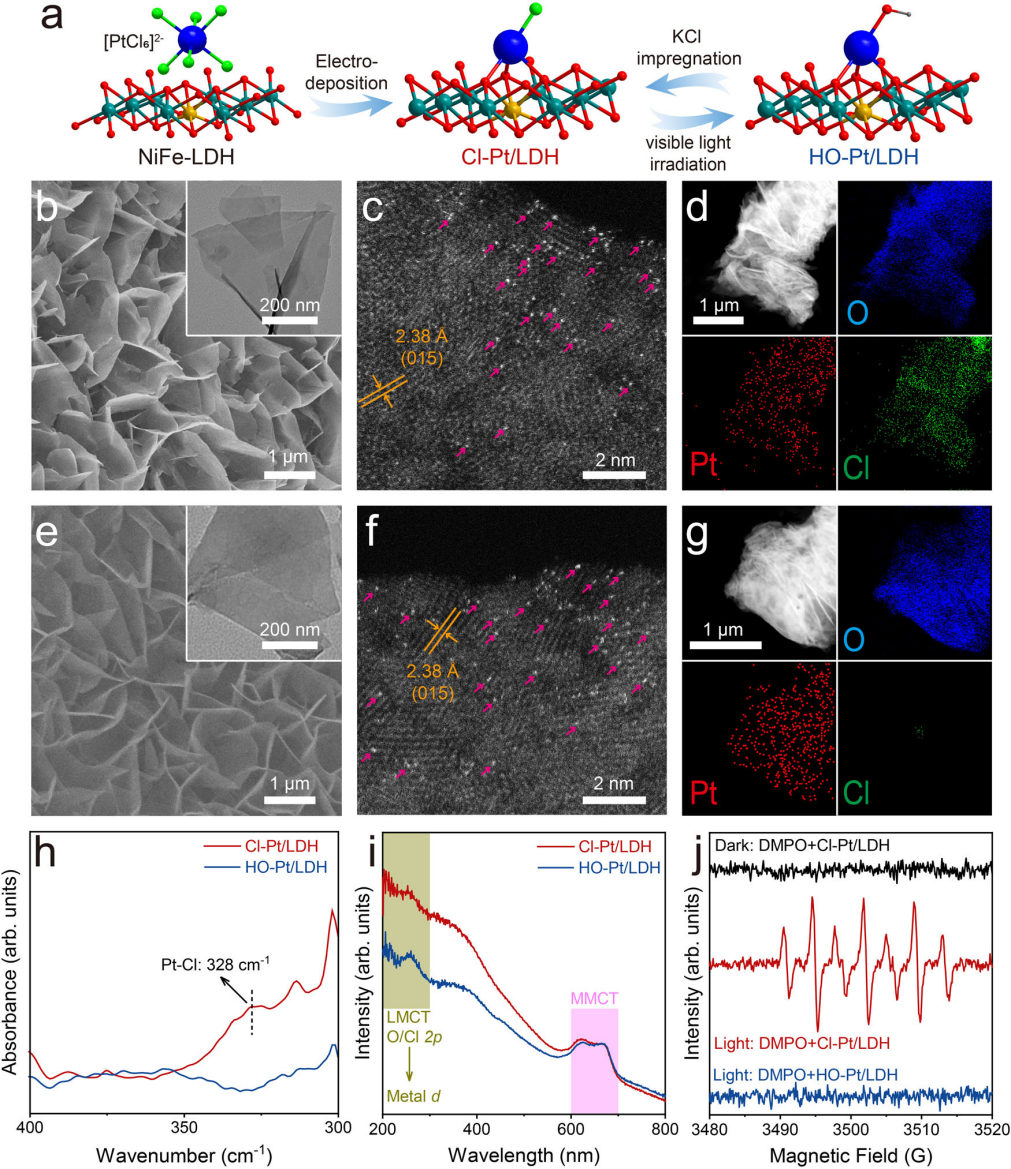
Synthetic procedure and structural characterizations of the Cl-Pt/LDH and HO-Pt/LDH. **a** Schematic illustration of the synthetic and ligand exchange procedure. Atoms are indicated by spheres: Pt (blue), Ni (olive), Fe (yellow), O (red), Cl (green), and H (gray). **b**, **e** SEM (inset: TEM), (**c**, **f**) HAADF-STEM, and (**d**, **g**) corresponding elemental mappings of Cl-Pt/LDH and HO-Pt/LDH, respectively. In Fig. 1c, f, the representative Pt single atoms and lattice spacing are pointed out by the pink arrows and organe arrows, respectively. **h** Far-Infrared and (**i**) UV-vis DRS spectra for Cl-Pt/LDH and HO-Pt/LDH. **j** EPR spectra of Cl-Pt/LDH and HO-Pt/LDH under light/dark conditions.

The irradiation-impregnation procedure for adjusting the axial ligand of Pt/LDH is elaborated in Fig. 1a. First, NiFe-LDH nanosheet arrays were synthesized through a hydrothermal method. Then, PtCl_6_^2−^ anion is adsorbed onto the LDH surface through electrodeposition, forming the atomic dispersed Pt-sites. The as-prepared samples show nearly identical X-ray diffraction (XRD) patterns as the NiFe-LDH support (Supplementary Fig. 1, JCPDS No. 40-0215), suggesting that the crystalline structure of the LDH remains. Scanning electron microscopy (SEM) and transmission electron microscopy (TEM) images show that both the pristine NiFe-LDH and Pt-loaded LDH exhibit plate-like nanosheet array with lateral size of ~500 nm and thickness of ~10 nm (Fig. 1b, e, Supplementary Figs. 2–3), negligible differences in morphology were found. No sub-nanometer clusters or nanoparticles were detected via aberration-corrected high-angle annular dark-field scanning transmission electron microscopy (AC-HAADF-STEM, Fig. 1c), except discrete bright dots, confirming the atomic dispersion of Pt-atoms in Cl-Pt/LDH. Besides, the uniform distributions of Pt and O on the nanoplates (Fig. 1d, g, and Supplementary Fig. 4) suggest that the Pt-atoms are homogeneously dispersed on the LDH substrate. Meanwhile, the lattice fringe with interplanar spacing of about 2.38 Å corresponds to the (015) planes of NiFe-LDH, in line with the XRD results.

To exchange the Cl ligand, we treated the Cl-Pt/LDH with white light irradiation (3.75 mW cm^−2^; 30 min) in KOH. While the atomic dispersion of Pt-atoms remains unchanged after the irradiation (Fig. 1f), the intensity of Cl signal mostly diminished, suggesting the successful removal of Cl ligand. In addition, the decreased signal at 329 cm^−1^ in the Far-Infrared spectra after the irradiation further confirms the loss of the coulombic Pt-Cl bonds (Fig. 1h)^40^. We tentatively attribute the Cl loss to the anion exchange by hydroxide, yielding the product of HO-Pt/LDH, its structure is later determined by X-ray adsorption spectroscopy. Obvious changes were also observed for the ligand-to-metal charge transfer (LMCT) induced absorption bands (~200–300 nm) in Ultraviolet-visible Diffuse Reflectance spectra (UV-vis DRS) of Cl-Pt/LDH and HO-Pt/LDH (Fig. 1i), while the metal-to-metal charge transfer (MMCT) remains unchanged, indicating that only ligand exchange occurred after the irradiation^41^. Electron paramagnetic resonance (EPR) measurements were conducted to study the mechanism of Cl removal^42^. As shown in Fig. 1j, profound radical signals were appeared when exposing the Cl-Pt/LDH to light and the radical scavenger 5,5-dimethyl-1-pyrroline N-oxide (DMPO) spontaneously, which can be attributed to the oxidation of the Cl ligand to Cl radicals by photogenerated holes, which then are captured by DMPO to form DMPO^+●^ radicals^43,44^. In contrast, the absence of radical signals were observed for samples of Cl-Pt/LDH in dark and HO-Pt/LDH under illumination as expected.

Taken together, we conclude that the Cl^−^ to OH^−^ ligand exchange is induced by visible light illumination. Later, the HO-Pt/LDH sample was re-immersed into KCl solution, as expected, the −Cl axial ligand can be recovered (denote as R-Cl-Pt/LDH) due to the strong binding affinity of halogens on Pt. Worth noting, the loadings of Pt in Cl-Pt/LDH, HO-Pt/LDH and R-X-Pt/LDH (X = F, Cl, Br, or I) are very close based on results of Inductively Coupled Plasma Atomic Emission Spectrometer (ICP-AES) (Supplementary Table 1), suggesting that there are no other detectable structural changes occur besides the axial-ligand exchange. Overall, the facile irradiation-impregnation procedure allows us to precisely manipulate the axial ligand of the Pt-single-sites while maintaining the homogeneity of the Pt-SACs.

### Investigating the coordination and electronic structures

**Fig. 2:**
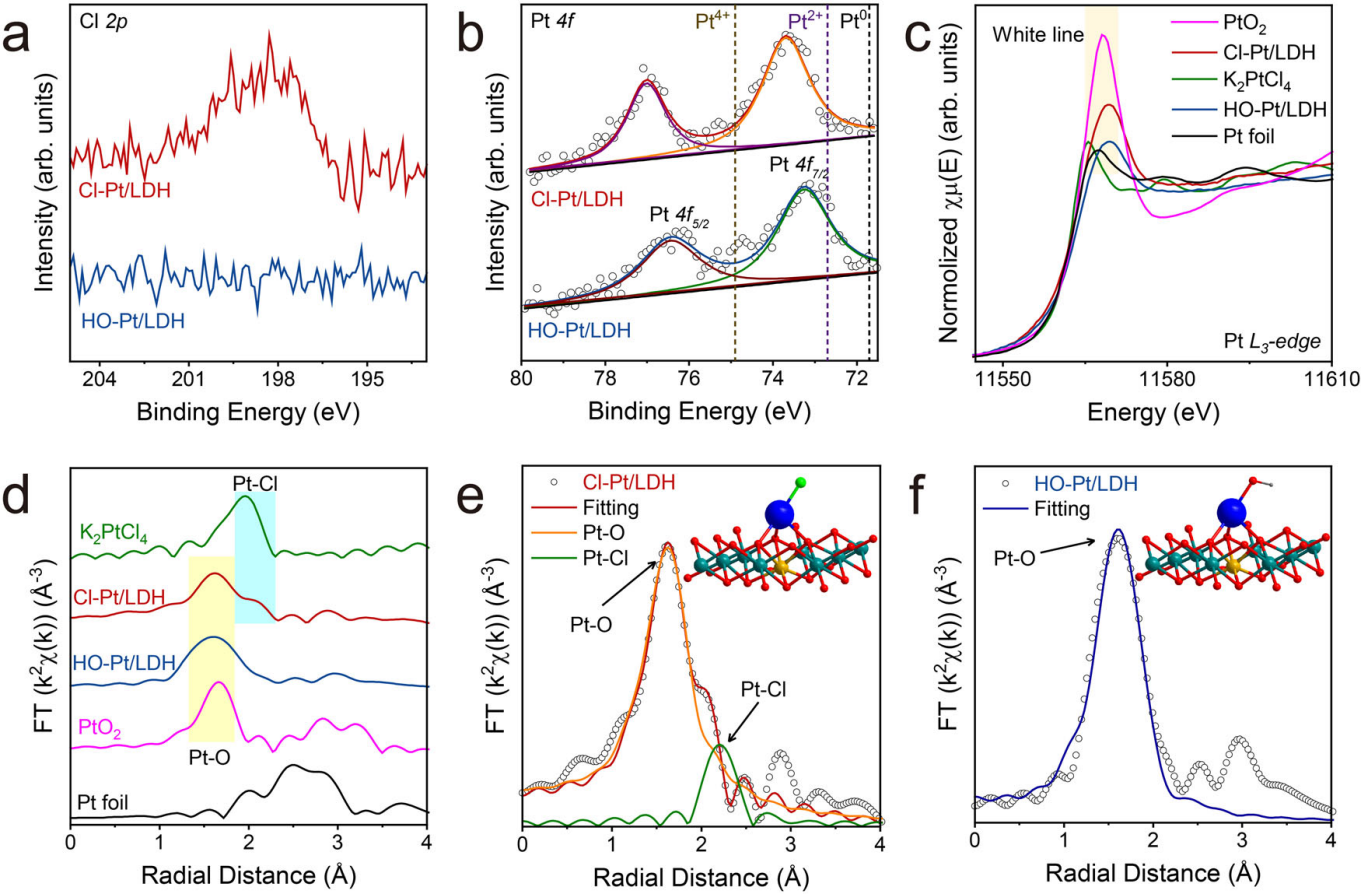
Characterizations of the electronic state and coordinate structure. High resolution XPS spectra of Cl 2p. **a** and Pt *4* *f* (**b**) for Cl-Pt/LDH and HO-Pt/LDH. **c** XANES spectra of Cl-Pt/LDH, HO-Pt/LDH, Pt foil, K_2_PtCl_4_ and PtO_2_ at the Pt L_3_-edge. **d** Corresponding FT-EXAFS spectra. Representative EXAFS fitting curve (inset is the magnified local structure) of Cl-Pt/LDH (**e**) and HO-Pt/LDH (**f**). The blue, olive, yellow, red, green, and gray spheres refer to Pt, Ni, Fe, O, Cl and H atoms, respectively.

X-ray photoelectron spectroscopy (XPS) was employed to analyse the detailed chemical composition of Cl-Pt/LDH and HO-Pt/LDH. The loss of Cl *2p* signal (Fig. 2a) after light irradiation provides further evidence for the complete ligand exchange. Meanwhile, negligible changes can be found in both Fe *2p* and Ni *2p* spectra (Supplementary Fig. 5), indicating that the homogeneity of the Pt-SAC is well retained. As shown in Fig. 2b, the *4f*_*7/2*_ peaks of Pt-atoms in both Cl-Pt/LDH and HO-Pt/LDH are located between Pt^2+^ (72.7 eV) and Pt^4+^ (74.9 eV), indicating the average valence states of these Pt-atoms are between +2 and +4^45^. The slightly higher binding energy of the Pt *4f-*electrons in Cl-Pt/LDH than that of the HO-Pt/LDH agrees well with previous results where electron transfer was observed from Pt to the Cl ligand^46^.

X-ray absorption fine structure (XAFS) spectroscopy was performed to investigate the coordination environment and electronic structure of Pt-single-sites in Cl-Pt/LDH and HO-Pt/LDH. First, the X-ray absorption near edge structure (XANES) spectra of the above samples and the references of Pt foil, K_2_PtCl_4_, and PtO_2_ at the Pt L_3_-edge were analysed. The white line at the Pt L_3_-edge (the intensity of which is a measure of the transition of *2p*_*3/2*_ → *5d*_*3/2*_ or *5d*_*5/2*_^47^) was recognized as a reliable indicator for the Pt electronic structure, since the unoccupied states above the Fermi level are essentially the *5d* character of Pt^48,49^. As shown in Fig. 2c, the white lines of both Cl-Pt/LDH and HO-Pt/LDH locate between those of the K_2_PtCl_4_ (Pt^2+^) and PtO_2_ (Pt^4+^), suggesting that the valence states of Pt-atoms in Cl-Pt/LDH and HO-Pt/LDH are between +2 and +4, in line with the above XPS results. After the curve fitting using standard references, the valence states of Pt in Cl-Pt/LDH and HO-Pt/LDH are estimated as 2.78 and 2.08, respectively (Supplementary Fig. 6a), illustrating the existence of strong ligand effects on the electronic structure of Pt-SACs.

Fourier-transformed (FT) *k*^2^-weighted extended X-ray absorption fine structure (EXAFS) spectra were employed to further analyse the coordination environment of the Pt-SACs. As shown in Fig. 2d, no Pt-Pt interactions at ~2.50 Å can be detected in both Cl-Pt/LDH and HO-Pt/LDH, demonstrating the atomic dispersion of Pt-atoms in these samples, which is consistent with the CO chemisorption results from the Fourier-transformed Infrared Spectroscopy (Supplementary Fig. 6b)^20^. Besides, two obvious peaks at around 1.60 Å and 2.06 Å are found in Cl-Pt/LDH, which are associated to the Pt-O path in PtO_2_ and the Pt-Cl path in K_2_PtCl_4_, respectively. In contrast, only one Pt-O like peak at ~1.60 Å can be seen in HO-Pt/LDH, indicating that the Cl ligand is fully replaced after irradiation. Based on the wavelet transform (WT) analysis of the EXAFS spectra (Supplementary Fig. 7), Cl-Pt/LDH displays a higher WT maximum at 5.2 Å^−1^ than that of HO-Pt/LDH at 4.9 Å^−1^, suggesting that the average coordination number of the Pt-sites in Cl-Pt/LDH is larger than that in HO-Pt/LDH.

To reveal the chemical environment of the Pt-single-sites, we performed quantitative least-square EXAFS curve-fitting analysis for the Pt-SACs. Several typical structures of LDH based SACs are fitted and modeled (Supplementary Fig. 8). Among them, the Pt-site chelated by three surface O-atoms on the LDH layer and one axial ligand opposite of the Fe-atom is considered to be the most possible structure (Fig. 1a and Supplementary Fig. 8a). The fiducial distances between Pt and coordinated atoms were calculated using standard crystal structures of Pt foil, K_2_PtCl_4_, and PtO_2_ (Supplementary Fig. 9-11). The best-fitted results of Cl-Pt/LDH (Supplementary Table 2) suggest that the main peak at 1.60 Å in the FT-EXAFS spectra at the Pt L_3_-edge can be attributed to the Pt-O first coordination sphere, whereas the minor peak at 2.06 Å can be attributed to the Pt-Cl first coordination sphere (Fig. 2e and Supplementary Fig. 12). Based on the EXAFS fitting parameters for Cl-Pt/LDH at the Pt L_3_-edge, O-atoms within the first coordination sphere is located at 2.02 Å with a coordination number of 3.02, suggesting a tetrahedron geometry for the Pt-sites. Besides, the Cl-atom at 2.29 Å with an estimated coordination number of 0.93 is proposed to be a vertical ligand on Pt. All things considered, we propose the most possible structure of the Cl-Pt/LDH as shown in the inset of Fig. 2e. Similar analysis on HO-Pt/LDH demonstrates that it only has O atoms in the first coordination sphere with a total coordination number of 3.60, affirming that the Cl ligand is replaced by the hydroxide group (Fig. 2f) with no other changes occurred to the Pt-sites.

### Electrocatalytic alkaline HER on Pt-SACs

**Fig. 3:**
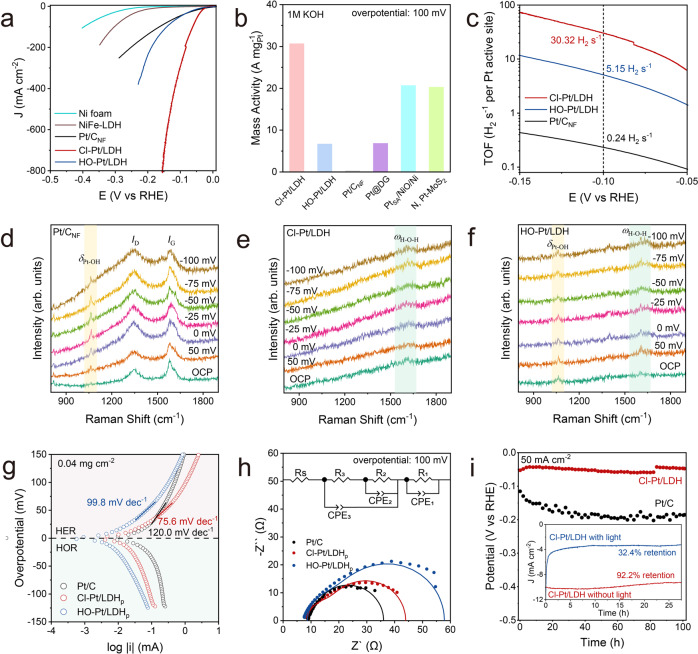
Alkaline HER. **a** HER polarization curves of Ni foam, NiFe-LDH, Pt/C, Cl-Pt/LDH, and HO-Pt/LDH. **b** Mass activities of the Pt-based catalysts in this work, and the state-of-the-art SACs reported elsewhere: Pt@DG^37^, Pt_SA_/NiO/Ni^34^ and N, Pt-MoS_2_^50^. **c** TOFs plots of the Pt-based catalysts. *Operando* Raman spectrum of (**d**) Pt/CNF, (**e**) Cl-Pt/LDH and (**f**) HO-Pt/LDH. **g** Micro-polarization curves for Pt/C, Cl-Pt/LDH_p_ and HO-Pt/LDH_p_. (**h**) EIS Nyquist plots of the Pt-based catalysts. **i** Plot of potential *vs*. time for the Cl-Pt/LDH and commercial Pt/C at a constant cathodic current density of 50 mA cm^−2^. (inset is chronoamperometry stability tests of Cl-Pt/LDH under the condition with or without the light irradiation.) The Pt loading amount of Cl-Pt/LDH, HO-Pt/LDH, Pt/C_NF_, Cl-Pt/LDH_p_, HO-Pt/LDH_p_, and Pt/C is 1.01 × 10^−2^, 1.01 × 10^−2^, 0.4, 2.88 × 10^−4^, 2.88 × 10^−4^ and 8.00 × 10^−3^ mgPt cm^−2^, respectively.

The electrocatalytic HER on Cl-Pt/LDH is examined in H_2_-saturated KOH (1.0 M). For comparison, the HER activities of Ni foam, NiFe-LDH, 20% Pt/C deposited on Ni foam (Pt/C_NF_) and HO-Pt/LDH are also measured under the same conditions. As shown in Fig. 3a, Cl-Pt/LDH exhibits the superior HER activity and ~100% Faradic efficiency to H_2_ among all the Pt-SACs tested here (Supplementary Fig 13 and Table 3), with modest overpotentials of 25.2 mV, 51.9 mV, and 72.3 mV to achieve the current densities of 10 mA cm^−2^, 100 mA cm^−2^, and 200 mA cm^−2^, respectively, overperforming both Pt/C_NF_ (27.4 mV, 164.9 mV, and 252.0 mV) and HO-Pt/LDH (41.5 mV, 142.5 mV, and 189.5 mV). The negligible HER activities of pristine Ni foam and the SCN^−^ poisoning experiment (Supplementary Fig. 14) both indicate that the high HER activity of Cl-Pt/LDH is originated from the Pt-sites. Further increase in the Pt loading leads to the formation of Pt nanoparticles (Pt_np_/LDH) and decrease in activity (Supplementary Fig. 15). The mass activity of Cl-Pt/LDH normalized to the Pt loading at overpotential of 100 mV is estimated as 30.6 A mgPt^−1^ (Fig. 3b), significantly larger than those of HO-Pt/LDH (6.6 A mgPt^−1^), Pt/C_NF_ (0.2 A mgPt^−1^), and the state-of-the-art Pt-SACs reported elsewhere (Pt@DG^37^, 6.78 A mgPt^−1^, Pt_SA_/NiO/Ni^34^, 20.6 A mgPt^−1^, and N, Pt-MoS_2_^50^, 20.2 A mgPt^−1^). Moreover, the turnover frequencies (TOFs) per Pt-site on Cl-Pt/LDH (30.3 H_2_ s^−1^) at overpotential of 100 mV are 5.9 and 126 times greater than those of HO-Pt/LDH (5.1 H_2_ s^−1^) and the commercial Pt/C_NF_ (0.2 H_2_ s^−1^), respectively (Fig. 3c). Steady-state cyclic voltammetry (CV) in full regions of water splitting is performed to demonstrate the superior performance of Cl-Pt/LDH (Supplementary Figs. 16–17). We then normalized the HER activities by the corresponding ECSAs estimated by measuring double-layer capacitance (C_dl_) at non-faradic regions, it turns out that Cl-Pt/LDH remains as the most active catalyst (Supplementary Fig. 18).

### Kinetics and intrinsic activities of the Pt-SACs for alkaline HER

*Operando* Raman spectroscopy was performed to probe the surface species and their changes in chemical bonding during HER. As shown in Fig. 3d, only Pt-OH peaks at 1062 cm^−1^ appeared for HER on Pt/C_NF_^51,52^. We therefore propose that the desorption of *OH is slow under the catalytic conditions, which agrees with previous observations suggesting that *OH is not a spectator in alkaline HER^9,53,54^. In contrast, such obvious Pt-OH peaks were not observed on Cl-Pt/LDH (Fig. 3e) under the same conditions. Instead, only subtle peaks at ~1630 cm^−1^ were found, which can be attributed to the H−O−H bending mode of the adsorbed water, suggesting the Volmer step and the consequent *OH desorption steps are accelerated, resulting in enhanced HER kinetics^55–57^. The subtle peak for adsorbed water exists in the HO-Pt/LDH as well, in line with the enhanced activity compared with that of Pt/C. Note, while minor Pt-OH peaks were also found in HER on HO-Pt/LDH (Fig. 3f), its intensity did not vary along with the changes in overpotential, suggesting the −OH is the axial ligand instead of coming from the water reduction. Overall, we conclude that the improved HER activity on the Pt-SACs is likely originated from the accelerated Volmer step. However, we note that due to the limitations of the operando spectroscopy, we do not exclude the effect of mass transportation during the measurements.

Tafel analysis was carried out to investigate the reaction kinetics of HER. As shown in Supplementary Fig. 19, Cl-Pt/LDH exhibits a low apparent Tafel slope of 24.33 mV dec^−1^, corresponding to a Tafel step limited HER kinetics. This is counterintuitive since Volmer step is usually determined as the rate-determine-step (RDS) for alkaline HER^58,59^. We thus believe that the HER on Cl-Pt-LDH is largely limited by the mass transportation of H_2_ leaving the electrode surface in the above measurements, resulting uncertainties for investigating its intrinsic activities^60^. Note, the Pt-SACs may be affected by the H_2_ mass diffusion to a different extent due to their different gas affinities (Supplementary Fig. 20). To evaluate the intrinsic HER activities of the above catalysts, powder-based catalysts (X-Pt/LDH_p_) were prepared and tested on a rotating disk electrode (RDE, Supplementary Fig. 21) to eliminate the influence of the H_2_ mass transportation, at least to the best extent. The Tafel slope obtained for Cl-Pt/LDH_p_ increases significantly with decreased catalyst loadings (Fig. 3f and Supplementary Fig. 21), suggesting that the mass transport effect become less dominant with low catalyst loading, at least within the potential window of interest^5^. Note, similar activity trends remain (Supplementary Fig. 22) even with extremely low catalyst loadings (0.04 mg cm^−2^), confirming the enhanced intrinsic activity of Cl-Pt/LDH compared to other samples. Under this condition, Cl-Pt/LDH_p_ shows a much lower Tafel slope (75.6 mV dec^−1^) compared to those of HO-Pt/LDH_p_ (99.8 mV dec^−1^) and Pt/C (120.0 mV dec^−1^), indicating that the sluggish Volmer step is significantly boosted on Cl-Pt/LDH_p_, and the RDS is likely the mixture of Volmer and Heyrovsky steps (Fig. 3g)^61^. Note that, the difference in the symmetry of the log *J* vs. *E* curves between Cl-Pt/LDH_p_ and HO-Pt/LDH_p_ suggest that *OH is an active participant in the hydrogen reactions via Volmer-Heyrovsky mechanism^62^.

Electrochemical impedance spectroscopy (EIS) was also employed to investigate the HER kinetics. As shown in Fig. 3h, the first semicircle at medium frequency is believed to be associated to the kinetics of Volmer step (Supplementary Fig. 23)^63^. Apparently, Cl-Pt/LDH_p_ shows a significant lower adsorption resistance (R_2_ = 21.15 ohm) than HO-Pt/LDH_p_ (R_2_ = 28.45 ohm), indicating that the water dissociation step is more favored on Pt-sites with Cl as axial ligand^15,39,64^. In addition, in-situ Cl ion titration were conducted to reveal the importance of the axial Cl-ligand to the HER activity. As shown in Supplementary Fig. 24, along with the increase of irradiation period, the Cl: Pt mole-ratio decreased from 1.08 to 0.05 accompanied by the decline of HER activity, suggesting that −Cl is replaced by hydroxide, consisting with the above XAFS results. The ligand exchange occurred quickly under the catalytic conditions. As shown in the inset of Fig. 3i, the HER activity of Cl-Pt/LDH dropped and reached to a plateau within the first half-hour under illumination, suggesting the completion of the ligand exchange within this short period. In contrast, Cl-Pt/LDH retained its activity when light was blocked from the catalytic system, indicating that the −Cl axial ligand on Pt is relatively stable under the HER conditions. Physical characterizations also suggest that its morphological and electronic structures were retained (Supplementary Figs. 25–26). Moreover, extended stability tests were conducted for both Cl-Pt/LDH and Pt/C at current densities of 50 mA cm^−2^ (Fig. 3i) and 500 mA cm^−2^ (Supplementary Fig. 27) under the same conditions. Over the 100-hour testing period, Cl-Pt/LDH exhibited improved durability compared to the commercial Pt/C catalyst, demonstrating the robust chemical structure of the Pt-single-sites under alkaline HER conditions.

### Exploring the origin of the axial-ligand effect

**Fig. 4:**
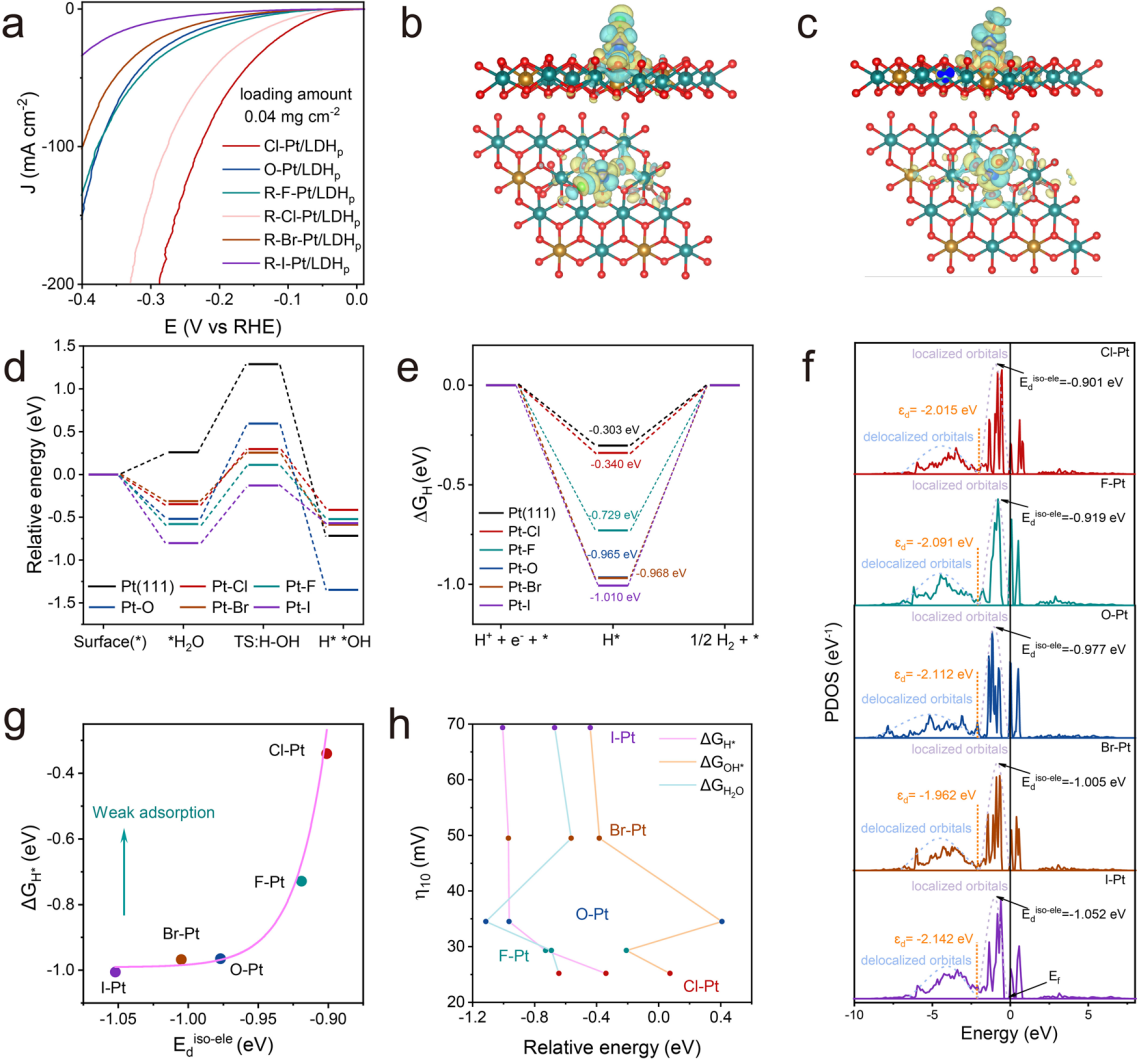
Axial-ligand effect and theoretical investigations. **a** HER polarization curves of the Pt-SACs with different axial-ligands. Computational models and localized electric field distributions of (**b**) Cl-Pt/LDH and (**c**) HO-Pt/LDH. **d** Calculated energy barriers of water dissociation kinetics and (**e**) adsorption free energies of H* on the surfaces of the Pt-SACs, and the Pt (111) slab as a reference. **f** Calculated Pt 5d band structures of the Pt-SACs. **g** Relationship between the E_d_^iso-ele^ and ΔG_H*_. **h** Relationship between alkaline HER overpotentials (at 10 mA cm^−2^) and variables. The Pt loading amount of Cl-Pt/LDH_p_, HO-Pt/LDH_p_, R-F-Pt/LDH_p_, R-Cl-Pt/LDH_p_, R-Br-Pt/LDH_p_ and R-I-Pt/LDH_p_ is 2.88×10^−4^, 2.88 × 10^−4^, 2.84 × 10^−4^, 3.00 × 10^−4^, 2.92 × 10^−4^, and 2.92 × 10^−4^ mgPt cm^−2^, respectively.

We extended the axial-ligand identity using the above irradiation-impregnation procedure to prepare R-F-Pt/LDH, R-Br-Pt/LDH, and R-I-Pt/LDH. HAADF-STEM images and the corresponding elemental mappings (Supplementary Figs. 28–31) confirmed the desired axial-ligand exchange on Pt-sites. XPS spectra (Supplementary Fig. 32) indicate that the valence state changes only occur to the Pt-atoms instead of the NiFe-LDH supports. All in all, we believe the homogeneity of the chemical environment around the Pt-sites of these catalysts remain after the ligand exchange. Note, at first glance (Fig. 4a and Supplementary Figs. 33–34), the alkaline HER activities of the Pt-SACs with different axial-ligands scale with the first-electron-affinity of the halogen-atoms. In the following we tried to explore the physical insights of this axial-ligand effect.

First, EIS measurements were carried out to investigate the detailed HER kinetics on these catalysts^65,66^. Based on the equivalent circuit (Supplementary Fig. 23), R_2_ and R_3_ are associated to the Volmer step and Heyrovsky step, respectively^15,39,63,64^. On Pt/C, the phase angle of the Heyrovsky step decreased rapidly while the phase angel of the Volmer step remain largely unchanged when increasing the overpotential for HER (Supplementary Fig. 35). In addition, R_2_ remains larger than R_3_ at all overpotentials, indicating the RDS for HER on Pt/C is always the Volmer step under the testing condition (Supplementary Fig. 36). On a contrary, phase angels of both the Volmer and Heyrovsky steps on Pt-SACs decrease along with the increase of overpotential, and R_2_ is mostly smaller than R_3_, suggesting that the Volmer step has been accelerated on the Pt-SACs consisting with the previous results (Supplementary Fig. 37 and Supplementary Tables 4–9).

First principal simulations were also performed to rationalize the axial-ligand effect on Pt-SACs for alkaline HER. The computational models for X-Pt/LDH (X = −F, −Cl, −Br, −I, and −OH) were built based on the EXAFS fitting results. As shown in Fig. 4b, c and Supplementary Figs. 38, 39, significant charge redistributions were observed at the Pt-X bonding regions, which is likely the origin of the enhanced HER activity. Stepwise reaction barriers for alkaline HER on Pt-SACs were modeled, including the Volmer step of water dissociation, *OH desorption, and the subsequent conversion of H* to H_2_. For the Volmer step (Fig. 4d), all the halogen-coordinated Pt/LDHs show stronger H_2_O adsorption ability and larger formation of enthalpy for water dissociation than those on Pt (111), leading to accelerated Volmer step. Among them, Cl-Pt/LDH exhibits the smallest energy barrier (0.073 eV) for the Volmer step. On another hand, the Cl-Pt-sites also exhibit the most optimized H binding energy among the Pt-SACs, leading to the most favored kinetics for the conversion of H* to H_2_ (Fig. 4e). Note, while Pt (111) shows slightly more optimized H binding energy compared to that of the Cl-Pt/LDH, its water dissociation step is expected to be slow (Fig. 4e), leading to a slower overall HER kinetics. The combined results indicate that the H binding energy is not the sole descriptor for alkaline HER, OH binding energy also plays significant role as it is likely not a spectator for the reaction^67–69^. The projected density of states (PDOS) of the *d*-orbitals of the Pt-SACs were calculated as well (Supplementary Fig. 40). Subsequently, the average energy levels of the narrow Pt*−5d* orbitals occupied by isolated electrons (E_d_^iso-ele^) were also calculated for Cl-Pt, F-Pt, HO-Pt, Br-Pt, and I-Pt as −0.901, −0.919, −0.977, −1.005, and −1.052 eV, respectively (Fig. 4f). This is in good agreement with the above XANES results, where the highest Pt valence state was observed for Pt in Cl-Pt/LDH owing to the largest first-electron-affinity of Cl ligand. According to the d-band theory, the adsorption properties of the surface intermediates are directly associated to the electronic structure of catalyst^70,71^. Increased first-electron-affinity of the ligands on Pt-single-atoms is expected to increase the E_d_^iso-ele^, and further weaken the hydrogen adsorption. Cl-Pt with the highest E_d_^iso-ele^ should yield the weakest interaction with H* intermediate, consequently H* is more readily to desorb and form H_2_ (Fig. 4g). Overall, the computational results agree with our experimental observations, demonstrating that modifying the electronic structure of the Pt-single-sites with axial ligand is an effective strategy in tuning the HER activity.

We further conducted CO stripping experiments to evaluate the *OH binding strength on Pt-SACs, since one could expect that strong Pt-OH bond strength will facilitate the removal of *CO owing to the adsorbate-adsorbate interactions^33,52^. As shown in Supplementary Figs. 41–42, a volcano-type relationship between alkaline HER activity and Pt-*OH strength was observed, indicating both *OH adsorption and desorption are important for alkaline HER. Taken together, we show that the alkaline HER activity of X-Pt/SACs increase monotonically with the ligand first-electron-affinity, as both *H and *OH are the dominant descriptors during alkaline HER (Fig. 4h).

### HER performance in an industrial relevant reactor

**Fig. 5:**
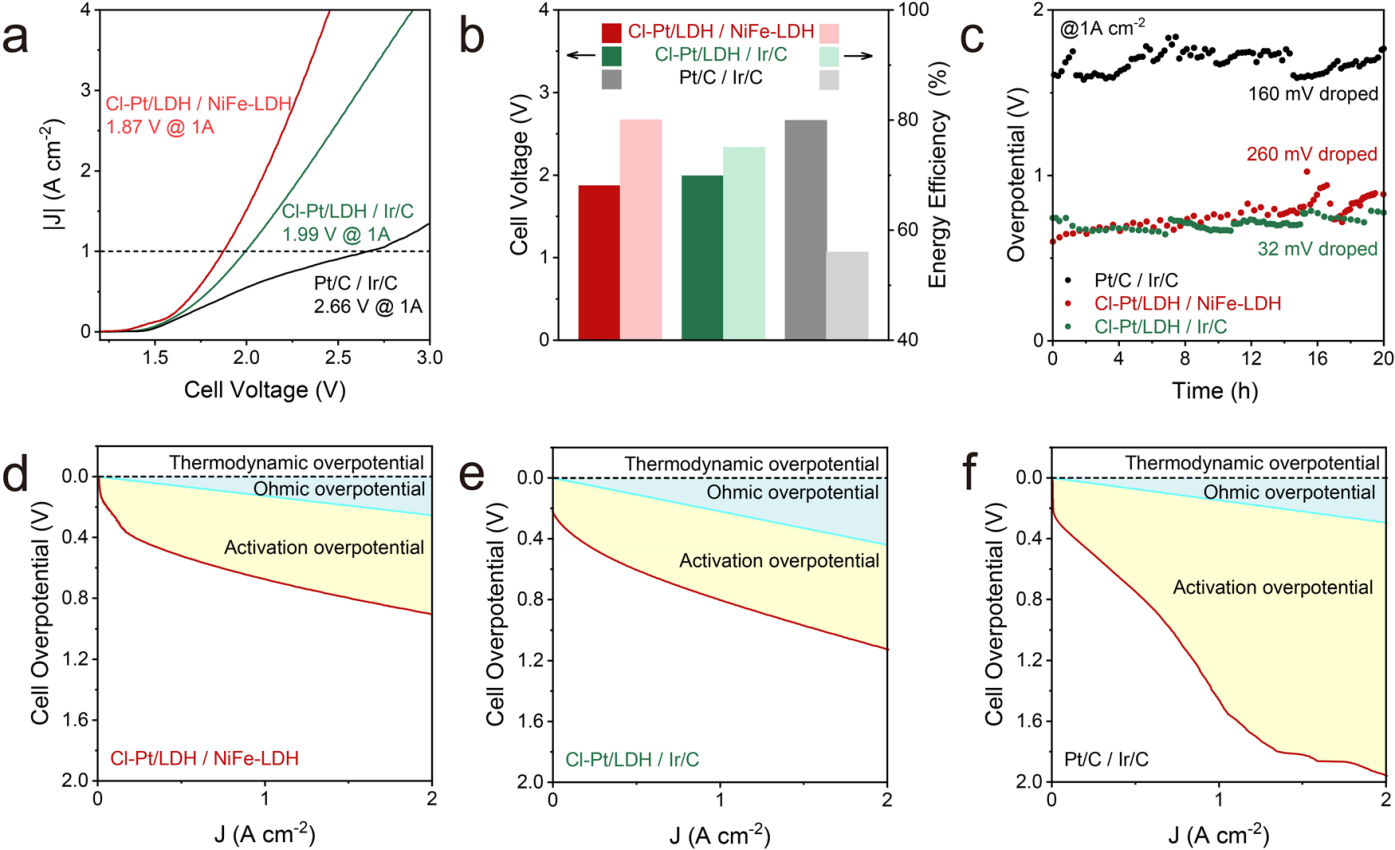
Alkaline HER in the MEA electrolyser. **a** LSV curves of the MEA reactors using Cl-Pt/LDH / NiFe-LDH, Cl-Pt/LDH / Ir/C, and commercial Pt/C / Ir/C as the cathodic and anodic electrodes, respectively. **b** Cell voltage at current density of 1 A cm^−2^ in Fig. 5a, and the corresponding energy efficiency. **c** Stability tests of the MEA water electrolysers at the current density of 1 A cm^−2^ at 60 °C. Breakout of the ohmic overpotential and activation overpotential for (**d**) Cl-Pt/LDH / NiFe-LDH, (**e**) Cl-Pt/LDH / Ir/C and (**f**) commercial Pt/C / Ir/C. The Pt loading of Cl-Pt/LDH, and Pt/C is 1.01 × 10^−2^ and 0.8 mgPt cm^−2^, respectively.

To evaluate the applicability of Cl-Pt/LDH as a cathodic catalyst under practical relevant operating conditions, we constructed an anion exchange membrane-based membrane electrode assemblies (MEA) electrolyser by using the pristine NiFe-LDH as the anode. MEA electrolysers based on commercial Pt/C and Ir/C were built and tested for comparison. The current-voltage profiles were obtained under 60 °C (Fig. 5a). Without further optimizations (reactor design, membrane choice and operating conditions, etc.), the electrolyser based on Cl-Pt/LDH / NiFe-LDH exhibits a much lower cell voltage (1.87 V) than those of the Cl-Pt/LDH / Ir/C (1.99 V) and the Pt/C / Ir/C (2.66 V) based electrolyser at current density of 1.0 A cm^−2^. Under this condition, their corresponding energy conversion efficiencies are 80%, 75% and 56%, respectively (Fig. 5b). The electrolysers were also subjected to water electrolysis for a 20 h-long stability tests at 1.0 A cm^−2^. As shown in Fig. 5c, the Cl-Pt/LDH / Ir/C electrolyser exhibits good stability with negligible overpotential loss (Supplementary Fig. 43), demonstrating promising potentials for future implementation for green hydrogen production. As shown in Fig. 5d–f, it is obvious that the activation overpotentials of Cl-Pt/LDH / NiFe-LDH or Cl-Pt/LDH / Ir/C are much lower that of Pt/C / Ir/C, leading to the enhanced energy efficiency originated from the superior HER activity of Cl-Pt/LDH.

In summary, we present Pt-SACs anchored on NiFe-LDH nanoarrays with different axial ligands (−F, −Cl, −Br, −I, and −OH) as highly active alkaline HER electrocatalysts prepared by a facile irradiation-impregnation procedure. Cl-Pt/LDH exhibits the highest HER activity among the Pt-SACs as well as the commercial Pt/C. The enhanced intrinsic activity of Cl-Pt/LDH towards alkaline HER is attributed to the largest first electron affinity of the axial ligand which poses strong electronic effect on the Pt-single-sites. DFT calculations suggest that the introduction of axial ligand on Pt-single-sites can tune the average energy levels of the isolated electrons occupied Pt-d-orbitals, and further the H* and *OH adsorption energies. Finally, Cl-Pt/LDH was also evaluated in a MEA-based alkaline water electrolyser at industrially relevant reaction rates, energy efficiency as high as 80% was obtained, demonstrating its promising application for hydrogen production. Overall, our work highlights the benefits of tailoring the chemical environments around the catalytic center, both the synthetic strategy and the constructive axial-ligand effect reported here can guide future SACs design with improved performance for large-scale green hydrogen production.

## Methods

### Chemicals and materials

Nickel (II) nitrate hexahydrate (Ni(NO_3_)_2_·6H_2_O), iron (III) nitrate nonahydrate (Fe(NO_3_)_3_·9H_2_O), Hexadecyltrimethylammonium bromide (CTAB), chloroplatinic acid hydrate (H_2_PtCl_6_·xH_2_O), potassium thiocyanate (KSCN), sodium fluoride (NaF), sodium chloride (NaCl), sodium bromide (NaBr), sodium iodide (NaI), hydrochloric acid (HCl), 5,5-Dimethyl-1-pyrroline N-oxide (DMPO), Nafion 117 containing solution (~5% in a mixture of lower aliphatic alcohols and water) were purchased from Sigma-Aldrich. Commercial Pt/C (20 wt.% Pt on carbon) and Ir/C (20 wt.% Ir on carbon) were purchased form Alfa Aesar. Potassium hydroxide (KOH, 99.99%) was purchased from Shanghai Macklin Biochemical Co., Ltd). Nickel foam, carbon fiber paper (Toray 060), and Dioxide Materials Sustainion membrane (X37-60 grade T) were purchased from Suzhou sinero technology Co., LTD. Ethanol (EtOH) and methanol (MeOH) were purchased from Beijing Chemical Works. All water were prepared with OmniaPure ultra-pure water system (resistivity of 18.2 MΩ cm). All chemical reagents were used as received without further purification.

### Synthesis of NiFe-LDH

The nickel-iron layered double hydroxide (LDH) nanosheet arrays in-situ growing on nickel foam were synthesized through a facile one-pot solvothermal method. Typically, 0.9 mmol Ni(NO_3_)_2_·6H_2_O and 0.6 mmol Fe(NO_3_)_3_·9H_2_O and 1.4 mmol CTAB were dissolved in a mixture of 36 mL MeOH and 6 mL water and stirred to form a clear solution. Nickel foam (about 4 × 3 cm) was carefully cleaned with the concentrated HCL solution (37 wt.%) in an ultrasound bath for 5 min to remove the surface NiO layer, and then water and MeOH were used for 5 min to ensure the surface of Ni foam was cleaned. Both the Ni foam and the solution were transferred to a 50 mL Teflon-lined stainless-steel autoclave. The as-prepared Ni foam was vertically immersed into the solution. The in-situ growth was carried out in an oven for 24 h at 180 °C. After the naturally cooling to the room temperature, a thin brown film on the Ni foam was formed, donated as NiFe-LDH. Subsequently, the NiFe-LDH was rinsed with water and MeOH for 3 min with the assistance of ultrasonication, and then dried at 80 °C overnight. The average loading amount of LDH on Ni foam is 1.4 mg cm^−2^. Besides, the powder-based catalysts were also collected by scraping the deposited materials on Ni foam, donated as NiFe-LDH_p_.

### Synthesis of Cl-Pt/LDH

The Cl-Pt/LDH was synthesized through electrochemical deposition in a three-electrode system, in which NiFe-LDH was used as the working electrode, graphite rod acted as the counter electrode, and the mercury/mercury oxide (Hg/HgO) electrode was employed as the reference electrode. The NiFe-LDH on Ni foam was cut into small size to afford the geometric area of the working electrode to be 1 cm^2^. The NiFe-LDH was firstly activated by cyclic voltammetry (CV) with a scan rate of 10 mV s^−1^ between 0.05 and −0.50 V versus reversible hydrogen electrode (RHE) for 30 cycles in 1 M KOH. Subsequently, the system was transferred into 1 M KOH solution containing 0.15 mmol L^−1^ H_2_PtCl_6_ for further electrodeposition of Pt single atoms. The Cl-Pt/LDH was fabricated by CV with a scan rate of 5 mV s^−1^ between 0.05 and −0.50 V versus RHE for 40 cycles. The Pt/C on Ni foam was fabricated by drop casting with a loading of 2 mg cm^−2^. The powder-based Cl-Pt/LDH_p_ was fabricated based on NiFe-LDH_p_ by CV with a scan rate of 50 mV s^−1^ between 0.05 and −0.70 V versus RHE for 20 cycles, this adjustment was made due to the different electrode surface area. When testing powder-based NiFe-LDH_p_ and corresponding X-Pt/LDH_p_, glassy carbon electrode with geometric area of 0.196 cm^−2^, and different catalyst loading (0.1 mg cm^−2^ or 0.004 mg cm^−2^) was used. All electrochemical curves were iR-corrected according to the equation: E_iR-correctred_ = E – I × R_s_.

### Synthesis of HO-Pt/LDH

The Cl ligand can be removed and replaced by the hydroxide group under the irradiation of visible light in water. PLS-SXE300 from Beijing perfectlight with a spectral region from 320 nm to 780 nm was used as the light source with an output power density of 3.75 mW cm^−2^. After 30 min irradiation, the HO-Pt/LDH was fabricated after the washing and drying process. The HO-Pt/LDH_p_ was synthesized via a similar procedure using Cl-Pt/LDH_p_.

### Synthesis of R-X-Pt/LDH (X = F, Cl, Br, or I)

The axial-ligand on Pt-single-atom can be easily reversed from hydroxide group to halogen by simple impregnation strategy. In a typical process, the R-F-Pt/LDH was fabricated by immersed HO-Pt/LDH in 2 M NaF solution at 40 °C for 4 h under magnetic stirring. Similarly, R-Cl-Pt/LDH, R-Br-Pt/LDH and R-I-Pt/LDH can be synthesized by changing the solution into NaCl, NaBr, and NaI, respectively. All the reversed catalysts were activated by two CV cycles at HER region in 1 M KOH in absence of light irradiation before elemental mapping and XPS measurements to remove the influence of possible anion intercalation in LDH supports. The R-X-Pt/LDH_p_ was synthesized via the same procedure using HO-Pt/LDH_p_.

### Synthesis of Pt_np_/LDH

The fabrication process of Pt_np_/LDH was similar with that of Cl-Pt/LDH however with increased CV cycles (100 cycles).

### Characterizations

Field-emission scanning electron microscopy (FESEM) images were taken on a Zeiss SUPRA 55. The transmission electron microscopy (TEM) images were carried out by a Hitachi HT7700 transmission electron microscopy. The crystal structures of the samples were examined by X-ray diffraction (XRD, Bruker D8) with Cu Kα radiation (λ = 0.154178 nm). High-resolution TEM (HR-TEM) and the corresponding energy-dispersive X-ray (EDX) mapping were taken on a JEOL JEM-2100F microscope. Aberration-corrected high-angle annular dark field scanning transmission electron microscopy (AC-HAADF-STEM) was performed on a JEM-ARM 200 F TEM/STEM with a spherical aberration corrector. All samples for TEM measurements were prepared by ultrasonic dispersion in ethanol and were drop-casting onto copper grids covered with a carbon film. Raman spectra were recorded on a Lab Ram ARAMIS Raman spectrometer (HORIBA) at an excitation wavelength of 633 nm. UV-vis absorption spectra were collected on a Shimadzu 2600 UV-visible spectrophotometer. Detailed chemical compositions were analyzed by X-ray photoelectron spectroscopy (XPS) on an ESCALAB 250Xi photoelectron spectrometer using monochromate Al Kα 150 W X-ray beam (1486.6 eV). All binding energies were referenced to the C 1 s peak (284.8 eV). The concentration of Pt was measured by inductively coupled plasma atomic emission spectroscopy (ICP-AES) from iCAP6300. The concentration of Cl was titrated by the ZDCL-2 chloride ion titrator (INESA). The electron paramagnetic resonance (EPR) spectra were collected on an Elexsys 580 spectrometer (ν = 9.86 GHz) with a center field at 3320 G and a sweep width of 140 G at room temperature. The X-ray absorption spectra (XAS) at the Pt L_3_-edge of the samples were recorded at room temperature in transmission mode using ion chambers (referenced samples) and fluorescence excitation mode using a Lytle detector (controlled samples) at beamline (BL) 14W1 of Shanghai Synchrotron Radiation Facility (SSRF).

### XAFS Measurement

The X-ray absorption spectra (XAS) at the Pt L_3_-edge of the samples were recorded at room temperature in transmission mode using ion chambers (referenced samples) and fluorescence excitation mode using Lytle detector and Solid-State-Detector (controlled samples) at beamline (BL) 14W1 of Shanghai Synchrotron Radiation Facility (SSRF). The station was operated with a Si (111) double crystal monochromator. During the measurement, the synchrotron was operated at 3.5 GeV and the current was between 150–210 mA. The data for each sample were calibrated with standard Pt metal foil. The XAS raw data were background subtracted, normalized, and Fourier transformed by standard procedures within the ATHENA program (version 0.9.26)^72^. The oxidation states and formal d-band hole counts of different single-atom Pt catalysts can be determined quantitatively by integrating the white-line intensity^24^. Least-squares curve fitting of the extended X-ray absorption fine structure spectra (EXAFS) χ(*k*) was carried out using the ARTEMIS program with the assistant of density functional theory (DFT), based on the EXAFS equation, which is expressed in terms of single- and multiple-scattering expansion:

1 $$\chi (k)={S}_{0}^{2}\mathop{\sum}\limits_{\varGamma }\frac{{N}_{\varGamma }|{F}_{{{{{{\rm{eff}}}}}}}^{\varGamma }(\pi,k,{R}_{\Gamma })|}{k{R}_{\varGamma }^{2}}\exp \left(-2{R}_{\varGamma }/\lambda (k)-2{k}^{2}{\sigma }_{\varGamma }^{2}\right)\\ \sin (2k{R}_{\varGamma }+{\phi }_{\varGamma }(k)+2{\delta }_{c}(k))$$

where *k* = [*m*_*e*_(*E-E*_*0*_)]^1/2^ represents a scale conversion from the photon energy (*E*, eV) to the wavenumber (*k*, Å^−1^) of the excited photoelectron, as measured from absorption threshold *E*_*0*_. The sums are over a series of equivalent scattering paths, *Γ*, which originates at the central absorption atoms, traveling to one or more of the neighboring atoms, and then back to the original central atoms. The equivalent scattering paths, with a degeneracy of *N*_*Γ*_, are grouped according to the atomic number of the passed atoms and the total path length *R*_*Γ*_ of the photoelectron. The dependence of the EXAFS oscillatory structure on path length and energy is reflected by the sin(2*kR*_*Γ*_ + *ϕ*_*Γ*_(*k*) + 2*δ*_*c*_(*k*)) term, where *ϕ*_*Γ*_(*k*) is the effective scattering phase shift for path *Γ*. *F*_*eff*_^*Γ*^(*π*, *k*, *R*_*Γ*_) denotes the effective scattering amplitude for path *Γ*. The amplitude decay due to inelastic scattering is captured by the exponential term *exp*(−2*R*_*Γ*_/*λ*(k)), where *λ*(k) is the photoelectron mean free path. The additional broadening effect due to thermal and structural disorder in absorber-scatterer(s) path lengths is accounted for by the Debye-Waller term *exp*(−2*k*^2^*σ*_*Γ*_^*2*^). *S*_0_^2^ is a many-body amplitude-reduction factor due to excitation in response to the creation of the core hole In this work, although the scattering amplitudes and phase shifts for all paths, as well as the photoelectron mean free path, were theoretically calculated by ab-initio code FEFF8.0^73^, the variable parameters that are determined by using the EXAFS equation to fit the experimental data are *N*_*Γ*_, *R*_*Γ*_, and *σ*_*Γ*_^*2*^. The *S*_0_^2^ parameter was determined in the fit of Pt foil standards and used as fixed value in the rest of the EXAFS models. All fits were performed in the *R* space with *k*-weight of 2. The EXAFS R-factor (R_f_), which measures the percentage misfit of the theory to the data, was used to evaluate the goodness of the fit^74^. The k^2^ weighting, k-range of 3-12.5 Å^−1^ and R range of 1-~3 Å were used for the fitting. The model of bulk Pt, K_2_PtCl_4_, PtO_2_ and DFT optimized structures were used to calculate the simulated scattering paths. For Wavelet Transform (WT) analysis, the χ(k) exported from Athena was imported into the Hama Fortran code^75,76^. The parameters were listed as follow: *R* range, 1–4 Å, *k* range, 0–12 Å^−1^; *k* weight, 2; and Morlet function with *κ* = 10, *σ* = 1 was used as the mother wavelet to provide the overall distribution^77–79^. Additional EXAFS simulations based on the best-fitted models were performed with FEFF8.0, and the thermal disorder was considered by using the correlated Debye model with Debye temperature of 475 K.

### CO chemisorption measurement via Fourier-transformed infrared (FT-IR) spectroscopy

The FT-IR spectra of CO chemisorption measurements were performed on the Nicolet 380 model (Thermo Electron Corporation) equipped with an MCT-A detector cooled by liquid nitrogen. The sample was first pre-reduced in the cell under 10% H_2_/Ar flow (20 mL min^−1^) at 100 °C for 1 h to remove any contaminant. After cooling the sample to room temperature under Ar flow (20 mL min^−1^), a background spectrum was collected. Then the sample was exposed to 10% CO/Ar (20 mL min^−1^) for 1 h until saturation. Subsequently, pure Ar (99.999%) was introduced at a flow rate of 20 mL min^−1^ for another 1 h to remove the gas-phase CO and then the FT-IR was collected with 64 scans at a resolution of 2 cm^−1^.

### In-situ Raman measurement

A Lab Ram ARAMIS Raman spectrometer (HORIBA) with a 633 nm stream-line laser excitation and the Cell with a quartz window (Shanghai chuxi industrial Co., LTD) were used for the in-situ Raman measurements. The obtained catalyst, Ag/AgCl and Pt wire were employed as the working, reference, and counter electrode, respectively. The laser beams were focused on the sample through the quartz window to collect Raman spectrum. The chronoamperometry method was employed to applied different voltages to the electrode with 25 mV intervals in the range of 0 to −0.1 V vs. RHE.

### Surface characterizations to gas bubbles

The gas-bubble contact angle with the volume of ≈0.5 μL was measured by the captive-bubble method and recorded using a microscope (SZX16, Olympus) mounted on a high-speed CCD camera (i-SPEED 3, AOS Technologies). The illumination was achieved by a fiber-optic illuminator system (CEL-TCX 250).

### CO stripping measurement

The CO stripping measurement was conducted in a three-electrode system in a H-type electrochemical cell. The graphic rod, silver/silver chloride electrode, and the obtained catalyst is used as counter electrode, reference electrode, and working electrode, respectively. Pure CO gas was first adsorbed on the working electrode at a fixed potential of 0.1 V vs. RHE in a CO-saturated 1 M KOH electrolyte for 10 min. All the CV of CO stripping were collected after purging with Ar gas at a scan rate of 20 mV s^−1^.

### Electrochemical Measurement for HER

All the electrochemical measurements were finished by a biologic potentiostat VMP3 electrochemical workstation with a three-electrode configuration at room temperature, in which the fabricated samples were performed as the working electrode, graphite rod acted as a counter electrode, and the mercury/mercury oxide (Hg/HgO) electrode acted as a reference electrode. For the powder-based samples, the glass carbon electrode coupled with a RDE was carried out as the working electrode with the rotating speed at 1600 rotation per minute (rpm). The electrolyte (1 M KOH solution) was purged with pure H_2_ for at least 30 min to obtain the H_2_ saturated solution before HER test. All potentials were corrected with IR compensation and then converted to RHE. The CV and linear sweep voltammetry (LSV) were performed at a scan rate of 50 mV s^−1^ and 10 mV s^−1^, respectively. For powder-based samples, the catalyst ink was prepared as following: 4 mg catalyst was ultrasonically dispersed in 1000 μL solution containing 480 μL water, 480 μL EtOH and 40 μL Nafion 117 ionomer solution. Electrochemical impedance spectroscopy (EIS) curves were obtained by a frequency range from 100 kHz to 1 Hz under different potential. The electrochemical active surface area (ECSA) was estimated based on ECSA = C_dl_ / C_s_, where C_dl_ corresponds to the double-layer charging current versus the scan rate and C_s_ corresponds to a specific capacitance. The linear fit slopes (C_dl_) for NiFe-LDH, Pt/C, Cl-Pt/LDH and HO-Pt/LDH are 2.94, 51.4, 9.62 and 6.88 mF cm^−2^, respectively. Compared with the specific capacitance (40 μF cm^−2^) of a smooth planar surface LDH with 1.0 cm^2^ surface area^80^, the ECSAs can be estimated as 73.5, 1285, 240.5 and 172 cm^2^_ECSA_ for NiFe-LDH, Pt/C, Cl-Pt/LDH and HO-Pt/LDH, respectively. The TOF of the catalysts was calculated according to the equation of TOF = I / (2 F × n), where I is the measured current during the LSV measurement, F is the Faraday constant of 96485 C mol^−1^, and n is the mole amount of Pt single atoms. The factor 1/2 represents two electrons are required to form one hydrogen molecule based on the equation of 2H^+^ + 2e^−^ → H_2_. The limiting current density ( *j*_*L*_) was calculated according to the Levich equation^60,81^: ${j}_{L}=0.62{nFA}{D}^{2/3}{\nu }^{-1/6}{c}_{0}{\omega }^{1/2}$, where *n* is the number of electrons transferred in HER (*n* = 2), *F* is the Faraday constant, *A* is the area of the electrode (0.196 cm^2^), *D* is the diffusion coefficient of H_2_ in 1 M KOH ( ≈ 3.03 × 10^5^ cm^2^ sec^−1^), *ν* is the kinematic viscosity of 1 M KOH electrolyte (≈0.998 × 10^−2^ cm^2^ s^−1^), *c*_*0*_ is the solubility of H_2_ in 1 M KOH electrolyte (≈0.5415 mM), and *ω* is the rotation rate^82,83^. The exchange current density ( *j*_*0*_) was conducted by a simple Bulter-Volmer equation fitting^11,17^: ${j}_{k}={j}_{0}\times ({e}^{\frac{\alpha F\eta }{{RT}}}-{e}^{\frac{\left(\alpha -1\right)F\eta }{{RT}}})$, where *j*_*k*_ is the measured current density normalized to the ECSA, *α* is the transfer coefficient, *F* is the Faraday constant, *η* is the overpotential, *R* is the ideal gas constant (8.314 J mol^−1^ K^−1^), and *T* is the temperature (298 K). Since the *j*_*L*_ is a constant under a certain test condition, reducing the catalyst loading amount is an effective way to reduce the impact of mass transport. At least three independent samples were employed to ensure the reproducibity of all the electrochemical measurements and other characterizations.The reaction product of H_2_ was measured using a gas chromatograph (GC-2014, SHIMADZU). Argon was used as the carrier gas in the chromatograph. The Faradaic efficiency (FE) was calculated based on FE = n_hydrogen_ / (Q_total_ / 2 F), where n_hydrogen_ is the produced mole of product H_2_, Q_total_ is the total electric quantity, and F is the Faradaic constant.

### Theoretical computation

Density functional theory (DFT) calculations were performed to investigate the geometric and electronic structures of Pt single-atom sites anchored on NiFe-LDH with different ligand and the mechanism for HER by using the Vienna Ab-initio Simulation package (VASP)^84–86^. The projected augmented wave (PAW) potential and generalized-gradient approximation (GGA) of the spin-polarized Perdew-Burke-Ernzerhof (PBE) functional were employed to describe the electron ion interaction and exchange correlation energy, respectively^87–89^. The energy cutoff for the plane-wave expansion was set at 400 eV for optimizing calculations of atoms and cell optimization. The Hubbard U (DFT+U) corrections^90,91^ for 3d transition metal were set using a U-J value 5.30 eV for Fe atom^92^ and 6.45 eV for Ni atom^93^. The DFT-D3 method was adopted to describe the van der Waals interactions between the adsorbed atoms and the support^65^. The construction of surface model was based on the single crystal of NiFe-LDH by constructing 2 × 2 × 1 supercell. The primitive cell model of NiFe-LDH was constructed with fully optimized atomic position, cell volume and cell shape. The vacuum spacing in a direction perpendicular to the plane of the catalyst was at 15 Å. Then, one Pt atom was directly placed on the top of one Fe atom and coordinated with three O atoms. After adding a X group (X = −F, −Cl, −Br, −I or −OH) at the top of Pt atom, the X-Pt/LDH model was constructed. The slab models were only optimized for the atomic positions. The equilibrium lattice constants were optimized with maximum stress on each atom within 0.02 eV Å^−1^ under quasi-Newton algorithm^94,95^. The self-consistent calculations applied a convergence energy threshold of 10^−5^ eV under the consideration of electronic relaxation to utilize conjugate-gradient method^96^. After geometry optimization, the lattice constants of Pt-SACs supercell with ~100 atoms are a = 12.35 Å, b = 12.27 Å, and c = 21.224 Å (Supplementary Fig. 38), which should be sufficient. Thus, the Brillouin zone integration was performed using 2 × 2 × 1 Monkhorst-Pack k-point sampling for all the Pt-SACs structures^97^.

The mechanism for alkaline hydrogen evolution reaction (HER) follows Volmer-Heyrovsky or Volmer-Tafel pathways (Volmer: H_2_O + M + e^−^ → M-H* + OH^−^; Heyrovsky: H_2_O + e^−^ +M-H* → H_2_ + OH^−^ + M; Tafel: 2M-H* → H_2_ + 2 M)^7^. To demonstrate mechanism for HER, we introduced the adsorption energies (E_ads_), defined as E_ads_ = E_(surface+adsorbate)_ - E_surface_ - E_adsorbate_, where the E_surface_ was the energy of different Pt SAC surface, E_adsorbate_ was the energy of the adsorbate, and the E_(surface+adsorbate)_ was the total energy of the surface with the adsorbate. According to the finite displacement method, the Gibbs free energy correction is necessary to obtain the accurate value for each system under a certain temperature and pressure by calculating the molecular vibration frequency^98^. Among them, the displacement on each degree of freedom is 2 times, and the convergence accuracy of the electronic step is 10^−7^ eV. All vibration frequencies lower than 50 cm^−1^ were set as 50 cm^−1^ due to the unreasonable contribution to the entropy from low frequency vibration^99^. The adsorption Gibbs free energy under standard atmospheric pressure (G_ads_) is defined as G_ads_ = E_ads_ + ∆G(T), where ∆G(T) was the sum of Gibbs free energy corrections at the temperature of T. Hence, we can conclude that ∆G(T) = ∆ZPE + ∆H(T) - T∆S(T), where ZPE, ∆H(T), and ∆S(T) represented by the zero-vibration energy change, the enthalpy change, and the entropy change before and after the adsorption. All the relevant thermodynamic quantities were calculated by vaspkit based on the above molecular vibration frequencies^100^. It was worth noticing that the correction for the solid surface was not included in the correction of free energy due to the correction value can be cancel out before and after the adsorption. Besides, for the adsorption of single atom A, all the energy before adsorption had been replaced by 1/n of elemental A_n_ energy.

The electronic structures of the catalyst were calculated by the density of state (DOS), where the calculation parameters were same with the geometric optimization mentioned above^101^. The average energy level *μ* for a specific orbital of the catalytic active site can be calculated by following:

2 $${{{{{\rm{\mu }}}}}}=\frac{{\int }_{{{{{{\rm{a}}}}}}}^{{{{{{\rm{b}}}}}}}{{{{{\rm{\varepsilon }}}}}}{{{{{\rm{N}}}}}}({{{{{\rm{\varepsilon }}}}}}){{{{{\rm{d}}}}}}{{{{{\rm{\varepsilon }}}}}}}{{\int }_{{{{{{\rm{a}}}}}}}^{{{{{{\rm{b}}}}}}}{{{{{\rm{N}}}}}}({{{{{\rm{\varepsilon }}}}}}){{{{{\rm{d}}}}}}{{{{{\rm{\varepsilon }}}}}}}$$

where ε was the energy level and N(ε) is the number of orbitals at the energy level ε. The parameter a and b were defined as the top or bottom limitation of the energy level for a certain orbital. The d-band center was identical with μ when a is −∞, b is zero and the orbital was d. Surface valence band photoemission spectra were corrected by subtracting the Shirley background^102^. The dynamic simulation was carried out to obtain the reaction energy barrier of the key primitive steps. Based on the structural optimization above, the related models were annealed to 298 K by the velocity adjustment method with a step value of 1 fs^103^. For every 25 steps, there was one heating rate adjustment, and the total adjustment number was 60. The restrictive molecular dynamic simulation was performed by using slow growth method with the maintain of the step length^104^. During the water dissociation process on Pt sites, the bond length of the broken O-H bond in H_2_O was taken as the limited freedom degree model with the increasement of 0.0005 Å fs^−1^.

### MEA measurement

The MEA setup with geometric area of 1 cm^2^ was purchased from gaossunion. The cathode and the anode were sandwiched with the membrane to form MEA, where the catalyst loading on both cathode and anode are 2 mg cm^−2^. 1 M of hot KOH solution was flowed through the MEA with a flow velocity of 15 mL min^−1^. The temperature of MEA was measured as 60 °C. The solution resistance for Cl-Pt/LDH / NiFe/LDH, Cl-Pt/LDH / Ir/C and Pt/C / Ir/C at open circuit voltage is 0.127 ohm, 0.237 ohm and 0.147 ohm, respectively. The energy efficiencies reported in this work are for the electrolysis cell only. The theoretical voltage (*E*_*0*_) of water splitting at 60 °C was calculated by *E*_*0*_ = −(ΔH−TΔS) / *n*F, where ΔH is the change in enthalpy during water splitting (−285.8 kJ mol^−1^), T is the reaction temperature, ΔS is the change in entropy during water splitting (−163.34 J K^−1^ mol^−1^), *n* is the number of electrons transferred (*n* = 2), and F is the Faradaic constant (96485 C mol^−1^). The energy efficiency of an electrolysis cell is defined as the net energy present in the hydrogen produced by the cell divided by the net energy consumed by the cell^105^. The net energy present in hydrogen is its higher heating value, which is 39.4 kWh/ kg of hydrogen.

## Supplementary information

Supplementary Information

## Data Availability

All the data generated or analyzed during this study have been included in the manuscript and Supplementary Information. All the data are also available upon reasonable request from the corresponding authors.
