# Supplementary material for: Pinpointing the axial ligand effect on platinum single-atom-catalyst towards efficient alkaline hydrogen evolution reaction
Source: Nat Commun. 2022 Nov 12;13:6875. doi: 10.1038/s41467-022-34619-5 (PMC9653394; doi:10.1038/s41467-022-34619-5)
Supplement: Supplementary file 1 — Supplementary Information [file 41467_2022_34619_MOESM1_ESM.pdf]

# Supplementary Information

## Pinpointing the axial ligand effect on platinum single-atom-catalyst towards efficient alkaline hydrogen evolution reaction

*Tianyu Zhang[1,2+], Jing Jin[1,3+], Junmei Chen[2], Yingyan Fang[1], Xu Han[1], Jiayi Chen[2], Yaping Li[1], Yu Wang[3], Junfeng Liu[1]* and Lei Wang[2]**

[1]State Key Laboratory of Chemical Resource Engineering, Beijing University of Chemical Technology, Beijing 100029, China

[2]Department of Chemical and Biomolecular Engineering, National University of Singapore, Singapore 117585, Singapore

[3]State Key Laboratory of Acoustics, Institute of Acoustics, Chinese Academy of Sciences Institution, Beijing 100190, China

[4]Shanghai Synchrotron Radiation Facility, Zhangjiang Laboratory, Shanghai Advanced Research Institute, Chinese Academy of Sciences, Shanghai 201204, China

E-mail: ljf@mail.buct.edu.cn; wanglei8@nus.edu.sg

[+] These authors contributed equally to this work.

# Table of contents

**Supplementary Figures** ........ **4**

Supplementary Fig. 1. Crystalline structure characterizations. ........ 4

Supplementary Fig. 2. Morphological characterizations of pristine NiFe-LDH. ........ 5

Supplementary Fig. 3. Digital photographs of the obtained samples. ........ 6

Supplementary Fig. 4. Elemental mappings of Cl-Pt/LDH and HO-Pt/LDH. ........ 7

Supplementary Fig. 5. Valence state characterizations. ........ 8

Supplementary Fig. 6. Oxidation state of Pt determined by XAS and FT-IR. ........ 9

Supplementary Fig. 7. Wavelet transform analysis. ........ 10

Supplementary Fig. 8. Formation energy calculation. ........ 11

Supplementary Fig. 9. Pt $L_3$-edge EXAFS analysis of Pt foil: ........ 12

Supplementary Fig. 10. Pt $L_3$-edge EXAFS analysis of $K_2PtCl_4$: ........ 13

Supplementary Fig. 11. Pt $L_3$-edge EXAFS analysis of $PtO_2$: ........ 14

Supplementary Fig. 12. Pt $L_3$-edge EXAFS analysis of Cl-Pt/LDH and HO-Pt/LDH: ........ 15

Supplementary Fig. 13. HER overpotentials and Faradaic efficiency. ........ 16

Supplementary Fig. 14. $SCN^-$ poison experiment. ........ 17

Supplementary Fig. 15. Electrodeposition of Pt on LDH. ........ 18

Supplementary Fig. 16. Combined HER and OER catalytic behavior. ........ 19

Supplementary Fig. 17. Highlighted Steady-state CV curves: ........ 20

Supplementary Fig. 18. ECSA measurements and ECSA-normalized current density. ........ 21

Supplementary Fig. 19. Tafel measurements of the obtained catalysts. ........ 22

Supplementary Fig. 20. Gas bubble adhesion behaviors. ........ 23

Supplementary Fig. 21. Tafel measurements of powder-based catalysts. ........ 24

Supplementary Fig. 22. Alkaline HER performance of powder-based catalysts ........ 25

Supplementary Fig. 23. Schematic diagram of equivalent circuit for HER mechanism. ........ 26

Supplementary Fig. 24. In-situ Cl ion titration measurement ........ 27

Supplementary Fig. 25. Morphology characterization of Cl-Pt/LDH after stability test. ........ 28

Supplementary Fig. 26. Valence state characterizations of Cl-Pt/LDH after stability test. ........ 29

Supplementary Fig. 27. Extended Stability test for Cl-Pt/LDH and Pt/C. ........ 30

Supplementary Fig. 28. Elemental mapping image of R-F-Pt/LDH. ........ 31

Supplementary Fig. 29. Elemental mapping image of R-Cl-Pt/LDH. ........ 32

Supplementary Fig. 30. Elemental mapping image of R-Br-Pt/LDH. ........ 33

Supplementary Fig. 31. Elemental mapping image of R-I-Pt/LDH. ........ 34

Supplementary Fig. 32. Valence state characterizations of reversed catalysts. ........ 35

Supplementary Fig. 33. HER performance of reversed catalysts. ............................................36
Supplementary Fig. 34. HER performance of powder-based reversed catalysts. .......................37
Supplementary Fig. 35. Bode plots for operando EIS measurements. ......................................38
Supplementary Fig. 36. Nyquist plots for operando EIS measurements. ..................................39
Supplementary Fig. 37. Correlation of the resistance to potential: ...........................................40
Supplementary Fig. 38. Computational models for the Pt-SACs. .............................................41
Supplementary Fig. 39. Computational models and localized electric field distribution. ..........42
Supplementary Fig. 40. Illustration of delocalized and localized d orbitals. ..............................43
Supplementary Fig. 41. CO stripping measurement for the control sample. ..............................44
Supplementary Fig. 42. CO stripping measurement for the obtained catalysts. .........................45
Supplementary Fig. 43. Impendance of MEA electrolysers. .....................................................46

**Supplementary Tables. ........................................................................................................47**

Supplementary Table 1. The contents of Pt loading in the obtained samples ............................47
Supplementary Table 2. EXAFS fitting parameters[a] at the Pt L-edge.......................................48
Supplementary Table 3. Comparison of alkaline HER activity. .................................................49
Supplementary Table 4. Optimum impedance fit parameters for Pt/C. ......................................50
Supplementary Table 5. Optimum impedance fit parameters for Cl-Pt/LDH. ...........................51
Supplementary Table 6. Optimum impedance fit parameters for HO-Pt/LDH. .........................52
Supplementary Table 7. Optimum impedance fit parameters for R-F-Pt/LDH. .........................53
Supplementary Table 8. Optimum impedance fit parameters for R-Br-Pt/LDH. .......................54
Supplementary Table 9. Optimum impedance fit parameters for R-I-Pt/LDH. ..........................55
Supplementary Table 10. Reaction energies[a] for the alkaline HER. ..........................................56

**Supplementary References ..................................................................................................57**

**Supplementary Figures**

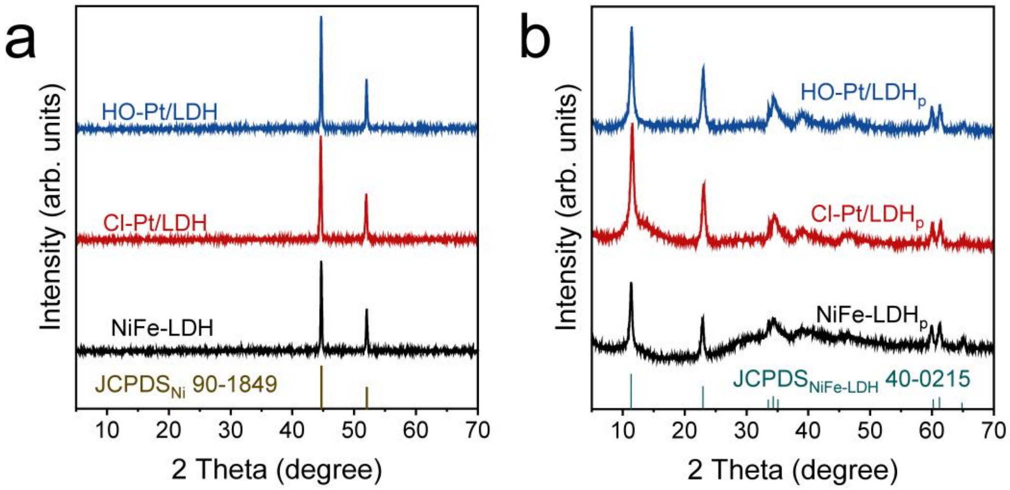


**Supplementary Fig. 1. Crystalline structure characterizations.** (a) XRD patterns of the as-prepared Cl-Pt/LDH, HO-Pt/LDH and NiFe-LDH. (b) XRD patterns of the powder-based Cl-Pt/LDH$_p$, HO-Pt/LDH$_p$ and NiFe-LDH$_p$. The characteristic peaks of Ni (JCPDS: 90-1849) at about 44° and 52° are too strong to overlap the appearance of diffraction peaks from the LDH array (Supplementary Fig. 1a). Thus, the powder-based samples are collected to substitute the loaded materials. As shown in Supplementary Fig. 1b, all the catalysts exhibit similar peaks in the XRD patterns, which can be ascribed to the typical NiFe-LDH structure (JCPDS: 40-0215). Besides, no characteristic peaks of Pt-based nanoparticles can be found in the XRD patterns.

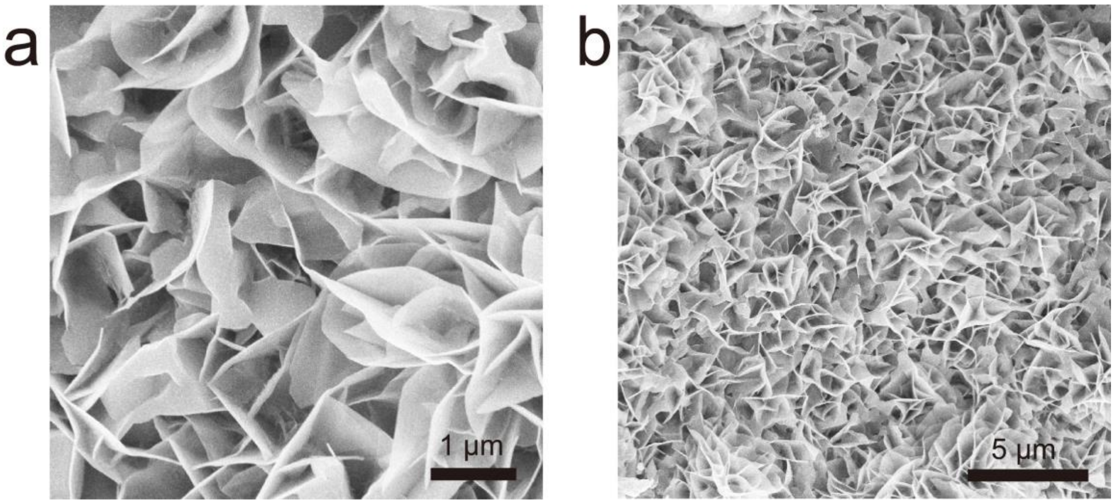


**Supplementary Fig. 2. Morphological characterizations of pristine NiFe-LDH.** (a) high and (b) low magnitude SEM images of NiFe-LDH.

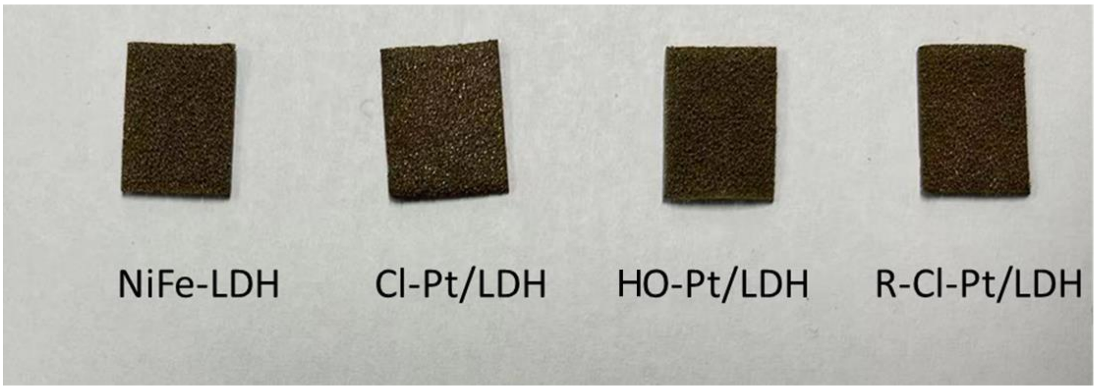


**Supplementary Fig. 3. Digital photographs of the obtained samples.**

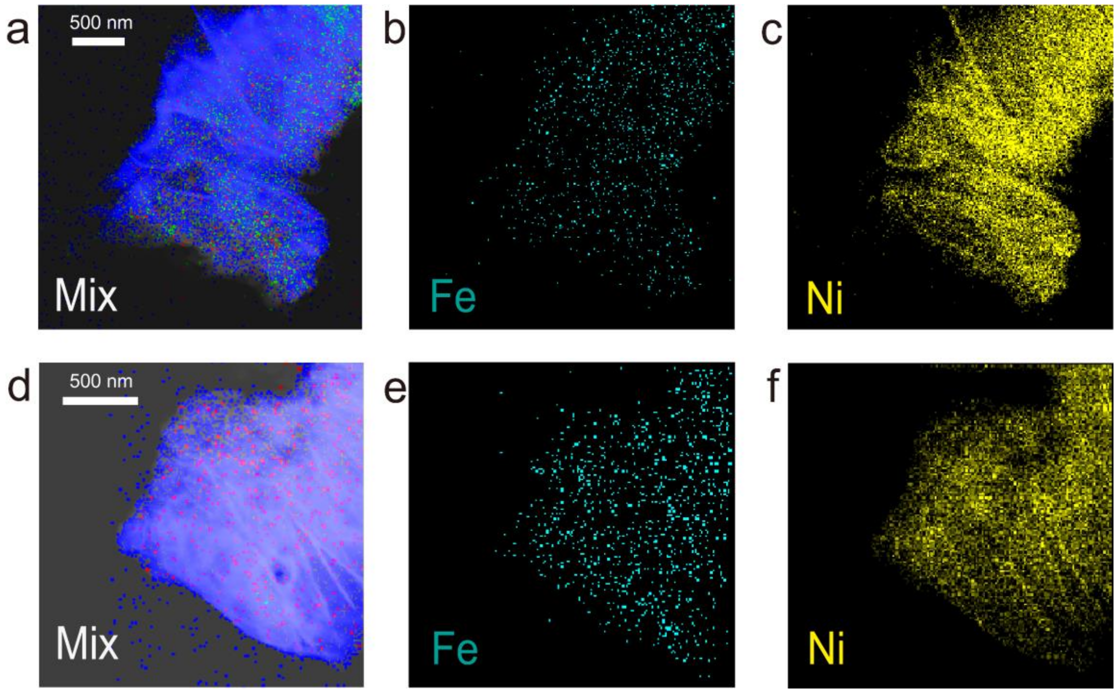


**Supplementary Fig. 4. Elemental mappings of Cl-Pt/LDH and HO-Pt/LDH.** (a, d) Mixed mapping for Pt (red), Cl (green) and O (blue); (b, e) Fe; and (c, f) Ni for Cl-Pt/LDH and HO-Pt/LDH, respectively.

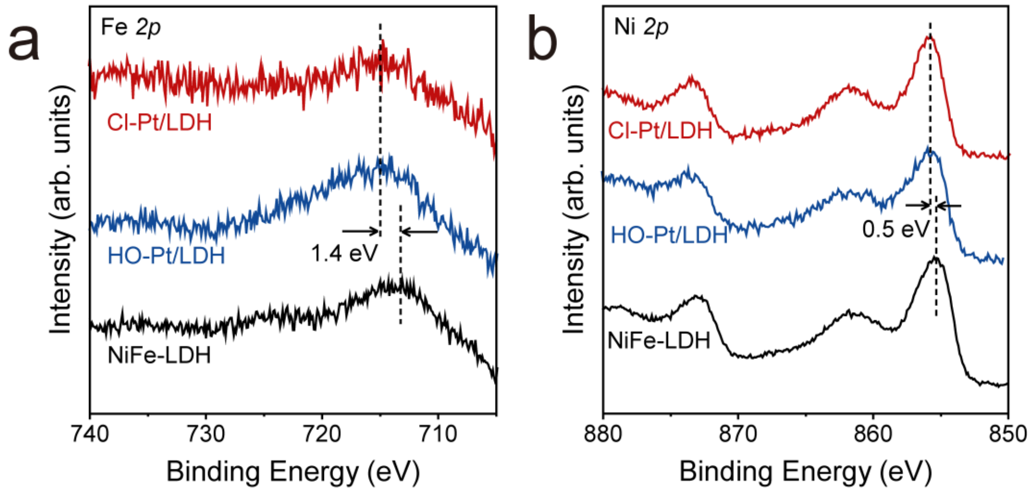


**Supplementary Fig. 5. Valence state characterizations.** High resolution (a) Fe *2p* and (b) Ni *2p* XPS spectra for Cl-Pt/NiFe-LDH, HO-Pt/NiFe-LDH and NiFe-LDH, respectively. The high-resolution Fe *2p* and Ni *2p* XPS spectra exhibit an increase of oxidation state during the electroreduction process, demonstrating the successful Pt loading with significant charge transfer between the Pt atoms and NiFe-LDH supports.

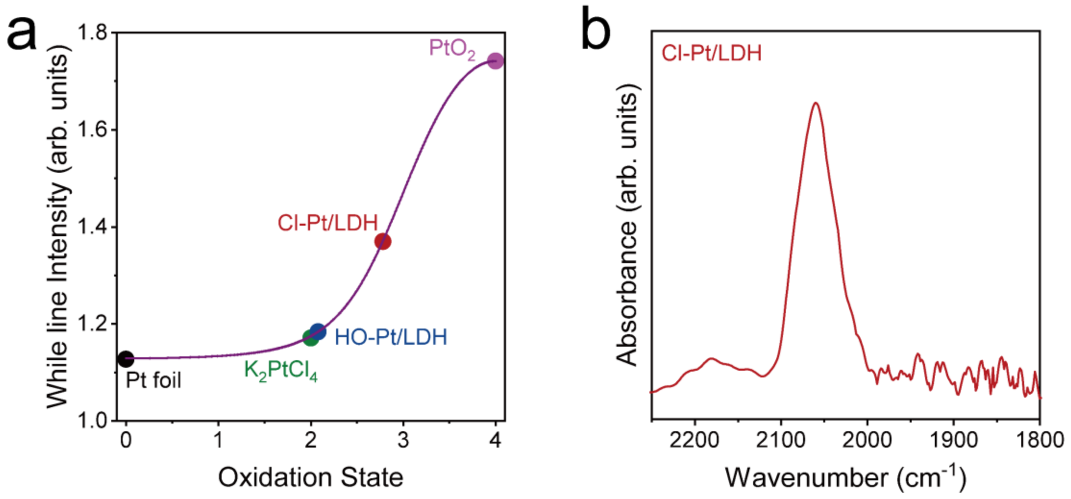


**Supplementary Fig. 6. Oxidation state of Pt determined by XAS and FT-IR.** (a). White line intensity of the Pt $L_3$-edge in as-prepared samples and reference samples vs. Pt oxidation state. Typically, the relationship between the oxidation state of Pt and the white line intensity is considered as linearity by only using Pt foil and $PtO_2$ as the reference[1]. However, after introducing $K_2PtCl_4$ as a third reference, the linear relationship is not satisfied. The Levenberg-Marquardt algorithm with a R-square of 0.9988 is selected by trial-and-error strategy to speculate the oxidation state of Pt in unknown samples[2, 3]. (b). FT-IR of CO chemisorption on Cl-Pt/LDH at CO saturation coverage. The adsorption of CO leads to a strong vibration band at ~ 2050 $cm^{-1}$, which can be ascribed to the linearly bonded CO on single Pt sites. The absence of CO bridged adsorption peaks on two neighboring Pt atoms at ~1860 $cm^{-1}$ and 1950 $cm^{-1}$ provides strong evidence for the atomic dispersion of Pt in our samples[4].

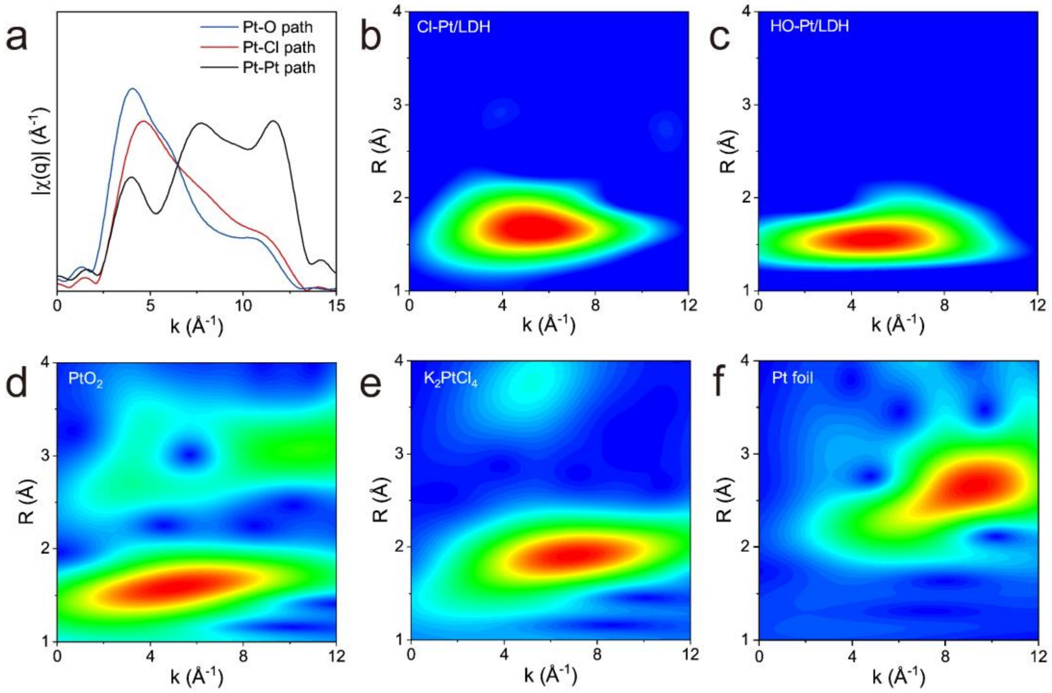


**Supplementary Fig. 7. Wavelet transform analysis.** Wavelet transform (WT) analysis of the EXAFS spectra is effective in identifying the overlapped contributions originated from different neighbor atoms or scattering events (single or multiple scattering) in both K- and R-spaces[5]. (a) Plots of the magnitude χ(q), calculated for the single scattering paths involving the Pt absorber and the first shell coordination numbers, including O, Cl and Pt. Wavelet transform of the $k^2$-weighted Pt $L_3$-edge EXAFS signals of (b) Cl-Pt/LDH, (c) HO-Pt/LDH, (d) $PtO_2$, (e) $K_2PtCl_4$, and (f) Pt foil, based on Morlet wavelets with optimum resolutions at 2.0 Å. Moreover, the Pt-sites in these samples are once again confirmed to be mononuclear centre due to the absence of WT signal at high k value over ~10.0 $Å^{-1}$.

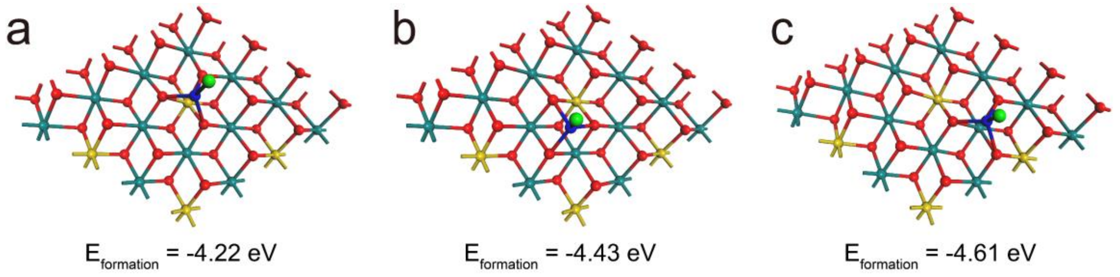


**Supplementary Fig. 8. Formation energy calculation.** The models with Pt atoms on the (a) Fe (b) O, and (c) Ni sites of the NiFe-LDH are considered and calculated by the DFT method. The blue, olive, yellow, red, and green balls refer to Pt, Ni, Fe, O, and Cl atoms, respectively. The results showed that the Pt atoms located on the top of Fe sites were most stable in the above three models.

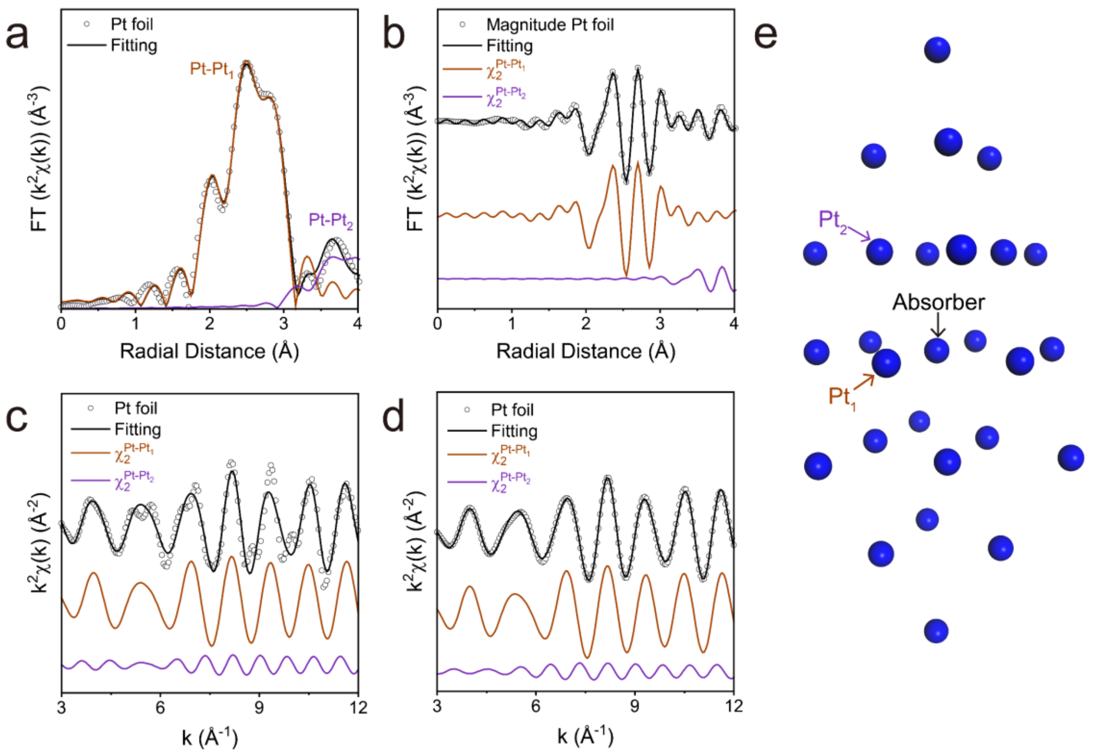


**Supplementary Fig. 9. Pt L3-edge EXAFS analysis of Pt foil:** (a) R space, (b) real R space, (c) k space and (d) q space, respectively. The data are $k^2$-weighted and not phase-corrected. (e) The schematic model of Pt foil: Pt (blue).

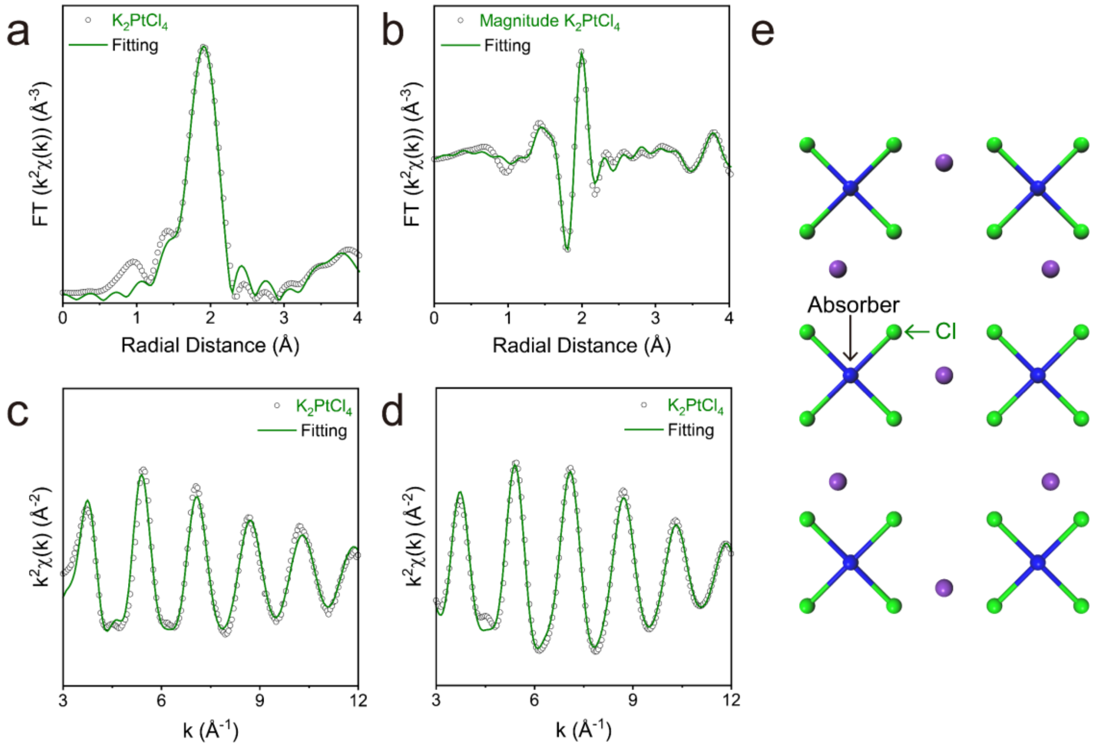


**Supplementary Fig. 10. Pt $L_3$-edge EXAFS analysis of $K_2PtCl_4$:** (a) R space, (b) real R space, (c) k space and (d) q space, respectively. The data are $k^2$-weighted and not phase-corrected. (e) The schematic model of Pt foil: Pt (blue), Cl (green), K (purple).

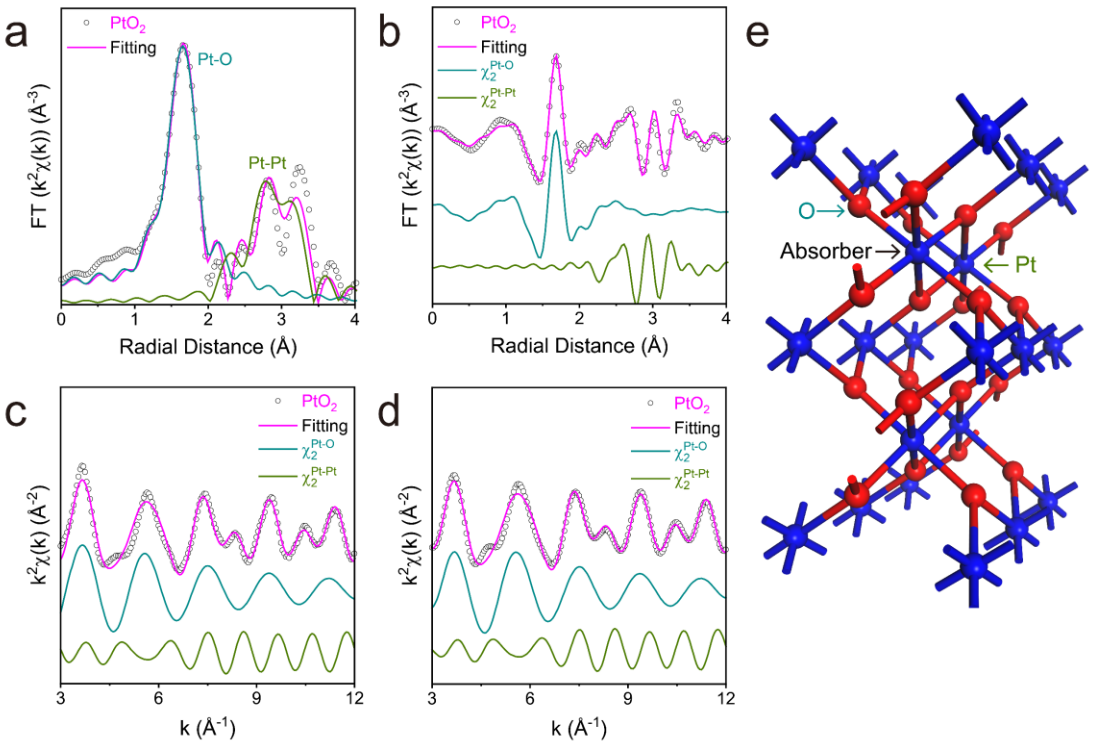


**Supplementary Fig. 11. Pt $L_3$-edge EXAFS analysis of $PtO_2$:** (a) R space, (b) real R space, (c) k space and (d) q space, respectively. The data are $k^2$-weighted and not phase-corrected. (e) The schematic model of Pt foil: Pt (blue), O (red).

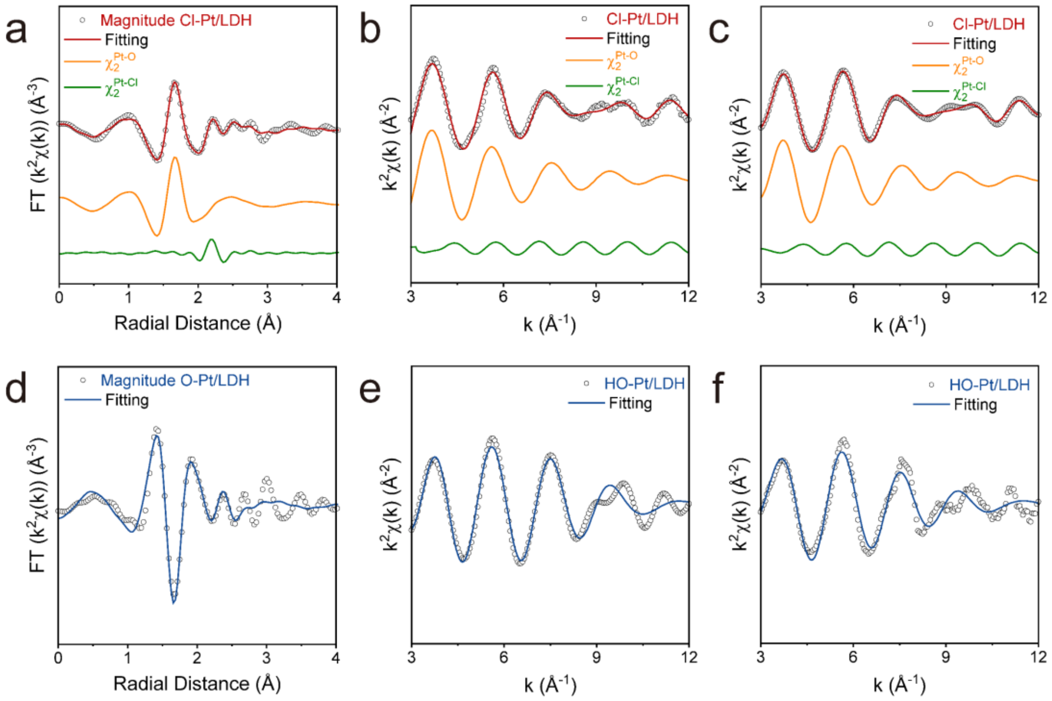


**Supplementary Fig. 12. Pt $L_3$-edge EXAFS analysis of Cl-Pt/LDH and HO-Pt/LDH:** (a, d) real R space, (b, e) k space and (c, f) q space, respectively. The data are $k^2$-weighted and not phase-corrected.

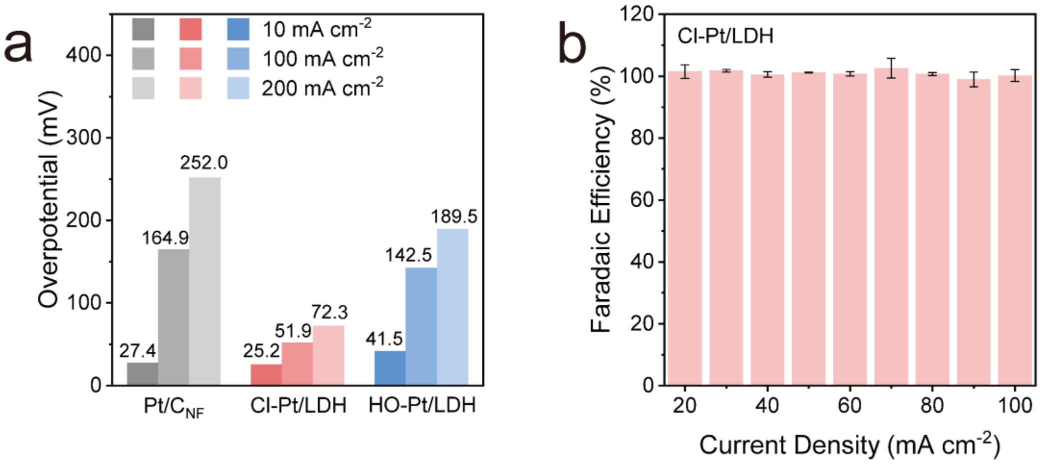


**Supplementary Fig. 13. HER overpotentials and Faradaic efficiency.** (a) Overpotentials required to achieve current densities of 10, 100, and 200 mA $cm^{-2}$ for Pt/$C_{NF}$, Cl-Pt/LDH, and HO-Pt/LDH. (b) Faradaic efficiency of Cl-Pt/LDH under different current density.

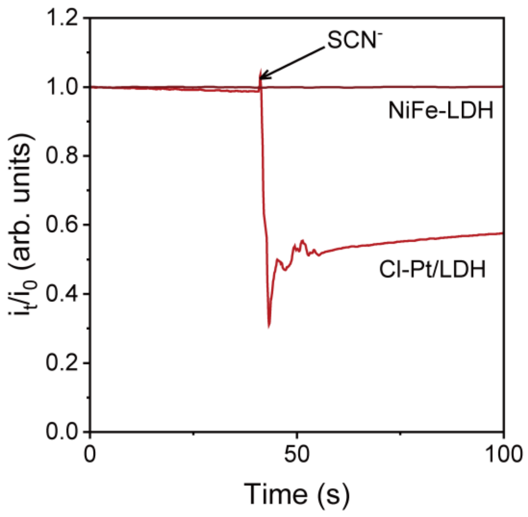


**Supplementary Fig. 14. $SCN^-$ poison experiment.** Current density-time curves of Cl-Pt/LDH and pristine NiFe-LDH before and after the addition of 50 mM thiocyanate ions ($SCN^-$) at -100 mV vs. RHE in 1M KOH.

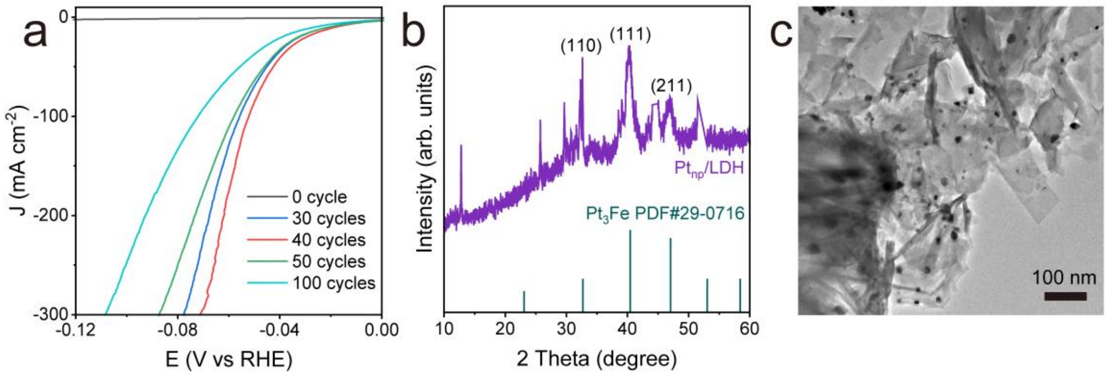


**Supplementary Fig. 15. Electrodeposition of Pt on LDH.** (a) LSV curves for electrodeposition of Pt with different CV cycles. (b) XRD pattern and (c) TEM image of $Pt_{np}$/LDH. The sample with 40 cycles is considered to possess maximum atomic Pt loading amount. The deficient performance of the catalysts with smaller CV cycles indicates the loading amount of Pt is not enough. On the contrary, further increasing the Pt loading will lead to the Pt nanoparticles formation and activity degradation. It is worth noticing that the nanoparticles exhibit XRD characteristic peaks of $Pt_3Fe$ alloy (JCPDS: 29-0716), indicating that Pt is close to Fe in space, which consistent well with our DFT calculation in Supplementary Fig. 8.

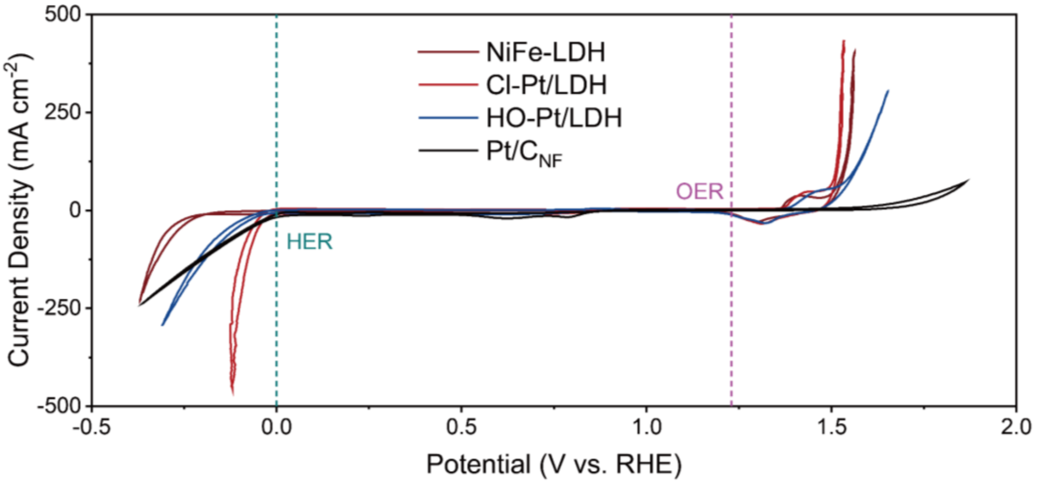


**Supplementary Fig. 16. Combined HER and OER catalytic behavior.** Steady-state CV curves at a scan rate of 100 mV $s^{-1}$ Cl-Pt/LDH, HO-Pt/LDH, NiFe-LDH and Pt/$C_{NF}$.

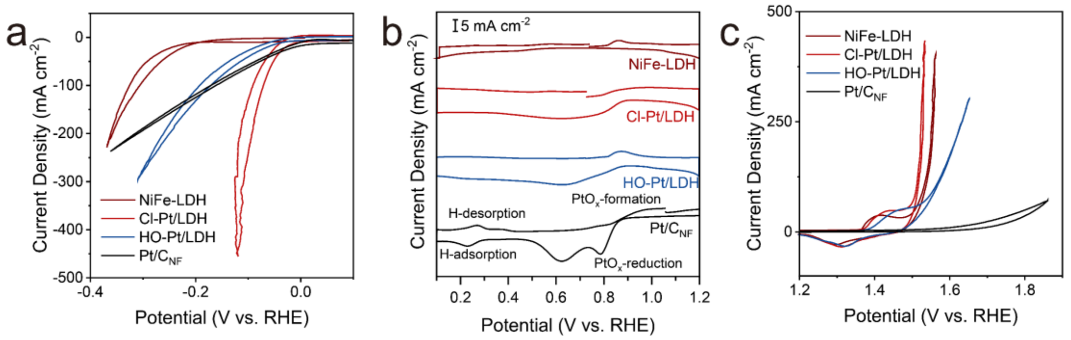


**Supplementary Fig. 17. Highlighted Steady-state CV curves:** (a) HER, (b) pre-reaction, and (c) OER region. The hydrogen desorption peaks for Pt/$C_{NF}$ at 0.28 V in the anodic sweep showed the presence of (110) facets[6]. Besides, the characteristic of redox peaks of NiFe-LDH can be observed for NiFe-LDH, Cl-Pt/LDH and HO-Pt/LDH in the OER region.

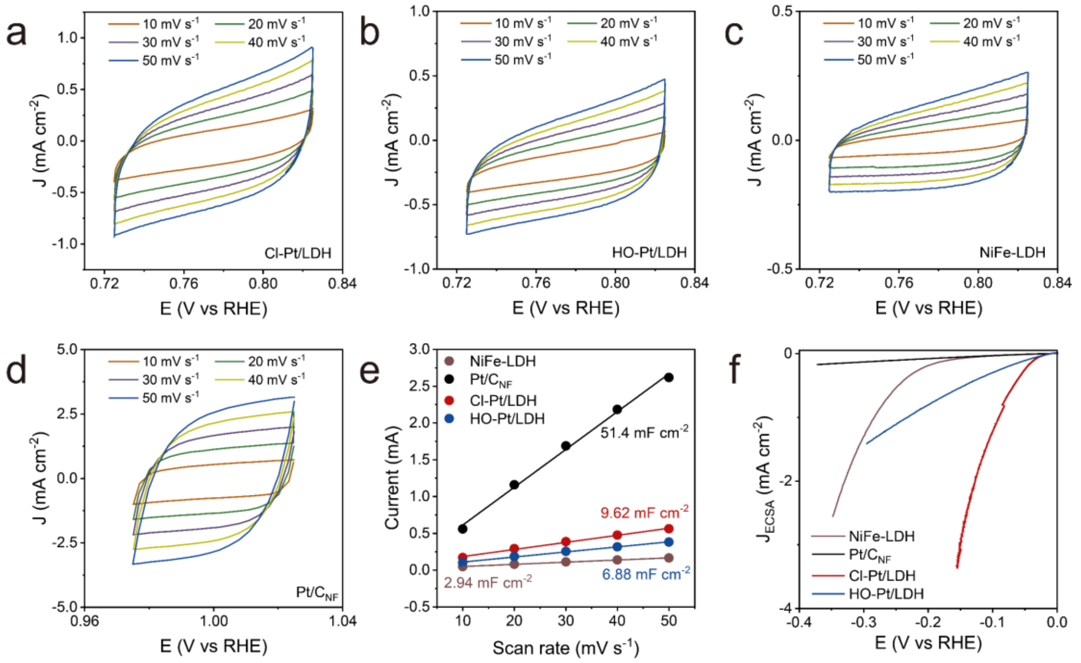


**Supplementary Fig. 18. ECSA measurements and ECSA-normalized current density.** CV curves at non-Faradaic region with various scan rates for (a) Cl-Pt/LDH, (b) HO-Pt/LDH, (c) NiFe-LDH, and (d) Pt/$C_{NF}$. The non-faradaic area was selected from the steady-state CV curves. (e) Electrochemical double-layer capacity and (f) ECSA normalized HER polarization curves of Cl-Pt/LDH, HO-Pt/LDH, NiFe-LDH and Pt/$C_{NF}$, respectively.

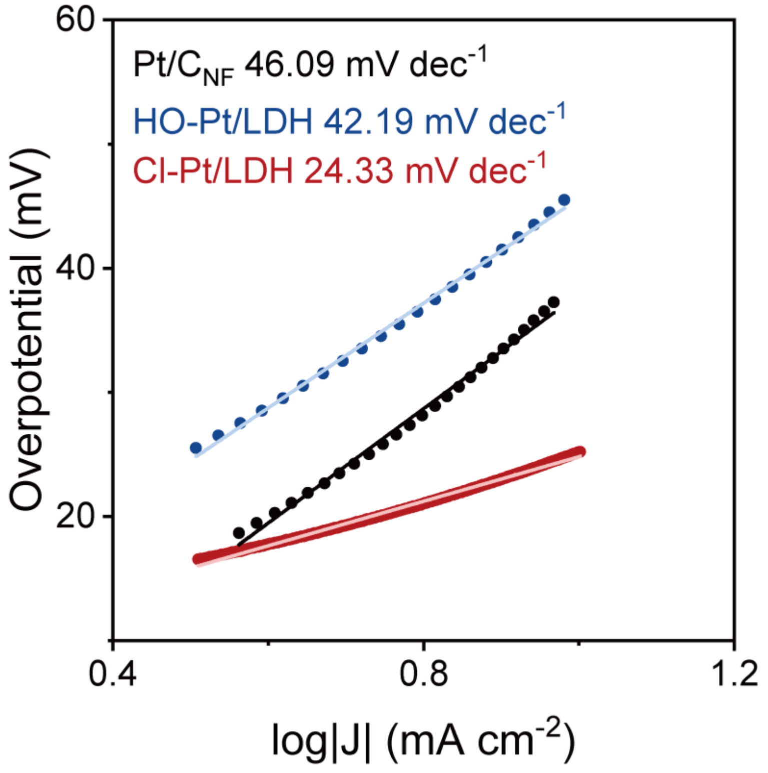


**Supplementary Fig. 19. Tafel measurements of the obtained catalysts.** Tafel slope analysis originated from LSV curves in Fig. 3a under the mass transport limitation.

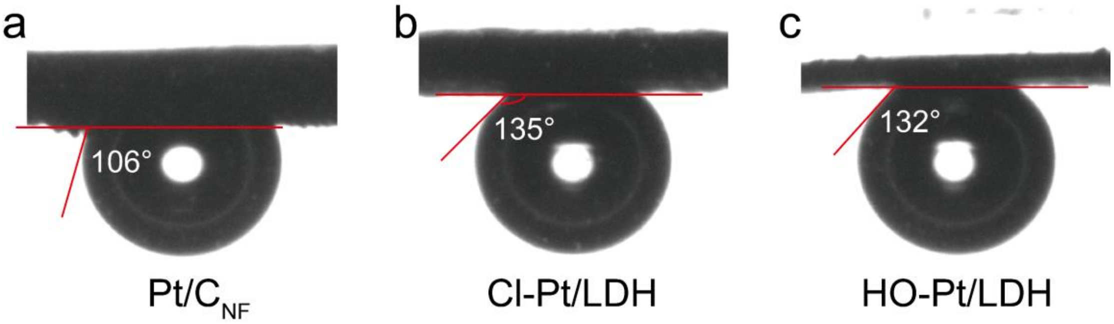


**Supplementary Fig. 20. Gas bubble adhesion behaviors.** The optical picture and corresponding contact angle for (a) Pt/$C_{NF}$, (b) Cl-Pt/LDH, and (c) HO-Pt/LDH, respectively. The Tafel difference originated from the mass transport limitation under similar hydrogen partial pressure were attributed to the aerophilic induced by the unique nanoarray structural advantages[7].

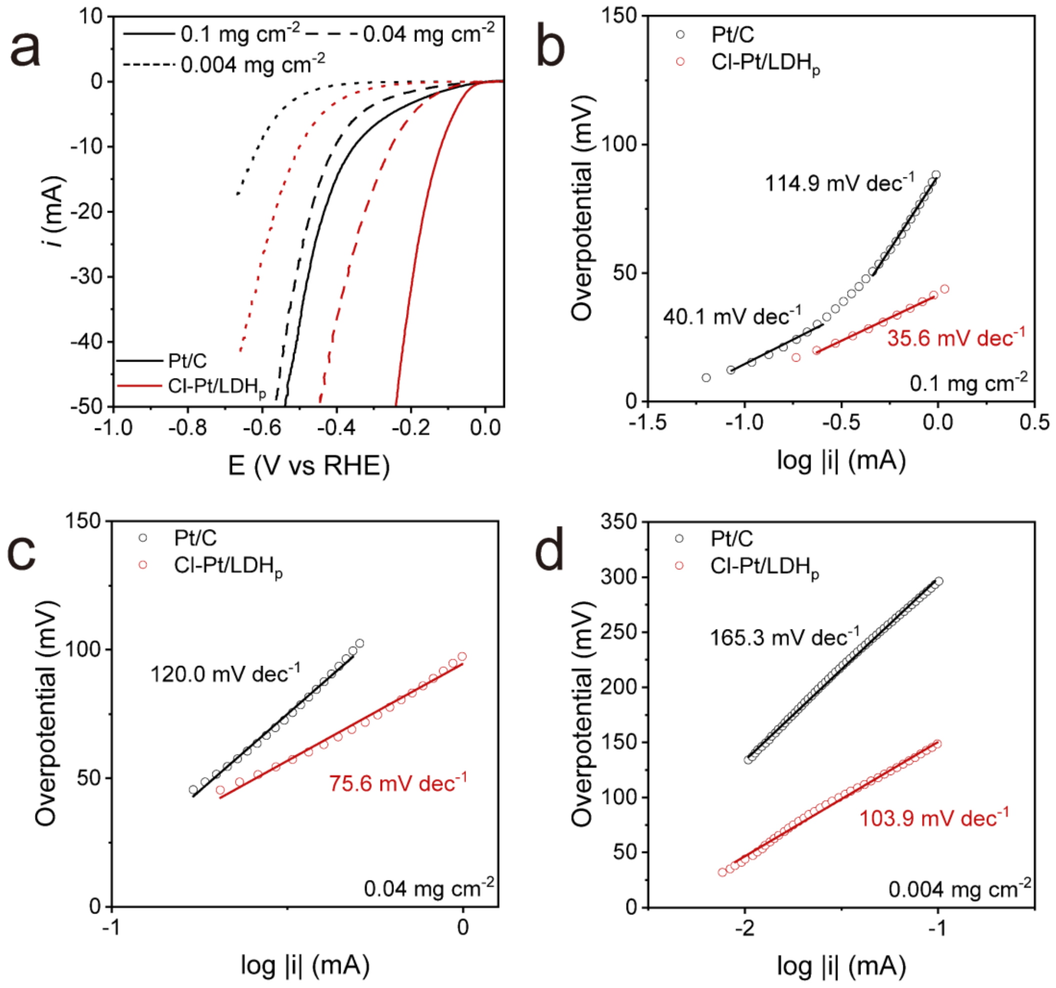


**Supplementary Fig. 21. Tafel measurements of powder-based catalysts.** (a) HER polarization curves of the powder-based Cl-Pt/$LDH_p$ and Pt/C with different loading amount on RDE. Corresponding Tafel slope analysis with the loading amount of (b) 0.1 mg cm$^{-2}$, (c) 0.04 mg cm$^{-2}$, and (d) 0.004 mg cm$^{-2}$. Note, with the loading amount of 0.004 mg cm$^{-2}$, the catalyst was difficult to spread evenly across the electrode, thus the catalysts loading amount of 0.04 mg cm$^{-2}$ was selected to exclude the $H_2$ mass transportation limitation and further investigate the ligand effect on Pt single-sites. Note, these factors including reactants diffusion, hydrogen mass transportation and solution resistance are difficult to be eliminated. Thus, the intrinsic fast HER kinetics are usually underestimated due to experimental limitations [8].

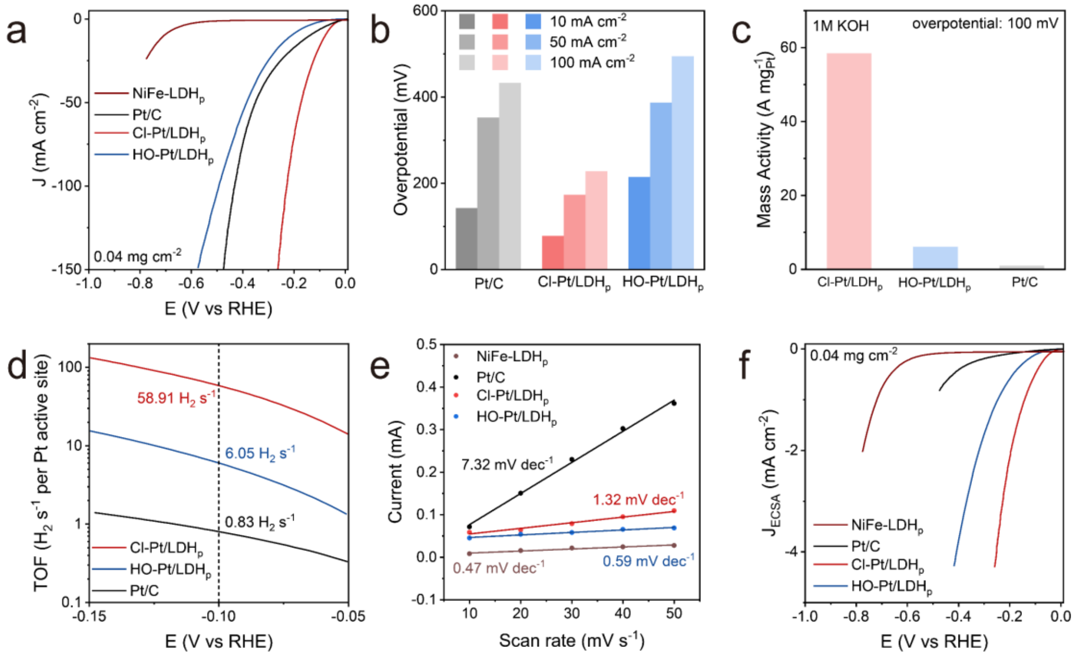


**Supplementary Fig. 22. Alkaline HER performance of powder-based catalysts.** (a) HER polarization curves of the powder-based NiFe-$LDH_p$, Cl-Pt/$LDH_p$, HO-Pt/$LDH_p$ and Pt/C on RDE. (b) Overpotentials required to achieve current densities of 10, 50, and 100 mA $cm^{-2}$ for various of catalysts. (c) The mass activity and (d) TOFs plots of the Pt-based catalysts. (e) Electrochemical double-layer capacity and (f) ECSA normalized HER polarization curves of Cl-Pt/$LDH_p$, HO-Pt/$LDH_p$, NiFe-$LDH_p$ and Pt/C, respectively. Note, after excluding the $H_2$ mass transport effect, the catalyst with better performance was even more underrated.

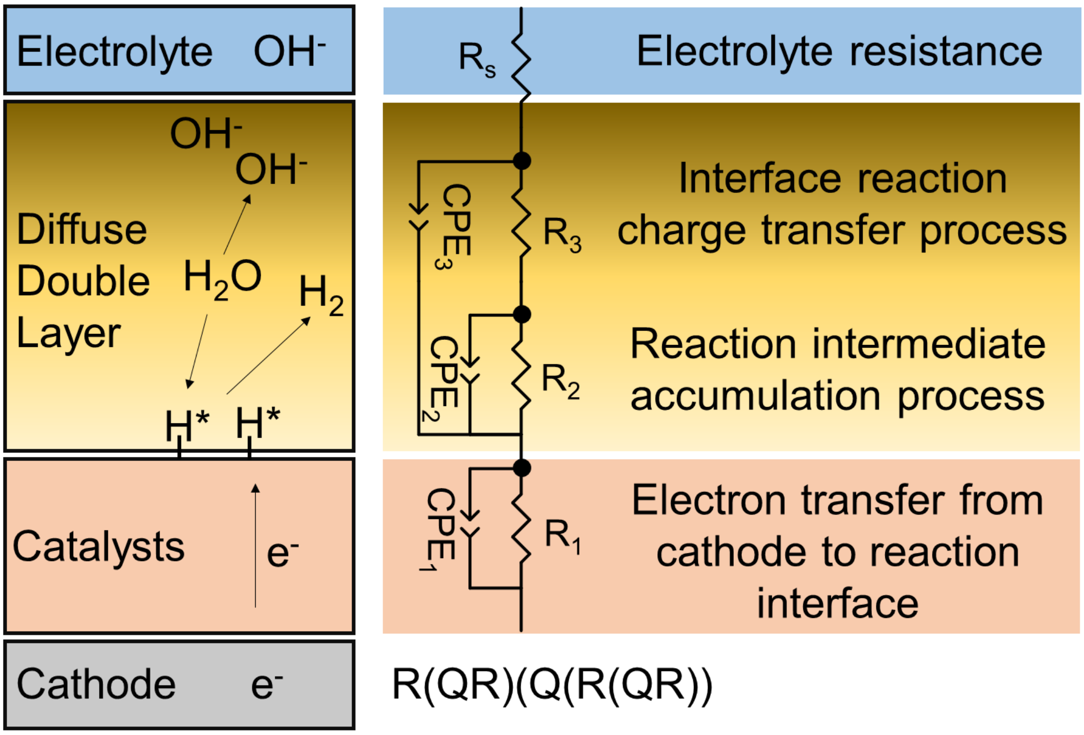


**Supplementary Fig. 23. Schematic diagram of equivalent circuit for HER mechanism.** Resistance (R) and constant phase element (CPE) are used to construct the equivalent circuit. Typically, the equivalent circuit consists of four parts: electron transfer from the cathode to the reaction interface ($R_1$ and $CPE_1$); reaction intermediate (H*) accumulation ($R_2$ and $CPE_2$); interface reaction charge transfer ($R_3$ and $CPE_4$); and electrolyte resistance ($R_s$). The mechanism of HER can be subdivided into Volmer ($H_2O + M + e^- \rightarrow M\text{-}H^* + OH^-$), Heyrovsky ($H_2O + M\text{-}H^* + e^- \rightarrow M + H_2 + OH^-$), and Tafel ($2M\text{-}H^* \rightarrow 2M + H_2$) steps. Based on Tafel slope, $b = (2.304RT) / [(\alpha^*e + n)F]$, where $\alpha^*$ is the number of electron transfer for RDS and n is the number of electron transfer before RDS), the alkaline HER mechanism can be determined as Volmer-Tafel (~30 mV $dec^{-1}$) or Volmer-Heyrovsky (~40 mV $dec^{-1}$). For Volmer-Tafel mechanism ($\alpha^* = 0$ and $n = 2$), electron transfer occurs only in the Volmer step, and Tafel step without any electron transfer cannot identified by EIS. Thus, $CPE_1$ and $R_1$, $CPE_2$ and $R_2$ in parallel of the equivalent circuit represent the electron transferability and Volmer step, respectively. For Volmer-Heyrovsky mechanism ($\alpha^* = n = 1$), two electrons occur in Volmer and Heyrovsky step, respectively. Thus, $CPE_1$ and $R_1$, $CPE_2$ and $R_2$, and $CPE_3$ and $R_3$ in parallel of the equivalent circuit represent the electron transferability, Volmer step and Heyrovsky step, respectively. Owing to excellent electron transfer ability of the obtained electrocatalytic catalysts, $CPE_1$ and $R_1$ can be ignored[9-11].

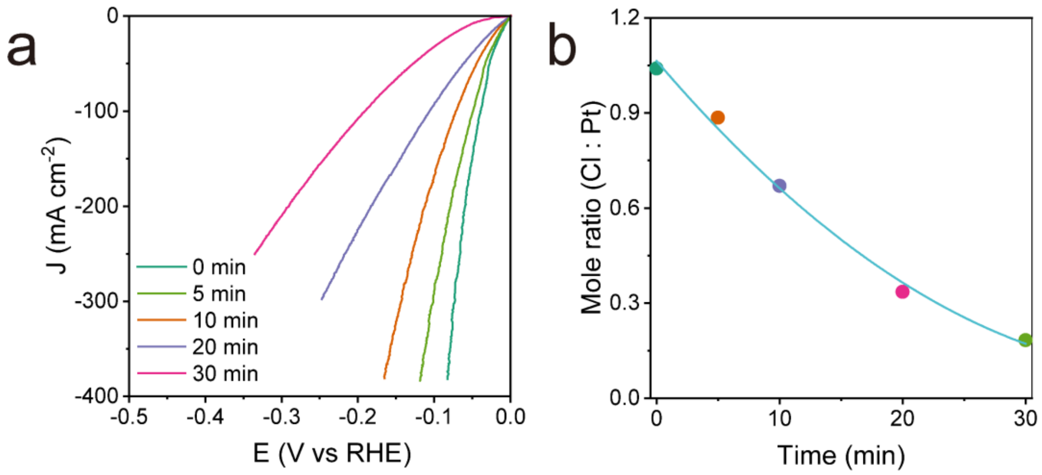


**Supplementary Fig. 24. In-situ Cl ion titration measurement.** (a) LSV curves for Cl-Pt/LDH with different irradiation time. (b) The Cl : Pt mole ratio change versus irradiation time.

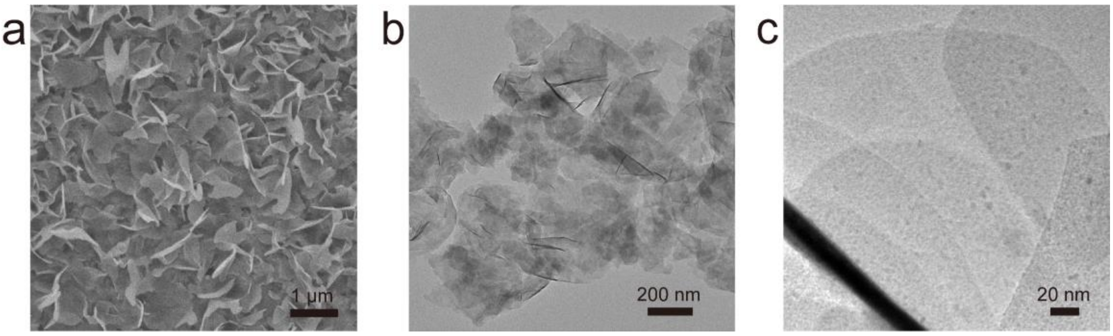

**Supplementary Fig. 25. Morphology characterization of Cl-Pt/LDH after stability test.** (a) SEM, (b) TEM, and (c) HRTEM image of Cl-Pt/LDH after stability test.

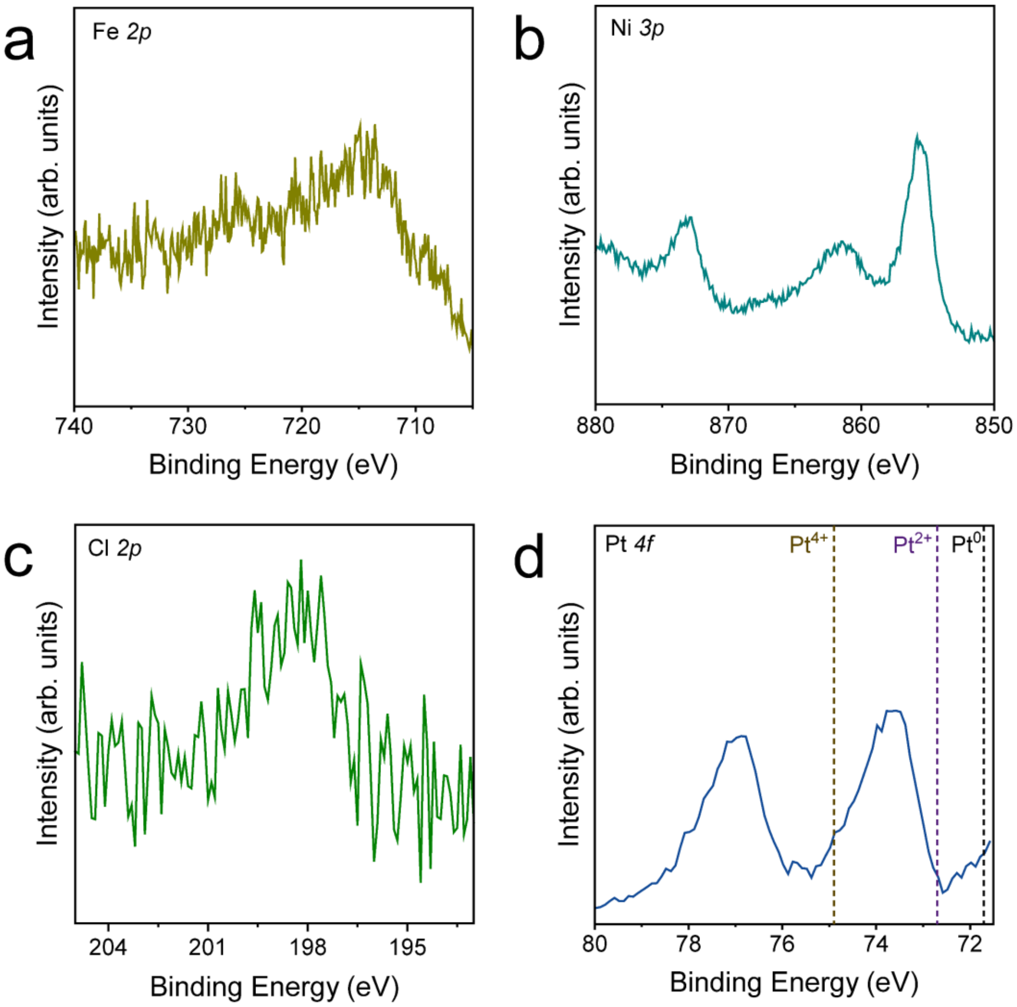


**Supplementary Fig. 26. Valence state characterizations of Cl-Pt/LDH after stability test.** High resolution (a) Fe *2p*, (b) Ni *2p*, (c) Cl *2p*, and (d) Pt *4f* XPS spectra for Cl-Pt/NiFe-LDH after stability test, respectively.

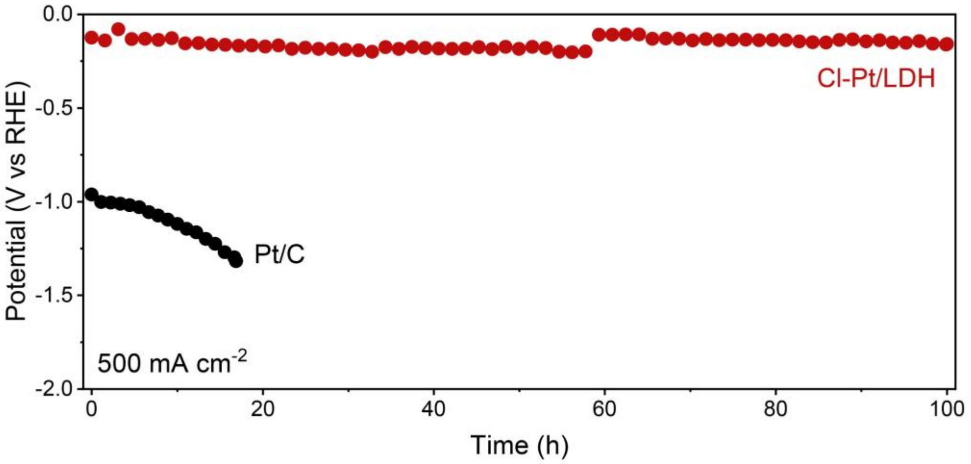


**Supplementary Fig. 27. Extended Stability test for Cl-Pt/LDH and Pt/C.** Plot of potential vs time for the Cl-Pt/LDH and commercial Pt/C at a constant cathodic current density of 500 mA cm$^{-2}$. The unuaully large overpotential of Pt/C is likely due to the inefficient mass transport of $H_2$ bubbles from the electrode surface (refer to the gas bubble adhesion measurement, Supplementary Fig. 20). This issue is less sever in the MEA configuration ($H_2$ gas escape from the back of the electrode), as evidenced by the data form Fig. 5 in the main manuscript. Nontheless, this test demostrate the satisfactory robustness of Cl-Pt/LDH under the alkaline HER conditions.

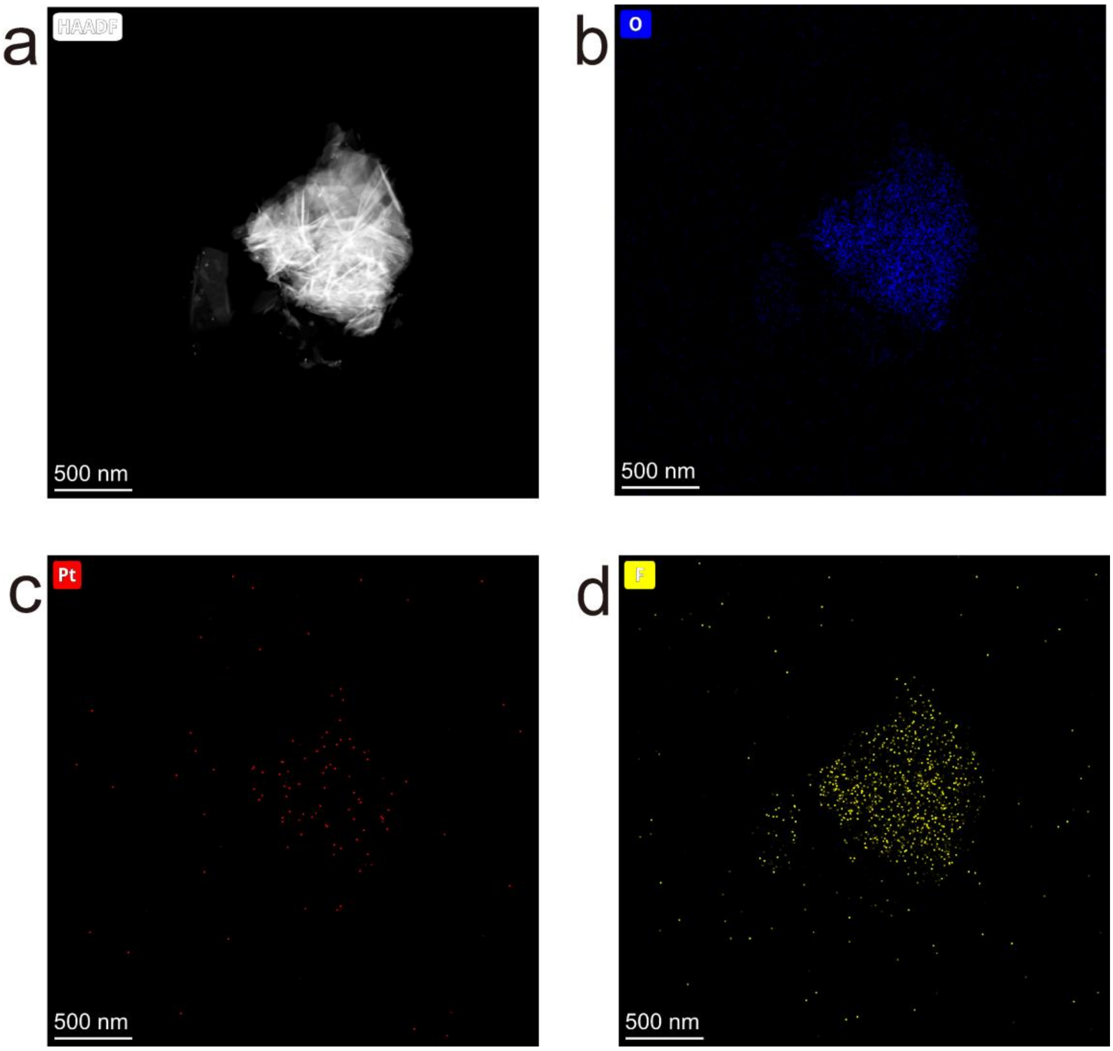


**Supplementary Fig. 28. Elemental mapping image of R-F-Pt/LDH.** (a) HAADF-STEM image and corresponding elemental mapping image of (b) O, (c) Pt, and (d) F.

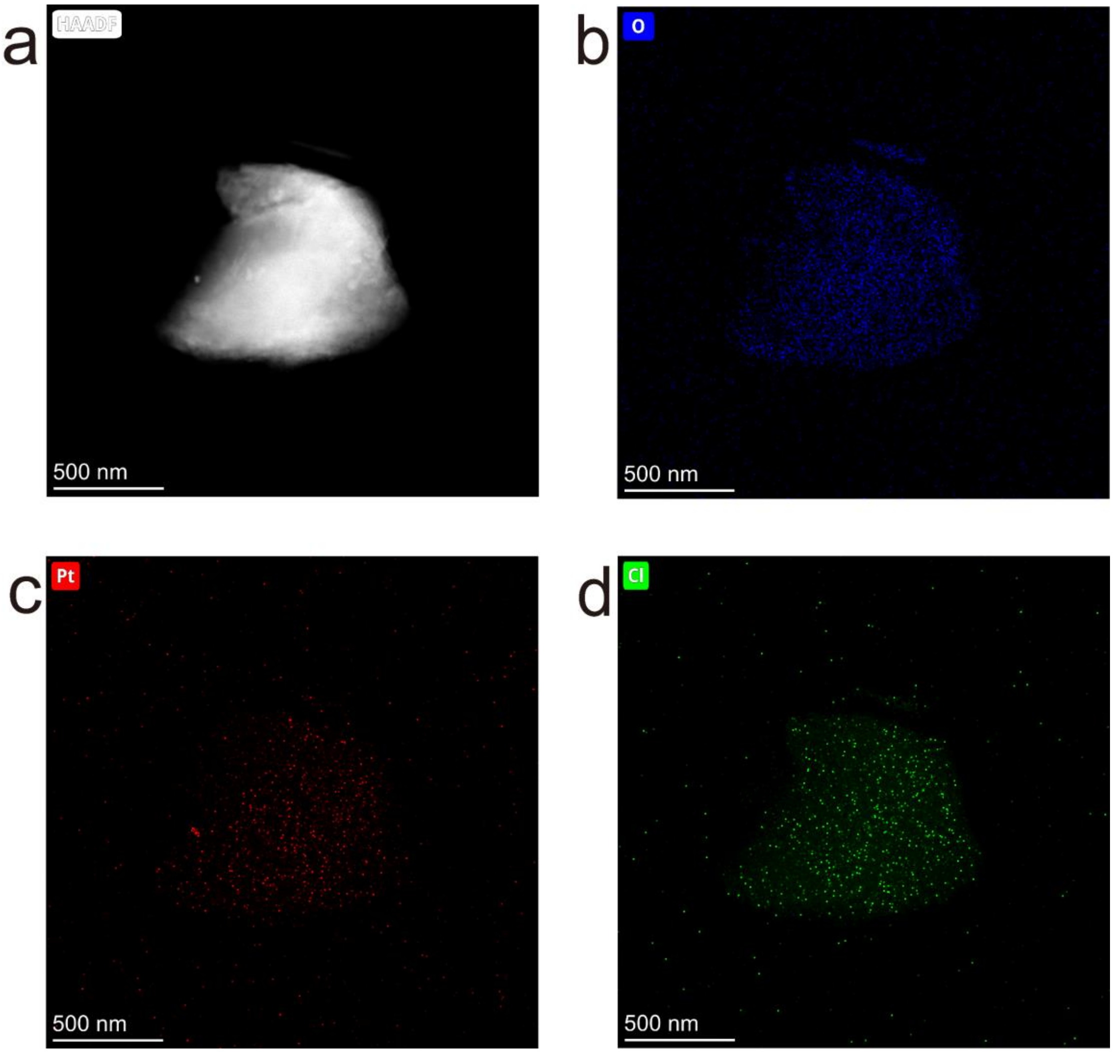


**Supplementary Fig. 29. Elemental mapping image of R-Cl-Pt/LDH.** (a) HAADF-STEM image and corresponding elemental mapping image of (b) O, (c) Pt, and (d) Cl.

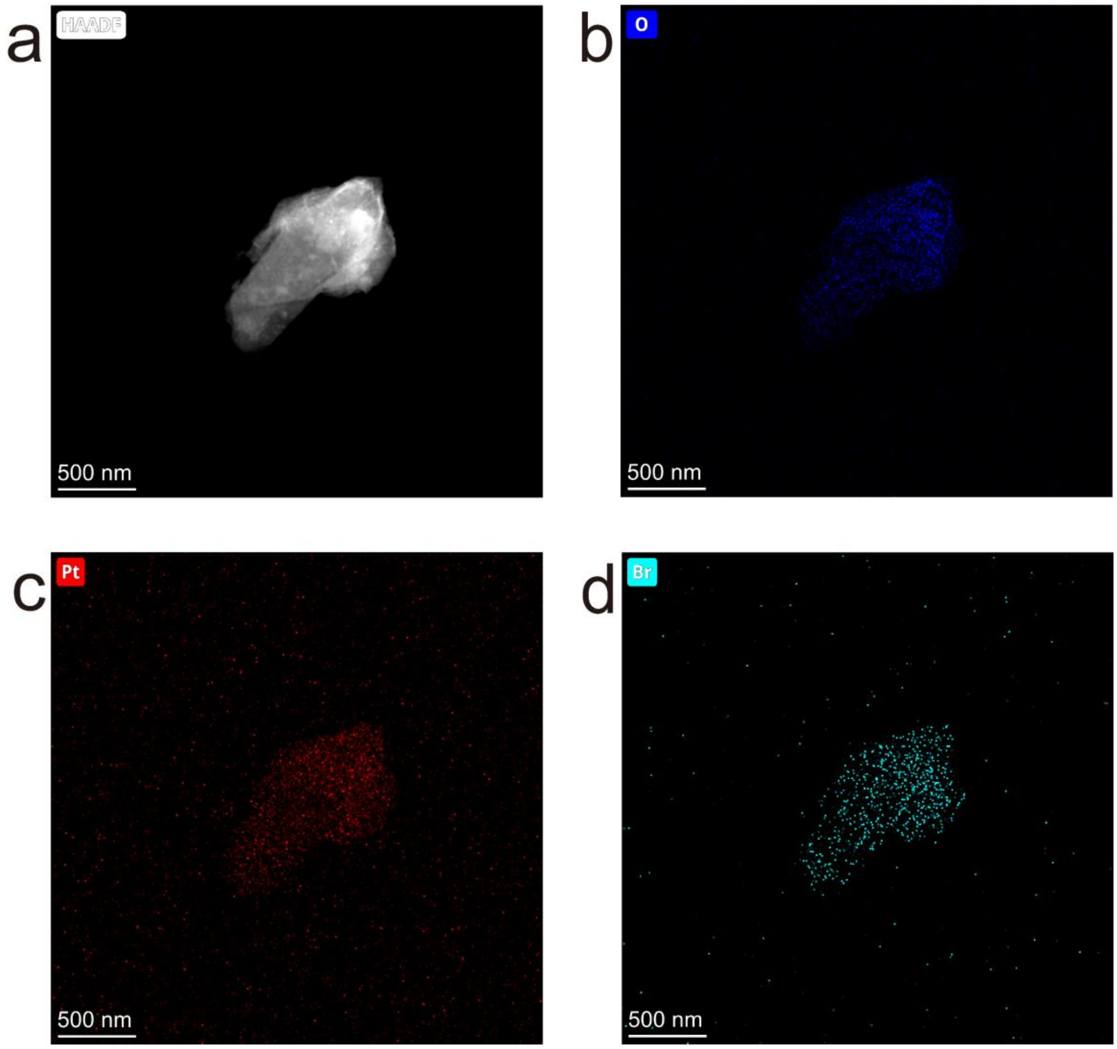


**Supplementary Fig. 30. Elemental mapping image of R-Br-Pt/LDH.** (a) HAADF-STEM image and corresponding elemental mapping image of (b) O, (c) Pt, and (d) Br.

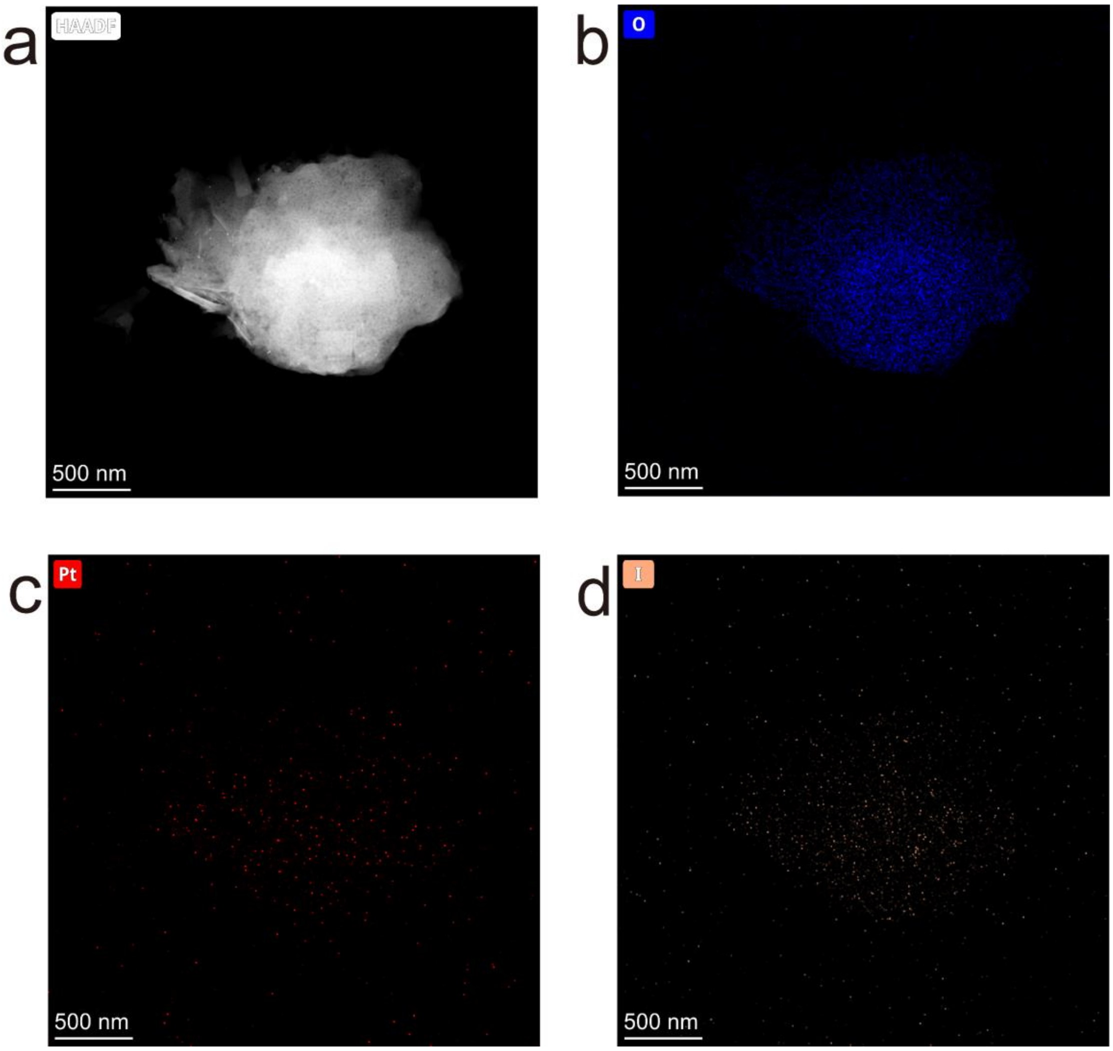


**Supplementary Fig. 31. Elemental mapping image of R-I-Pt/LDH.** (a) HAADF-STEM image and corresponding elemental mapping image of (b) O, (c) Pt, and (d) I.

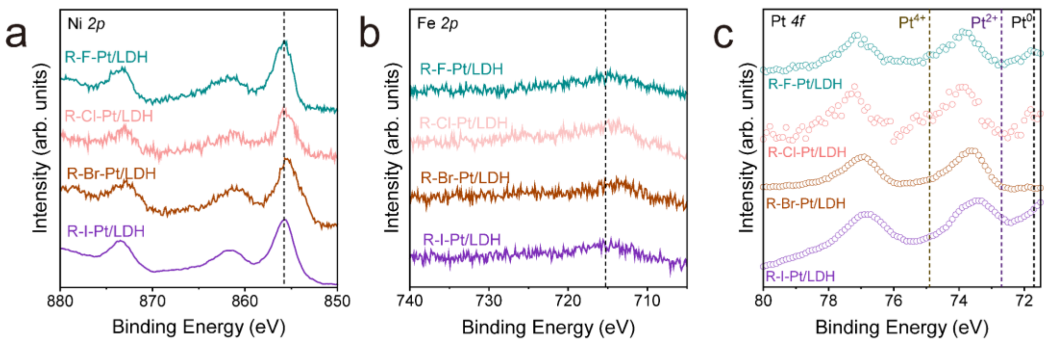


**Supplementary Fig. 32. Valence state characterizations of reversed catalysts.** High resolution (a) Fe *2p*, (b) Ni *2p*, and (c) Pt *4f* XPS spectra for R-F-Pt/LDH, R-Cl-Pt/LDH, R-Br-Pt/LDH, and R-I-Pt/LDH, respectively. All the valence state of Pt in the reversed samples are between +2 to +4, and the order from highest to lowest is: R-Cl-Pt/LDH > R-F-Pt/LDH > R-Br-Pt/LDH > R-I-Pt/LDH.

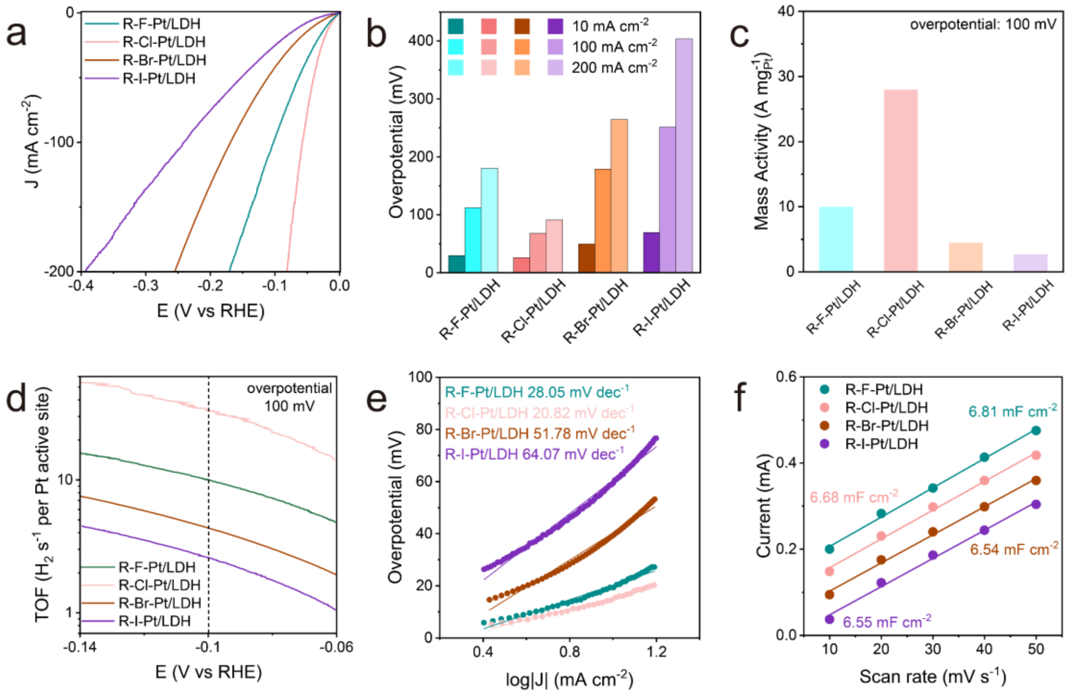


**Supplementary Fig. 33. HER performance of reversed catalysts.** (a) HER polarization curves of the Pt-SACs with different axial-ligands. (b) Overpotentials required to achieve current densities of 10, 50, and 100 mA cm$^{-2}$ for the Pt-SACs with different axial-ligands. (c) The mass activity, (d) TOFs plots, (e) Tafel analysis, and (f) electrochemical double-layer capacity of the Pt-SACs with different axial-ligands.

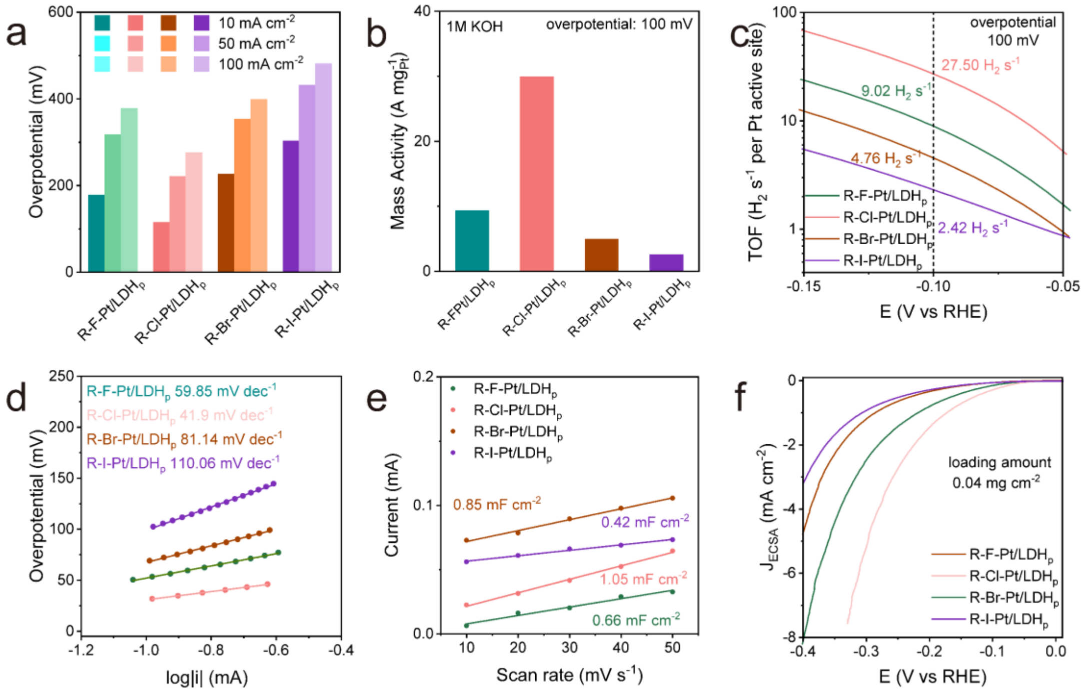


**Supplementary Fig. 34. HER performance of powder-based reversed catalysts.** (a) Overpotentials required to achieve current densities of 10, 50, and 100 mA $cm^{-2}$ for the powder-based Pt-SACs with different axial-ligands. (b) The mass activity, (c) TOFs plots, and (d) Tafel analysis, (e) electrochemical double-layer capacity and (f) ECSA normalized HER polarization curves of the powder-based Pt-SACs with different axial-ligands.

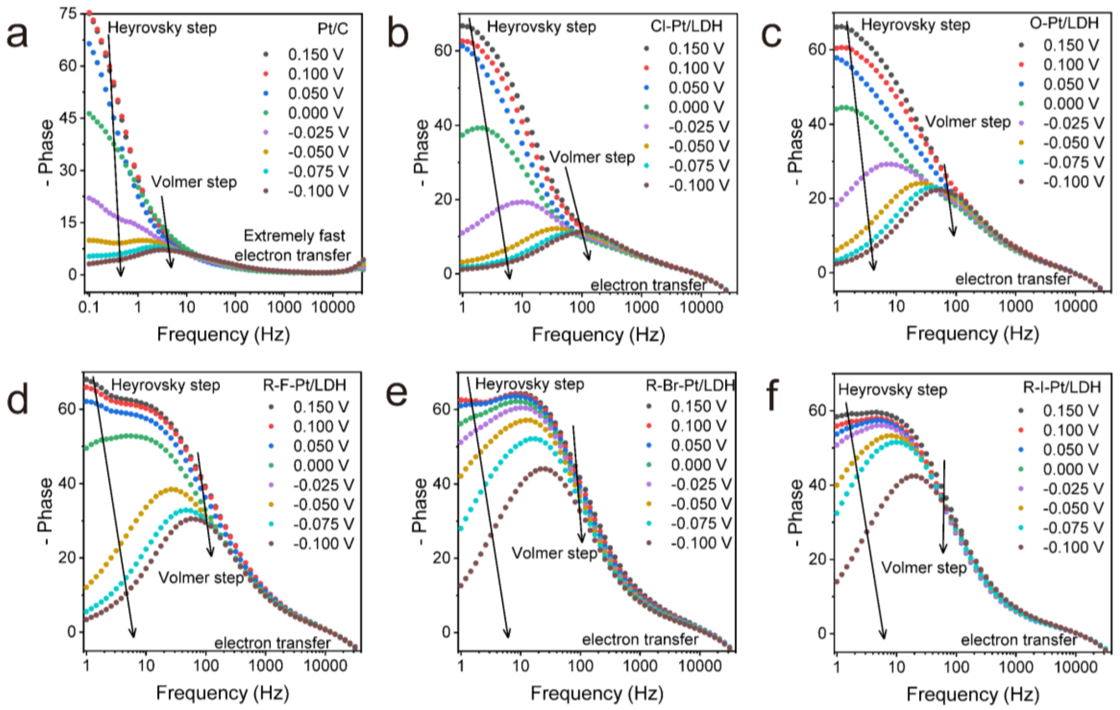


**Supplementary Fig. 35. Bode plots for operando EIS measurements.** Bode plots for (a) Pt/C, (b) Cl-Pt/LDH, (c) HO-Pt/LDH, (d) R-F-Pt/LDH, (e) R-Br-Pt/LDH, and (f) R-I-Pt/LDH. Note, two peaks and one trough from low frequency to high can be found in all Bode plots, demonstrating three state variables exist, corresponding to the Heyrovsky step, Volmer step and electron transfer step, respectively.

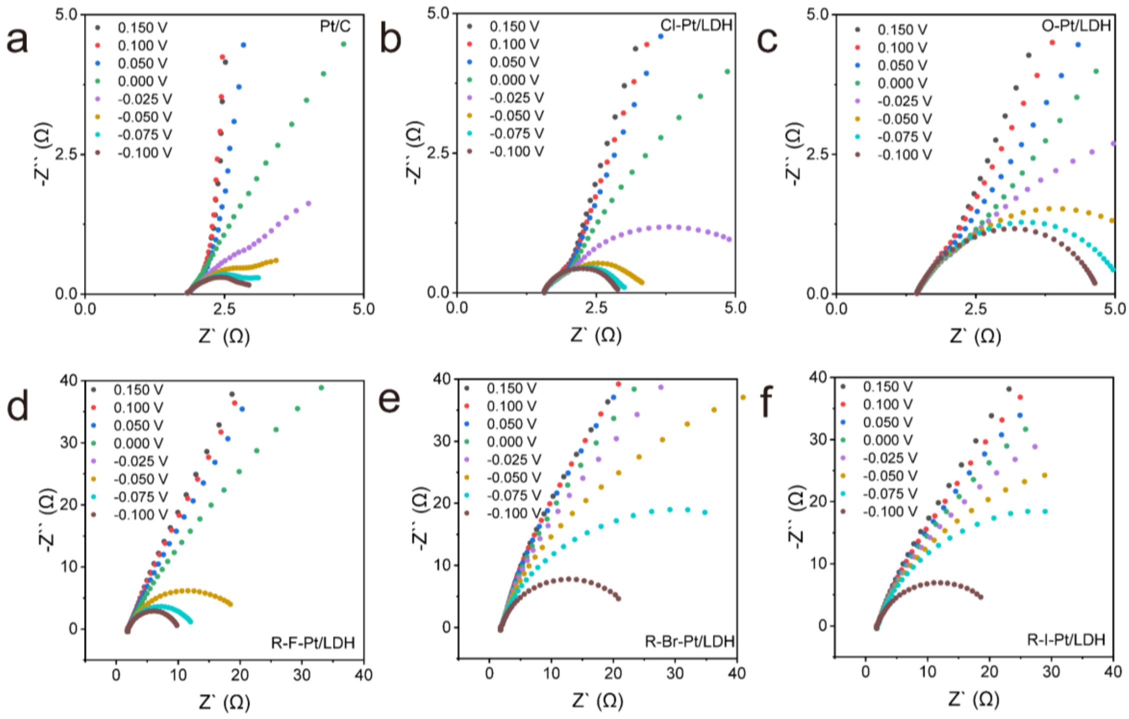


**Supplementary Fig. 36. Nyquist plots for operando EIS measurements.** Nyquist plots for (a) Pt/C, (b) Cl-Pt/LDH, (c) HO-Pt/LDH, (d) R-F-Pt/LDH, (e) R-Br-Pt/LDH, and (f) R-I-Pt/LDH, respectively.

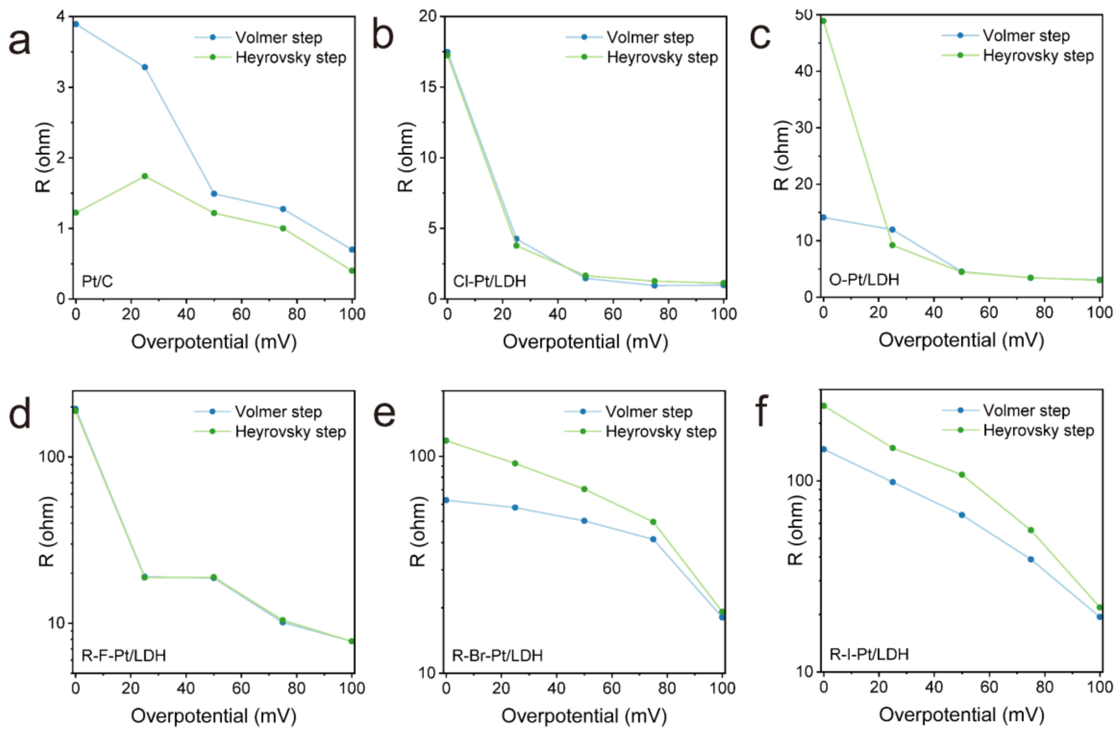


**Supplementary Fig. 37. Correlation of the resistance to potential:** (a) Pt/C, (b) Cl-Pt/LDH, (c) HO-Pt/LDH, (d) R-F-Pt/LDH, (e) R-Br-Pt/LDH, and (f) R-I-Pt/LDH, respectively.

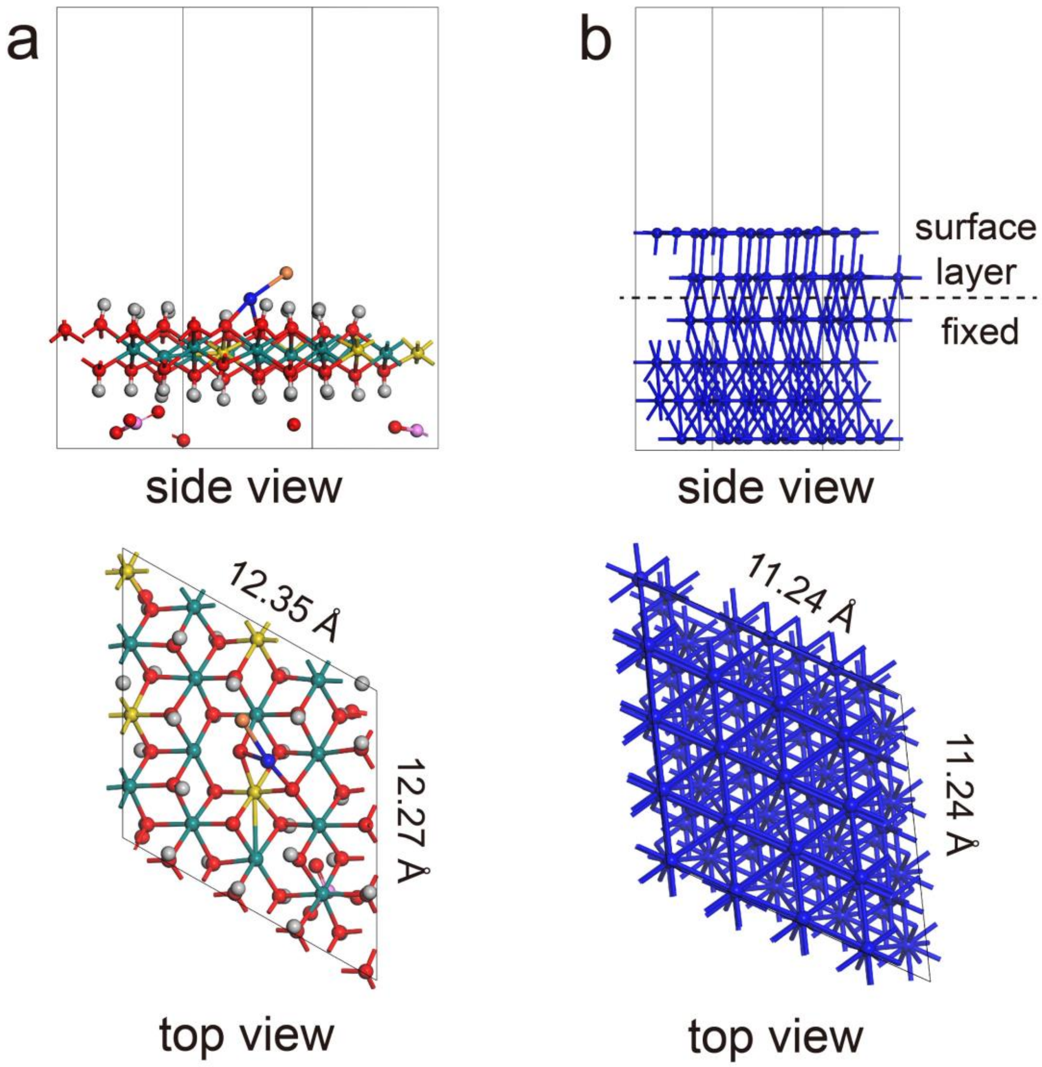


**Supplementary Fig. 38. Computational models for the Pt-SACs.** Top and side views of slab models for (a) Pt-SACs and (b) Pt (111). The blue, olive, yellow, red, and orange sphere refer to Pt, Ni, Fe, O atoms and X group (X = −F, −Cl, −Br, −I, and −OH), respectively. During the optimization, the atoms of Pt (111) in the slab bottom (below the dash line) are fixed at bulk position while the other layers are relaxed.

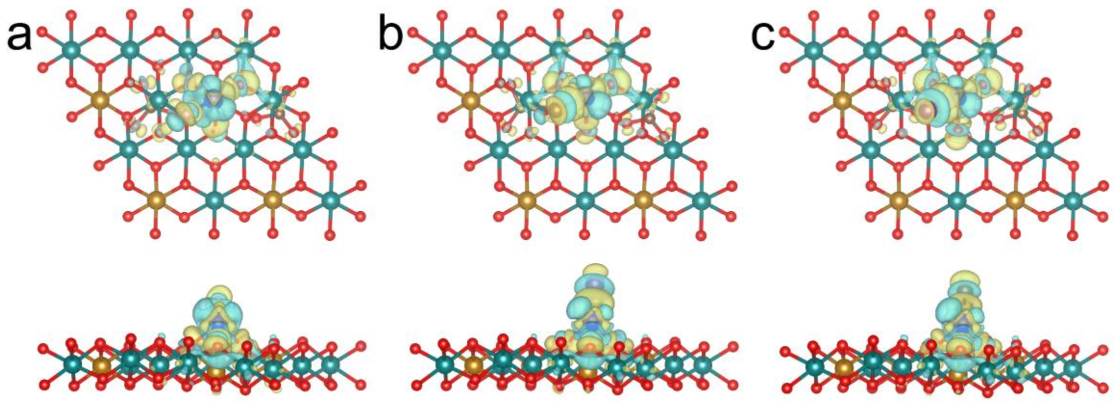


**Supplementary Fig. 39. Computational models and localized electric field distribution.** Top and side views of (a) R-F-Pt/LDH, (b) R-Br-Pt/LDH, (c) R-I-Pt/LDH. The blue, olive, yellow, red, pink, brown, and purple sphere refer to Pt, Ni, Fe, O, F, Br, and I atoms, respectively.

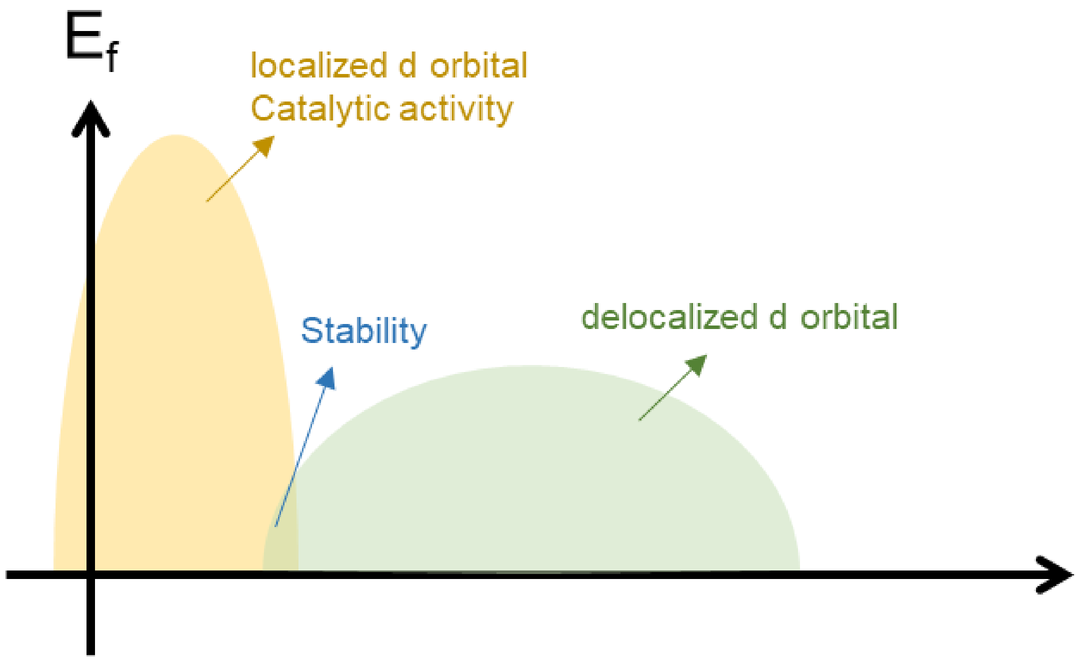


**Supplementary Fig. 40. Illustration of delocalized and localized d orbitals.** According to the energy position, the d-orbital of Pt in Pt SACs can be divided into two types: the delocalized d orbitals far from the fermi level; and the localized orbitals near the fermi level. The delocalized d orbitals are formed by the interaction with the p orbitals of the coordinated O atoms on NiFe-LDH. The filled electron in these orbitals makes the entire system lower energy and steadier, which is the key to make the Pt atomic dispersed. To the contrary, the electron on the localized d orbitals of Pt can be considered as the isolated electrons to integrate with the reaction intermediate, which is considered as the essential origin of the SAC activity. The distribution of two kinds of d orbitals can be regulated due to the different interaction between the ligand and the d orbitals of Pt[12].

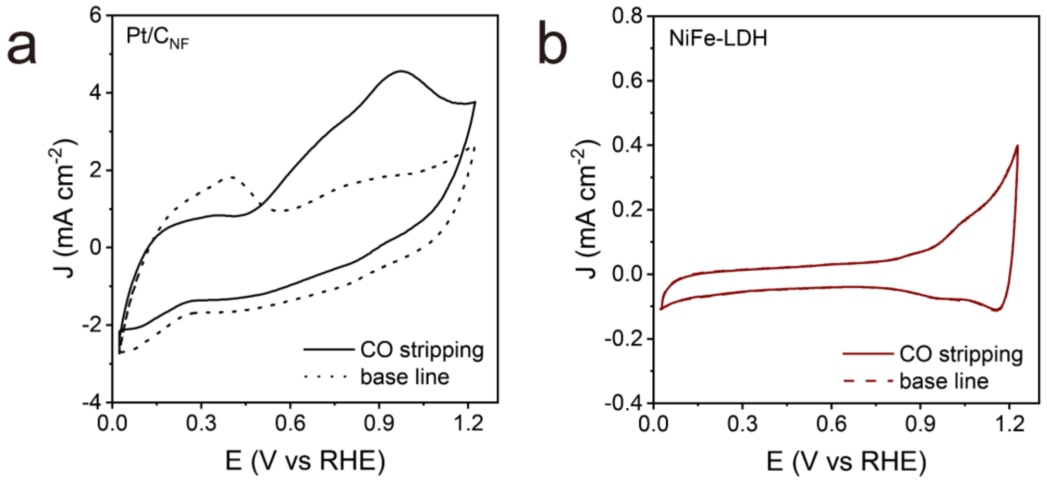


**Supplementary Fig. 41. CO stripping measurement for the control sample.** The CO stripping voltammetry of (a) Pt/C, and (b) NiFe-LDH.

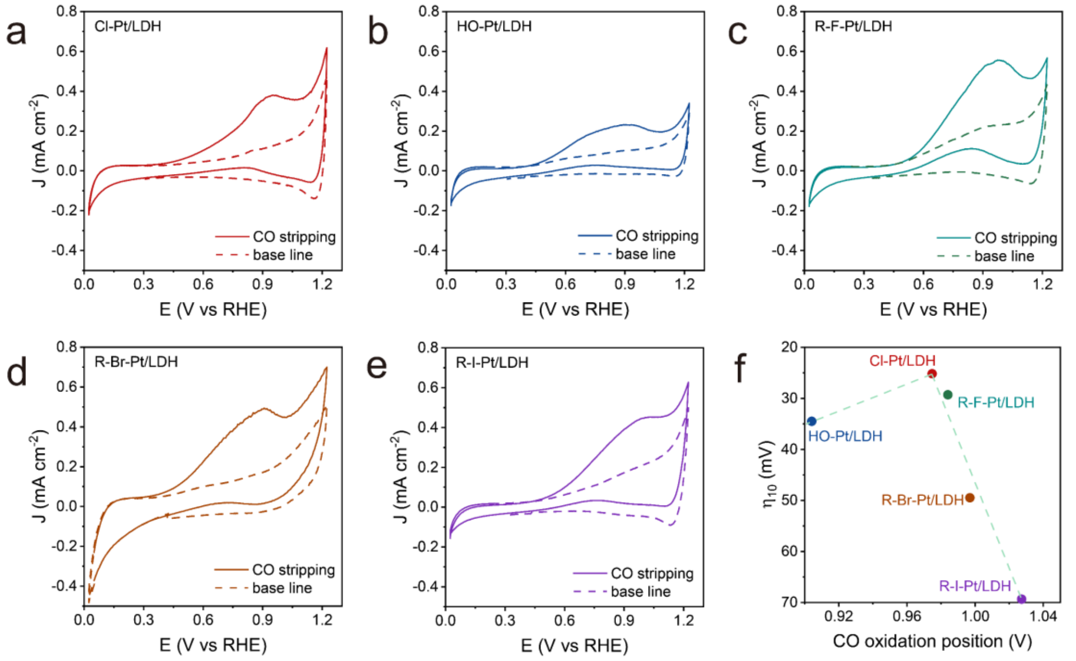


**Supplementary Fig. 42. CO stripping measurement for the obtained catalysts.** The CO stripping voltammetry of (a) Cl-Pt/LDH, (b) HO-Pt/LDH, (c) R-F-Pt/LDH, (d) R-Br-Pt/LDH, and (e) R-I-Pt/LDH. (f) Relationship of alkaline HER activity and CO oxidation position peaks. It is well known that water dissociation can be facilitated by good interaction between *OH intermediates and the catalytic surface. In addition, such interaction can be monitored using CO stripping tests[13-16].

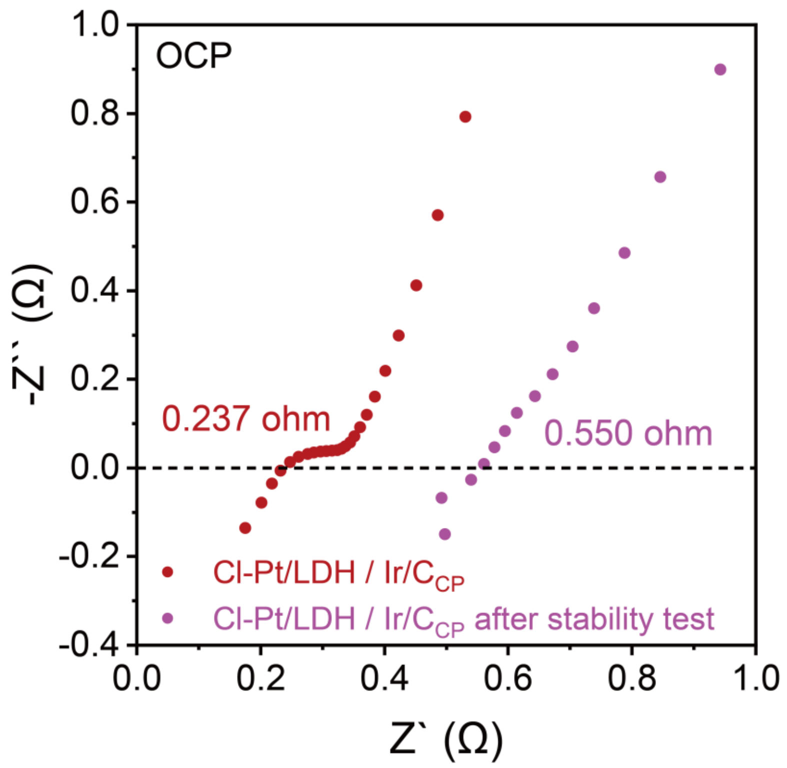


**Supplementary Fig. 43. Impendance of MEA electrolysers.** EIS Nyquist plots of Cl-Pt/LDH / Ir/C before and after the stability test. Note the solution resistance increased significantly after the stability test, which may because the unstable anionic membrane. Also, the anode catalysts were not fully optimized to investigate the maximum long-term stability of the real device.

**Supplementary Tables.**

**Supplementary Table 1. The contents of Pt loading in the obtained samples**.

| Samples | Pt loadings[b] (wt.%) |
|---|---|
| Cl-Pt/LDH | 0.72 |
| Post-catalysis Cl-Pt-LDH[a] | 0.70 |
| HO-Pt/LDH | 0.72 |
| R-Cl-Pt/LDH | 0.75 |
| R-F-Pt/LDH | 0.71 |
| R-Br-Pt/LDH | 0.73 |
| R-I-Pt/LDH | 0.73 |
| $Pt_{np}$/LDH | 7.1 |

[a]the Pt loading of the used sample after 20 h stability test.

[b]the Pt loading is determined by ICP-AES.

**Supplementary Table 2. EXAFS fitting parameters[a] at the Pt L-edge.**

| Samples | Path | *C.N.* | *R* (Å) | $\sigma^2$*10$^3$ (Å$^2$) | *ΔE* (eV) | *R* |
|---|---|---|---|---|---|---|
| Pt foil | Pt-$Pt_1$ | 12* | 2.76±0.004 | 4.63±0.48 | 3.22±0.66 | 0.009 |
| | Pt-$Pt_2$ | 6* | 3.92±0.012 | 4.66±1.22 | | |
| $PtO_2$ | Pt-O | 5.72±0.71 | 2.02±0.010 | 2.24±1.35 | 9.40±0.64 | 0.012 |
| | Pt-Pt | 7.71±1.03 | 3.11±0.013 | 4.28±1.56 | | |
| $K_2PtCl_4$ | Pt-Cl | 3.95±0.41 | 2.31±0.008 | 2.61±1.01 | 4.11±1.17 | 0.033 |
| Cl-Pt/LDH | Pt-O | 3.00±0.42 | 2.01±0.016 | 6.44±2.41 | 9.73±1.55 | 0.022 |
| | Pt-Cl | 0.48±0.25 | 2.35±0.034 | 4.79±6.42 | | |
| HO-Pt/LDH | Pt-O | 3.60±0.53 | 2.01±0.012 | 6.59±1.99 | 7.87±1.48 | 0.027 |

[a]*C.N.*: coordination number; *R*: distance between absorber and backscatter atoms; $\sigma^2$: Debye-Waller factor to account for both thermal and structural disorders; *ΔE*: the inner potential correction. *R* factor (%): goodness of fit. *The experimental EXAFS fit of metal foil by fixing *C.N.* as the known crystallographic value. $S_0^2$ was fixed to 0.8 as determined from Pt foil fitting. Fitting range: 3.0≤$k$ (Å$^{-1}$)≤12 and 1≤$R$ (Å)≤3.

**Supplementary Table 3. Comparison of alkaline HER activity.**

| Catalysts | $\eta_{10}$ (mV) | $\eta_{100}$ (mV) | Tafel (mV $dec^{-1}$) | Charge transfer resistance (ohm) | Mass activity at certain $\eta$ (A $mg_{pt}^{-1}$ @ mV) | Turnover frequency at certain $\eta$ ($H_2$ $s^{-1}$ @ mV) | Ref. |
|---|---|---|---|---|---|---|---|
| Cl-Pt/LDH | 25.2 | 51.9 | 24.33 | 21.15 | 30.6@100 | 30.3@100 | This work |
| HO-Pt/LDH | 34.5 | 142.5 | 29.98 | 28.45 | 6.6@100 | 5.1@100 | This work |
| $Pt_1$/N-C[1] | 46 | 203 | 36.8 | - | - | 1.89@50 | Ref. 1 |
| SANi-PtNWs[17] | 70 | - | 60.3 | - | 11.8@70 | - | Ref. 17 |
| Pt-SAs/$MoSe_2$[18] | 29 | 120 | 41 | - | 34.4@100 | 6.21@50 | Ref. 18 |
| $Pt_{np}$/$Co_{0.85}Se$[19] | 58 | 164 | 39 | - | 1.28@100 | 3.93@100 | Ref. 19 |
| Pt@DG[20] | 37 | 236 | 119 | - | 6.78@100 | 6.73@50 | Ref. 20 |
| $Pt_{SA}$-NiO/Ni[21] | 26 | 86 | 27.07 | 0.61 | 20.6@100 | 5.71@50 | Ref. 21 |
| $Pt_{SA}$-$Co(OH)_2$@Ag NWs[22] | 29 | 104 | 35.72 | 0.7 | 1.6@29 | - | Ref. 22 |
| $PtSe_2$/Pt[23] | 42 | - | 53 | 3.4 | - | - | Ref. 23 |
| Pt–Ni NTAs[24] | 23 | 56 | 38 | 0.22 | 4.27@50 | - | Ref. 24 |
| $Pt_5$/HMCS[25] | 46.2 | 127 | 28.4 | - | 3.23@70 | - | Ref. 25 |
| $Pt_3Co$@NCNT[26] | 36 | 100 | 27.2 | - | - | 0.94@100 | Ref. 26 |
| A-CoPt-NC[27] | 27 | - | 50 | - | 45@70 | - | Ref. 27 |
| Pt/Ni ASs[28] | 28 | - | 47 | - | 30.2@100 | 11.4@50 | Ref. 28 |
| Pt-PVP/TNR@GC[29] | 21 | 62 | 34 | - | 16.53@50 | - | Ref. 29 |
| $Ti_3C_2T_x$-$Pt_{SA}$[30] | 38 | 141 | 45 | - | 23.32@100 | 23.45@100 | Ref. 30 |
| N,Pt-$MoS_2$[31] | 38 | - | 39 | 6.9 | 20.2@100 | 2.4@100 | Ref. 31 |

**Supplementary Table 4. Optimum impedance fit parameters for Pt/C.**

| $\eta^{a}$ | $R_s$ | $CPE_2T$ | $CPE_2P$ | $R_2$ | $CPE_3T$ | $CPE_3P$ | $R_3$ |
|---|---|---|---|---|---|---|---|
| 0 | 1.825 | 0.276 | 0.517 | 3.892 | 0.288 | 0.617 | 1.222 |
| 25 | 1.827 | 0.360 | 0.457 | 3.281 | 0.229 | 0.633 | 1.741 |
| 50 | 1.832 | 0.230 | 0.528 | 1.491 | 0.180 | 0.661 | 1.217 |
| 75 | 1.836 | 0.195 | 0.547 | 1.274 | 0.149 | 0.689 | 1.000 |
| 100 | 1.816 | 0.110 | 0.564 | 0.699 | 0.002 | 0.757 | 0.399 |

[a]The unit of $\eta$ is mV.

**Supplementary Table 5. Optimum impedance fit parameters for Cl-Pt/LDH.**

| $\eta$ | $R_s$ | $CPE_1T$ | $CPE_1P$ | $R_1$ | $CPE_2T$ | $CPE_2P$ | $R_2$ | $CPE_3T$ | $CPE_3P$ | $R_3$ |
|---|---|---|---|---|---|---|---|---|---|---|
| 0 | 1.535 | 0.039 | 0.574 | 2.017 | 0.026 | 0.744 | 17.47 | 0.023 | 0.801 | 17.25 |
| 25 | 1.539 | 0.023 | 0.631 | 1.604 | 0.032 | 0.66 | 4.242 | 0.026 | 0.714 | 3.775 |
| 50 | 1.553 | 0.011 | 0.72 | 1.284 | 0.011 | 0.786 | 1.463 | 0.015 | 0.726 | 1.649 |
| 75 | 1.558 | 0.010 | 0.729 | 1.341 | 0.003 | 0.944 | 0.95 | 0.008 | 0.794 | 1.255 |
| 100 | 1.561 | 0.005 | 0.803 | 1.003 | 0.003 | 0.912 | 0.981 | 0.006 | 0.835 | 1.127 |

[a]The unit of $\eta$ is mV.

**Supplementary Table 6. Optimum impedance fit parameters for HO-Pt/LDH.**

| $\eta$ | $R_s$ | $CPE_1T$ | $CPE_1P$ | $R_1$ | $CPE_2T$ | $CPE_2P$ | $R_2$ | $CPE_3T$ | $CPE_3P$ | $R_3$ |
|---|---|---|---|---|---|---|---|---|---|---|
| 0 | 1.451 | 0.008 | 0.740 | 1.789 | 0.028 | 0.576 | 14.13 | 0.022 | 0.685 | 48.87 |
| 25 | 1.446 | 0.006 | 0.777 | 1.731 | 0.025 | 0.577 | 11.98 | 0.016 | 0.741 | 9.215 |
| 50 | 1.445 | 0.004 | 0.808 | 1.612 | 0.01 | 0.708 | 4.532 | 0.009 | 0.764 | 4.47 |
| 75 | 1.444 | 0.003 | 0.839 | 1.212 | 0.006 | 0.777 | 3.416 | 0.006 | 0.812 | 3.465 |
| 100 | 1.45 | 0.002 | 0.868 | 0.977 | 0.005 | 0.803 | 3.073 | 0.004 | 0.844 | 2.986 |

[a]The unit of $\eta$ is mV.

**Supplementary Table 7. Optimum impedance fit parameters for R-F-Pt/LDH.**

| $\eta$ | $R_s$ | $CPE_1T$ | $CPE_1P$ | $R_1$ | $CPE_2T$ | $CPE_2P$ | $R_2$ | $CPE_3T$ | $CPE_3P$ | $R_3$ |
|---|---|---|---|---|---|---|---|---|---|---|
| 0 | 1.921 | 0.001 | 0.899 | 1.598 | 0.005 | 0.707 | 194.5 | 0.005 | 0.708 | 188.7 |
| 25 | 1.933 | 0.001 | 0.913 | 1.462 | 0.004 | 0.727 | 19.06 | 0.004 | 0.744 | 18.84 |
| 50 | 1.967 | 0.001 | 0.925 | 1.407 | 0.004 | 0.741 | 18.68 | 0.004 | 0.74 | 18.95 |
| 75 | 1.988 | 0.001 | 0.914 | 1.499 | 0.003 | 0.779 | 10.11 | 0.003 | 0.77 | 10.43 |
| 100 | 2.006 | 0.001 | 0.89 | 1.797 | 0.002 | 0.804 | 7.79 | 0.002 | 0.811 | 7.795 |

[a]The unit of $\eta$ is mV.

**Supplementary Table 8. Optimum impedance fit parameters for R-Br-Pt/LDH.**

| $\eta$ | $R_s$ | $CPE_1T$ | $CPE_1P$ | $R_1$ | $CPE_2T$ | $CPE_2P$ | $R_2$ | $CPE_3T$ | $CPE_3P$ | $R_3$ |
|---|---|---|---|---|---|---|---|---|---|---|
| 0 | 1.878 | 0.003 | 0.832 | 1.83 | 0.004 | 0.809 | 62.71 | 0.005 | 0.766 | 118 |
| 25 | 1.875 | 0.003 | 0.833 | 1.928 | 0.004 | 0.81 | 58.03 | 0.005 | 0.767 | 92.57 |
| 50 | 1.872 | 0.003 | 0.832 | 1.923 | 0.004 | 0.811 | 50.35 | 0.005 | 0.773 | 70.49 |
| 75 | 1.871 | 0.003 | 0.824 | 1.97 | 0.004 | 0.812 | 41.41 | 0.004 | 0.793 | 49.77 |
| 100 | 1.803 | 0.003 | 0.803 | 3.798 | 0.004 | 0.807 | 18.1 | 0.004 | 0.800 | 19.19 |

[a]The unit of $\eta$ is mV.

**Supplementary Table 9. Optimum impedance fit parameters for R-I-Pt/LDH.**

| $\eta$ | $R_s$ | $CPE_1T$ | $CPE_1P$ | $R_1$ | $CPE_2T$ | $CPE_2P$ | $R_2$ | $CPE_3T$ | $CPE_3P$ | $R_3$ |
|---|---|---|---|---|---|---|---|---|---|---|
| 0 | 1.828 | 0.003 | 0.786 | 2.687 | 0.002 | 0.835 | 146.1 | 0.003 | 0.774 | 246.4 |
| 25 | 1.821 | 0.003 | 0.766 | 5.164 | 0.002 | 0.840 | 98.22 | 0.003 | 0.785 | 148.5 |
| 50 | 1.817 | 0.004 | 0.761 | 4.408 | 0.002 | 0.833 | 66.26 | 0.003 | 0.754 | 107.6 |
| 75 | 1.819 | 0.003 | 0.805 | 2.575 | 0.003 | 0.829 | 38.8 | 0.003 | 0.756 | 55.07 |
| 100 | 1.818 | 0.003 | 0.779 | 2.778 | 0.003 | 0.819 | 19.38 | 0.003 | 0.794 | 21.76 |

[a]The unit of $\eta$ is mV.

**Supplementary Table 10. Reaction energies[a] for the alkaline HER.**

| Model | $*H_2O$ | TS:H-OH | *H *OH | $\Delta G_{H*}$ | $\Delta G_{OH*}$ | $\Delta G_{H2O}$ |
|---|---|---|---|---|---|---|
| Cl-Pt | -0.347 | 0.296 | -0.413 | -0.34 | 0.073 | -0.643 |
| F-Pt | -0.58 | 0.111 | -0.521 | -0.729 | -0.208 | -0.691 |
| O-Pt | -0.519 | 0.595 | -1.374 | -0.965 | 0.409 | -1.114 |
| Br-Pt | -0.311 | 0.253 | -0.586 | -0.968 | -0.382 | -0.564 |
| I-Pt | -0.801 | -0.13 | -0.569 | -1.006 | -0.441 | -0.671 |
| Pt(111) | 0.259 | 1.287 | 0.889 | -0.303 | -1.192 | -1.028 |

[a]The energy unit of all the variables is eV.

## Supplementary References


1. Fang S., *et al.* Uncovering near-free platinum single-atom dynamics during electrochemical hydrogen evolution reaction. *Nat. Commun.* **11**, 1029 (2020).
2. Tsyganok A., *et al.* Operando X-ray Absorption Spectroscopy (XAS) Observation of Photoinduced Oxidation in FeNi (Oxy)hydroxide Overlayers on Hematite (α-$Fe_2O_3$) Photoanodes for Solar Water Splitting. *Langmuir.* **36**, 11564-11572 (2020).
3. Bowen D. K., Tanner B. K., Wormington M., Panaccione C., Matney K. M., Bowen D. K. Characterization of structures from X-ray scattering data using genetic algorithms. *Philos. Trans. Royal Soc. A.* **357**, 2827-2848 (1999).
4. Qiao B., *et al.* Single-atom catalysis of CO oxidation using $Pt_1/FeO_x$. *Nat. Chem.* **3**, 634-641 (2011).
5. Funke H., Scheinost A. C., Chukalina M. Wavelet analysis of extended x-ray absorption fine structure data. *Phys. Rev. B.* **71**, 094110 (2005).
6. Hussain S., *et al.* Improved ORR Activity and Long-Term Durability of Pt Nanoparticles Deposited on $TiO_2$-Decorated Multiwall Carbon Nanotubes. *J. Electrochem. Soc.* **166**, F1284-F1291 (2019).
7. Xu W., Lu Z., Sun X., Jiang L., Duan X. Superwetting Electrodes for Gas-Involving Electrocatalysis. *Acc. Chem. Res.* **51**, 1590-1598 (2018).
8. Yang T. T., Tan T. L., Saidi W. A. High Activity toward the Hydrogen Evolution Reaction on the Edges of $MoS_2$-Supported Platinum Nanoclusters Using Cluster Expansion and Electrochemical Modeling. *Chem. Mater.* **32**, 1315-1321 (2020).
9. Subbaraman R., *et al.* Enhancing Hydrogen Evolution Activity in Water Splitting by Tailoring $Li^+$-$Ni(OH)_2$-Pt Interfaces. *Science.* **334**, 1256-1260 (2011).
10. Danilovic N., *et al.* Enhancing the Alkaline Hydrogen Evolution Reaction Activity through the Bifunctionality of $Ni(OH)_2$/Metal Catalysts. *Angew. Chem. Int. Ed.* **51**, 12495-12498 (2012).
11. Chen W., *et al.* Deciphering the alternating synergy between interlayer Pt single-atom and NiFe layered double hydroxide for overall water splitting. *Energy Environ. Sci.* **14**, 6428-6440 (2021).
12. Wang Y., *et al.* Recent progress in theoretical and computational investigations of structural stability and activity of single-atom electrocatalysts. *Prog. Nat. Sci.* **29**, 256-264 (2019).
13. Rudi S., Cui C., Gan L., Strasser P. Comparative Study of the Electrocatalytically Active Surface Areas (ECSAs) of Pt Alloy Nanoparticles Evaluated by Hupd and CO-stripping voltammetry. *Electrocatalysis.* **5**, 408-418 (2014).
14. Gasteiger H. A., Markovic N., Ross P. N., Cairns E. J. Carbon monoxide electrooxidation on well-characterized platinum-ruthenium alloys. *J. Phys. Chem.* **98**, 617-625 (1994).
15. Subbaraman R., *et al.* Trends in activity for the water electrolyser reactions on 3d M(Ni,Co,Fe,Mn) hydr(oxy)oxide catalysts. *Nat. Mater.* **11**, 550-557 (2012).
16. Ochal P., *et al.* CO stripping as an electrochemical tool for characterization of Ru@Pt core-shell catalysts. *J. Electroanal. Chem.* **655**, 140-146 (2011).

17. Li M., *et al.* Single-atom tailoring of platinum nanocatalysts for high-performance multifunctional electrocatalysis. *Nat. Catal.* **2**, 495-503 (2019).
18. Shi Y., *et al.* Electronic metal–support interaction modulates single-atom platinum catalysis for hydrogen evolution reaction. *Nat. Commun.* **12**, 3021 (2021).
19. Jiang K., *et al.* Single platinum atoms embedded in nanoporous cobalt selenide as electrocatalyst for accelerating hydrogen evolution reaction. *Nat. Commun.* **10**, 1743 (2019).
20. Yang Q., *et al.* Single Carbon Vacancy Traps Atomic Platinum for Hydrogen Evolution Catalysis. *J. Am. Chem. Soc.* **144**, 2171-2178 (2022).
21. Zhou K. L., *et al.* Platinum single-atom catalyst coupled with transition metal/metal oxide heterostructure for accelerating alkaline hydrogen evolution reaction. *Nat. Commun.* **12**, 3783 (2021).
22. Zhou K. L., *et al.* Seamlessly conductive $Co(OH)_2$ tailored atomically dispersed Pt electrocatalyst with a hierarchical nanostructure for an efficient hydrogen evolution reaction. *Energy Environ. Sci.* **13**, 3082-3092 (2020).
23. Wang Z., *et al.* $PtSe_2$/Pt Heterointerface with Reduced Coordination for Boosted Hydrogen Evolution Reaction. *Angew. Chem. Int. Ed.* **60**, 23388-23393 (2021).
24. Nairan A., *et al.* Proton selective adsorption on Pt–Ni nano-thorn array electrodes for superior hydrogen evolution activity. *Energy Environ. Sci.* **14**, 1594-1601 (2021).
25. Wan X.-K., Wu H. B., Guan B. Y., Luan D., Lou X. W. Confining Sub-Nanometer Pt Clusters in Hollow Mesoporous Carbon Spheres for Boosting Hydrogen Evolution Activity. *Adv. Mater.* **32**, 1901349 (2020).
26. Zhang S. L., Lu X. F., Wu Z.-P., Luan D., Lou X. W. Engineering Platinum–Cobalt Nano-alloys in Porous Nitrogen-Doped Carbon Nanotubes for Highly Efficient Electrocatalytic Hydrogen Evolution. *Angew. Chem. Int. Ed.* **60**, 19068-19073 (2021).
27. Zhang L., *et al.* Charge Polarization from Atomic Metals on Adjacent Graphitic Layers for Enhancing the Hydrogen Evolution Reaction. *Angew. Chem. Int. Ed.* **58**, 9404-9408 (2019).
28. Ding J., Ji Y., Li Y., Hong G. Monoatomic Platinum-Embedded Hexagonal Close-Packed Nickel Anisotropic Superstructures as Highly Efficient Hydrogen Evolution Catalyst. *Nano Lett.* **21**, 9381-9387 (2021).
29. Li C., *et al.* Polyvinylpyrrolidone-Coordinated Single-Site Platinum Catalyst Exhibits High Activity for Hydrogen Evolution Reaction. *Angew. Chem. Int. Ed.* **59**, 15902-15907 (2020).
30. Zhang J., Wang E., Cui S., Yang S., Zou X., Gong Y. Single-Atom Pt Anchored on Oxygen Vacancy of Monolayer $Ti_3C_2T_x$ for Superior Hydrogen Evolution. *Nano Lett.* **22**, 1398-1405 (2022).
31. Sun Y., *et al.* Plasma-induced large-area N,Pt-doping and phase engineering of $MoS_2$ nanosheets for alkaline hydrogen evolution. *Energy Environ. Sci.* **15**, 1201-1210 (2022).
